# High Spin Iron–Phosphinidene and Arsinidene Complexes With Attenuated Metal–Ligand Multiple Bond Character

**DOI:** 10.1002/anie.202523239

**Published:** 2026-03-19

**Authors:** Austin D. Chivington, Álvaro García‐Romero, David C. Meier, Maren Pink, Jose M. Goicoechea, Jeremy M. Smith

**Affiliations:** ^1^ Department of Chemistry Indiana University Bloomington Indiana USA

**Keywords:** arsinidene, electronic structure, iron, phosphinidene

## Abstract

The MEH_2_ salts (M = Na, K; E = P, As) react with high spin (*S* = 2) PhB(AdIm)_3_FeCl (PhB(AdIm)_3_^–^ = tris(3‐adamantylimidazol‐2‐ylidene)phenylborate) to afford the corresponding PhB(AdIm)_3_FePH_2_ and PhB(AdIm)_3_FeAsH_2_ complexes. Remarkably, these complexes can be deprotonated by benzyl potassium in the presence of equimolar 18‐crown–6 or 2,2,2‐crypt to provide the parent phosphinidene and arsinidene complexes, [PhB(AdIm)_3_FePH]^–^ and [PhB(AdIm)_3_FeAsH]^–^. Structural and spectroscopic characterization, combined with computational investigations, provide evidence for multiconfigurational electronic structures in which the metal–ligand multiple bond character is attenuated by the high spin iron(II) center. Initial reactivity studies reveal the nucleophilic character of the pnictogenidene ligands, which provide phosphinophosphinidene and phosphinoarsinidene products with a chlorodiazaphospholidine electrophile.

## Graphical abstract

Unusually low metal–ligand bond orders are observed in high spin iron(II) pnictogenidene (PH^2–^ and AsH^2–^) complexes. Electronic structure analysis provides evidence for multiconfigurational character that attenuates the metal–ligand multiple bond character normally that is typically associated with pnictogenidene ligands. These complexes can be used to assemble a phosphino–phosphinidene and –arsinidene.

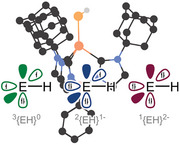

## 1 Introduction

In contrast to imido complexes, the chemistry of transition metal phosphinidenes and arsinidenes (M = ER, E = P, As) is underdeveloped. This is in part a consequence of the inherent properties of the phosphinidene/arsinidene units, which in their free state have an unsaturated valence shell with a triplet ground state that makes them highly reactive [1]. While these species can be tamed by coordination to a transition metal fragment, terminal phosphinidene and arsinidene ligands are rare. Additional stabilization by bulky ligands and/or high oxidation state metals is often required to avoid the formation of bridging complexes [2, 3, 4], as demonstrated by structurally characterized phosphinidene complexes [5, 6, 7, 8, 9, 10, 11, 12, 13, 14, 15, 16, 17, 18, 19, 20, 21, 22, 23, 24, 25, 26, 27, 28, 29, 30, 31]. These complexes adopt low spin states, for which computational analysis suggests the terminally coordinated phosphinidene ligands have a singlet ground state due to π–donation from the ligand substituent or by π–backdonation from the metal [3, 32, 33, 34].

A limited number of platforms that have been shown to stabilize both terminal phosphinidene and arsinidene ligands. Wolczanski reported the Ta(V) complexes (silox)_3_Ta═EPh (E = P, As) in which the pnictogenidene ligands are coordinated to a metal in its highest oxidation state [8], while more recently Reiβ and Hering–Junghans reported the Ti complexes Cp_2_Ti(PMe_3_)(═E^R^ter) (E = P, R = Mes = 2,4,6‐Me‐C_6_H_2_; E = As; R = Dipp = 2,6–^i^Pr_2_C_6_H_3_) that are stabilized by highly protective terphenyl ligands [30]. Interestingly, an antiferromagnetically coupled biradical resonance (Ti^III–^ER^–^) was proposed to have a significant contribution to the ground state of this complex, hinting at a greater diversity of electronic structures for pnictogenidene ligands.

**FIGURE 1:**
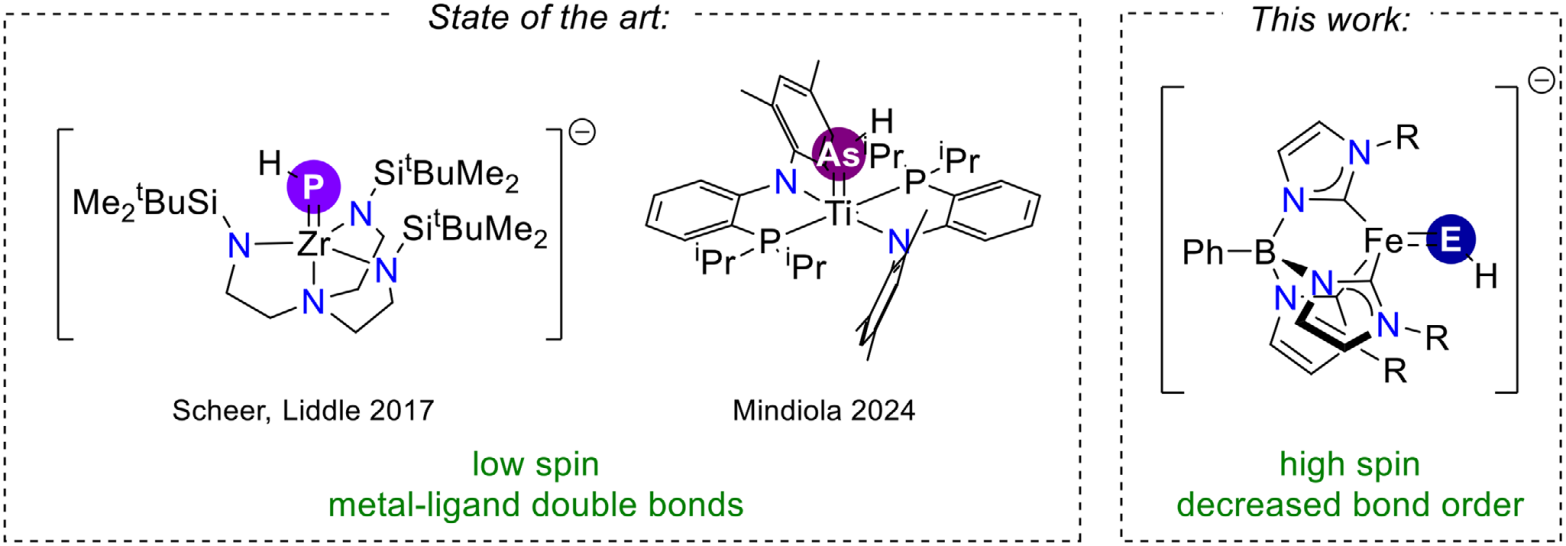
The only two structurally characterized parent phosphinidene and arsinidene transition metal complexes are low spin with a metal‐ligand double bond. The complexes in this paper represent a different bonding paradigm for these ligands.

While sterically protected phosphinidene and arsinidene complexes have been known for at least four decades, those containing these ligands in their parent form, that is, [PH]^2–^ and [AsH]^2–^ are less common. In addition to some spectroscopically characterized complexes [35, 36], only one transition metal (Zr) [1] and two actinide (U, Th) [37, 38] complexes with the parent phosphinidene ligand have been structurally characterized (Figure 1). Complexes with the parent arsinidene ligand are similarly rare [35], where there is likewise only one example of a structurally characterized transition metal complex (Ti) [39] and two for the actinides (U, Th) [1, 38]. All of these complexes are low spin with a metal‐pnictogenidene multiple bond.

We have demonstrated the aptitude of strongly donating and bulky tris(carbene)borate ligands to permit the formation of metal–ligand multiple bonds [40, 41], particularly with iron [42, 43, 44, 45, 46, 47]. Of particular relevance here is the ability of tris(carbene)borates to stabilize iron imides [48, 49], an isolobal sibling of phosphinidenes and arsinidenes. Given this precedent, the increasing interest in the chemistry of multiply bonded metal–pnictogens across the periodic table, and their potential synthetic utility (e.g., in group transfer chemistry) [50, 51, 52, 53], we sought to prepare iron pnictogenidene complexes based on a bulky tris(carbene)borate ligand platform. Previous attempts to synthesize low‐coordinate iron pnictogenidene complexes have been thwarted by the high reactivity of these species [54].

We describe herein the synthesis and characterization of four–coordinate iron(II) tris(carbene)borate complexes in which parent phosphinidene (PH^2–^) and arsinidene (AsH^2–^) ligands are bound to a single metal ion. As well as extending the occurrence of these ligands to include the late transition metals, these complexes are distinct for the high spin configuration of the metal center. Electronic structure calculations provide evidence that this attenuates the metal–ligand multiple bonding, with three configurations contributing to the ground electronic state. This is manifested in the ability of such compounds to act as phosphinidene (PH^2–^) and arsinidene (AsH^2–^) transfer reagents.

## 2 Results and Discussion

### 2.1 Synthesis of Dihydrophosphanide and Dihydroarsanide Complexes

**SCHEME 1:**
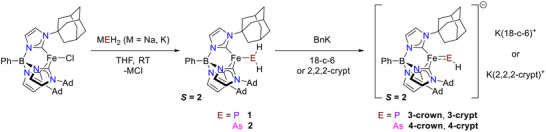
Synthesis of phosphinidene and arsinidene complexes.

**FIGURE 2:**
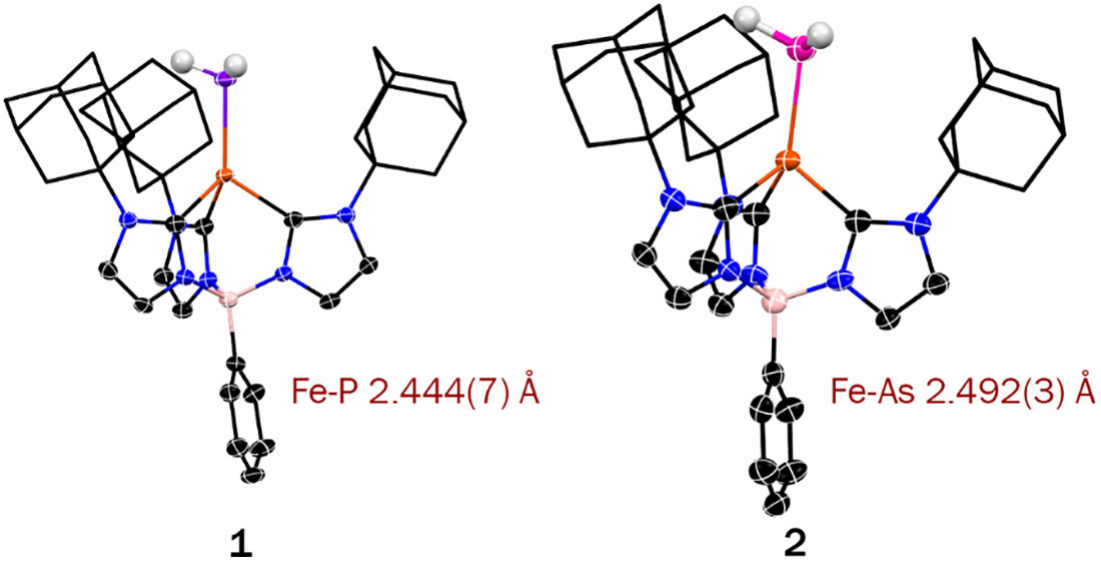
Molecular structures of phosphide and arsenide complexes **1** and **2** from single crystal x‐ray diffraction, shown as ORTEPs. Thermal ellipsoids are shown at 50%, probability, most hydrogen atoms omitted and most of the tris(carbene)borate ligand shown as wireframe for clarity. Black, orange, magenta, blue, pink, red, purple, and fuchsia represent carbon, iron, potassium, nitrogen, boron, oxygen, phosphorus, and arsenic, respectively.

Sodium dihydrophosphanide (NaPH_2_) [55] reacts cleanly with PhB(AdIm)_3_FeCl in a salt elimination reaction to afford the four–coordinate high spin (*S* = 2) complex PhB(AdIm)_3_FePH_2_ (**1**) in near quantitative yield (Scheme 1). The identity of the bright yellow compound has been authenticated by single crystal x‐ray diffraction and subsequent spectroscopic characterization admits it to the company of other phosphanide complexes (Figure 2) [56]. The x‐ray crystal structure of **1** reveals a pseudotetrahedral iron center (τ_4_ = 0.76) [57] that is coordinated by the tris(carbene)borate and the PH_2_^–^ ligand, with the latter lying on the molecular three–fold axis (∠B ···Fe ···P 173.4(4)°). The most salient structural feature is the Fe─P distance (2.444(7) Å) which is longer than that for diamagnetic *trans–*[Fe(dmpe)_2_(H)(PH_2_)] (Fe─P (2.337(1) Å)), the only other iron complex known for the PH_2_^–^ ligand [58]. The ATR–IR spectrum of **1** exhibits two overlapping broad absorption bands (ν_HPH_ = 2265, 2259 cm^−1^) which we attribute to the symmetric and asymmetric stretches of the PH_2_^–^ ligand. Similar ν_HPH_ stretching frequencies were observed for [Zr(TrenDMBS)(PH_2_)], [Th(TrenTIPS)(PH_2_)], and [U(TrenTIPS)(PH_2_)] [1, 37, 38]. The complex has also been characterized by ^1^H NMR spectroscopy and solution phase magnetometry (µ_eff_ = 4.9(2) µ_B_), indicating that *S* = 2 configuration of the precursor complex is conserved in **1**.

Similarly, treating PhB(AdIm)_3_FeCl with KAsH_2_ [59] provides the analogous arsanide complex PhB(AdIm)_3_FeAsH_2_ (**2**) complex as a golden yellow solid in near quantitative yield (Scheme 1). This complex has also been structurally authenticated by single crystal x‐ray diffraction, with a molecular structure that is isostructural to that of **1** (Figure 2). Interestingly, the Fe─As bond length (2.492(3) Å) is only slightly longer than the Fe─P bond length **1** despite the difference in covalent radii for these atoms (P 1.07 Å, As 1.19 Å). Nonetheless, this distance is in agreement with that predicted based on the covalent radii of iron and arsenic (ca. 2.54 Å) [60, 61]. To our knowledge, only one other structurally authenticated transition metal arsanide complex is known, namely Ir(CO)(Cl)H(PEt_3_)_2_(AsH_2_) [62].

The presence of the AsH_2_^–^ ligand is supported by the solid‐state vibrational spectrum (ATR–IR), with bands (ν_HAsH_ = 2068 cm^−1^, shoulder at 2053 cm^−1^) that are expectedly red–shifted from the ν_HPH_ bands in **1**. The energy of these bands compares favorably with that reported for (^Dipp^NacNac)ZnAsH_2_ (ν_HAsH_ = 2070 cm^−1^) [62], which further corroborates our spectroscopic assignment. The complex has also been characterized by ^1^H NMR spectroscopy and Evans’ method magnetometry (µ_eff_ = 4.8(3) µ_B_). Complex **2** is a rare example of a terminal parent arsanide ligand having no additional stabilization (e.g., as a pnictogenylborane) [63].

### 2.2 Deprotonation Affords Parent Pnictogenidene Ligands

**FIGURE 3:**
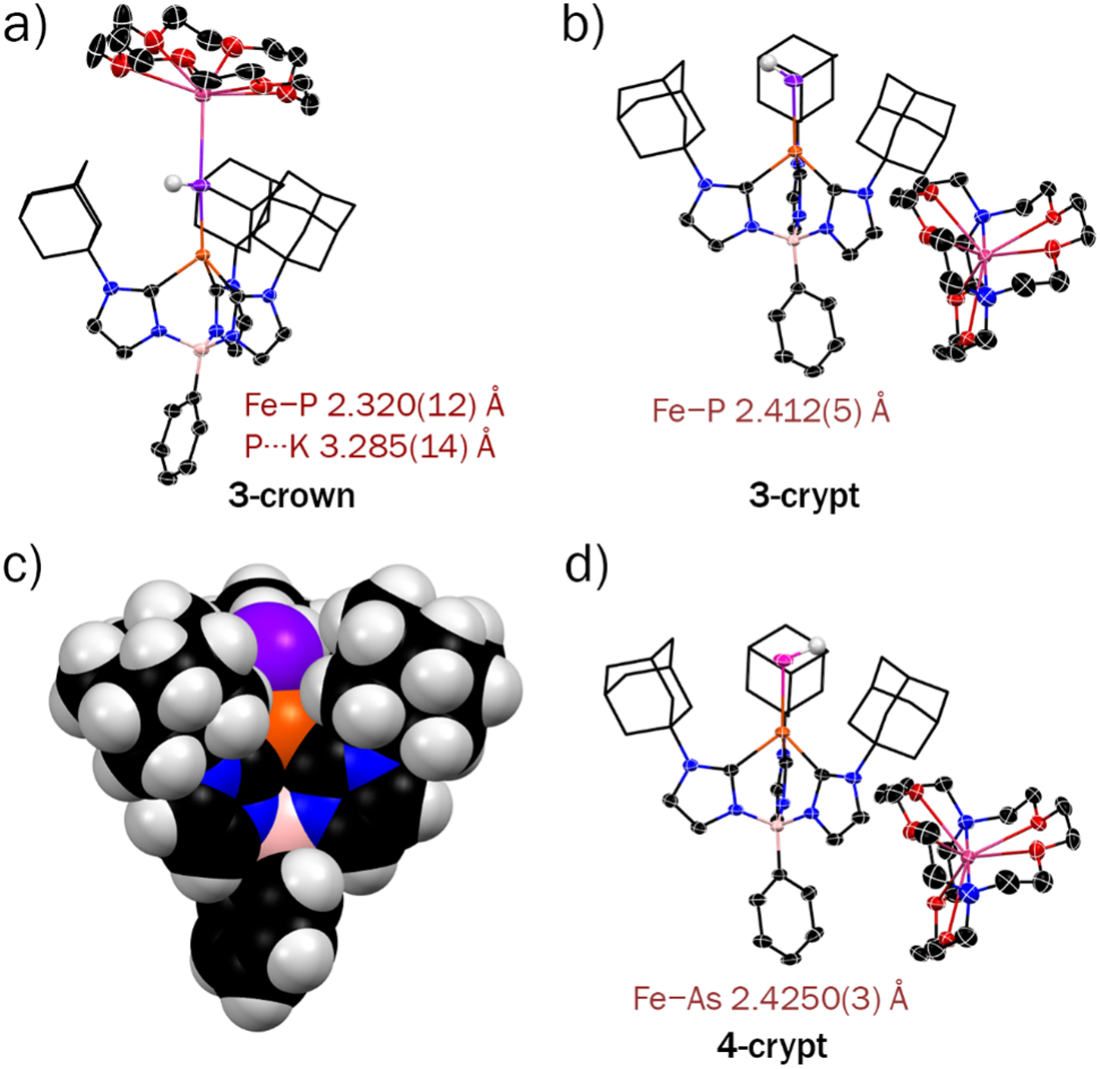
Molecular structures of phosphinidene and arsinidene complexes from single crystal x‐ray diffraction. ORTEP representations of phosphinidene complexes (a) **3‐crown** and (b) **3‐crypt**; (c) space–filling representation **3‐crypt** and (d) ORTEP representation of **4‐crypt**. Thermal ellipsoids are shown at 50%, probability, most hydrogen atoms omitted and most of the tris(carbene)borate ligand shown as wireframe for clarity. Bright pink, black, orange, magenta, blue, pink, red, and purple represent arsenic, carbon, iron, potassium, nitrogen, boron, oxygen, and phosphorus, respectively.

Remarkably, complex **1** reacts with excess benzyl potassium (KCH_2_Ph) in the presence of one molar equivalent 18‐crown‐6 to afford the parent phosphinidene complex [K(18‐crown‐6)][PhB(AdIm)_3_FePH] (**3‐crown**), which is isolated as dark red crystals after workup (Scheme 1). The solid‐state molecular structure of **3‐crown** has been elucidated by single crystal x‐ray diffraction as the closely interacting ion pair [K(18‐crown‐6)][PhB(AdIm)_3_FePH], see Figure 3a. The pseudotetrahedral iron center is coordinated by the tris(carbene)borate and phosphinidene ligands, the latter lying on molecular three–fold axis (∠B ···Fe ···P 177.0(9)°). The Fe─P bond distance in **3‐crown** (2.320(12) Å) is contracted by over 0.1 Å from that in **1**, suggesting iron–phosphorus multiple bond character. However, this distance is significantly longer than that computed for the hypothetical complex (OC)_4_Fe═PH (2.192 Å), which is suggested to have double bond character [32]. Remarkably, the Fe─P distance is longer than in most structurally characterized transition metal phosphinidenes, including complexes for 4d (η^6^–C_6_H_6_)Ru═PMes*, Mes* = 2,4,6‐^t^Bu_3_C_6_H_2_)) [64] and 5d (e.g., Cp*Ir(CO)═PMes*) [11] elements. The distance between the phosphorus atom and the potassium ion (3.285(14) Å) lies within the sum of their van der Waals radii. The potassium ion lies off the molecular threefold axis (∠Fe–P–K 153.23(6)°), suggesting a Lewis acid/base interaction involving a nucleophilic phosphinidene ligand.

The Fe─C distances (2.134(4)–2.104(4) Å) are typical for a high spin (*S* = 2) iron (II) tris(carbene)borate complex, suggesting that the oxidation and spin state of **1** are conserved in **3‐crown** [42, 65]. This is supported by zero–field ^57^Fe Mössbauer spectroscopy, where the isomer shift (δ = 0.67 mm s^−1^) and quadrupole splitting (Δ*E*_Q_ = 1.95 mm s^−1^) at 80 K are both in the range observed for other high spin iron(II) tris(carbene)borate complexes [43]. The presence of the phosphinidene ligand is corroborated by AT–IR, where a single broad band is observed (ν_PH_ = 2215 cm^−1^) at a frequency that greater than observed for [K(benzo‐15‐crown‐5)_2_][Zr(TrenDMBS)(PH)] (ν_PH_ = 2100 cm^−1^) [1] and (silox)_3_TaPH (ν_PH_ = 2150 cm^−1^) [35]. An intense band at 500 nm in the UV‐vis spectrum is assigned as a phosphinidene to iron LMCT band on the basis of TD‐DFT.

The ^1^H NMR spectrum of **3‐crown** exhibits six paramagnetically shifted resonances that can be assigned to the supporting tris(carbene)borate ligand of a three–fold symmetric species. We do not observe resonances that can be attributed to the phosphinidene ligand in the ^1^H or ^31^P{^1^H} NMR spectra. In addition, a paramagnetically broadened resonance at δ = 9.18 ppm is attributed to the protons of 18‐crown‐6, indicating that the tight ion pair observed in the solid‐state is maintained in solution. Interestingly, the ^1^H NMR spectrum of crystalline **3‐crown** in THF–d_8_ evolves over the course of a few hours, with the resonance for the crown becoming sharper and shifting toward higher frequency. We attribute this behavior to solvation of the cation by THF, which breaks the interaction with the phosphinidene,. suggesting that additional Lewis acid stabilization of this ligand is not needed. Indeed, we have been able to structurally characterize the ion‐separated complex, where the potassium ion is coordinated by two additional THF ligands (Figure S54).

To avoid the formation of tight ion pairs, we repeated the reaction of **1** with benzyl potassium in the presence of 2,2,2‐cryptand, which afforded dark red blocks of [K(2,2,2‐crypt)][PhB(AdIm)_3_FePH] (**3‐crypt**). In this case, the solid‐state structure reveals that phosphorous and potassium are well separated (9.762(6) Å), establishing that the phosphinidene ligand does not require additional Lewis acid stabilization (Figure 3b). A space filling model reveals that the tris(carbene)borate ligand provides a protective pocket around the terminal phosphinidene ligands (Figure 3c). Interestingly, the solid‐state structure of **3‐crypt** reveals the terminal phosphinidene ligand has a longer Fe─P bond length (2.412(5) Å) than in **3‐crown**, although the Fe─C distances (2.027(14)–2.063(14) Å) are shorter. While it is tempting to ascribe these changes to differences in the electron density of the phosphinidene ligand in the presence and absence of the Lewis acidic cation, a linear synchronous transit (LST) calculation reveals that the potential energy surface associated with the Fe─P bond distance is relatively shallow (Figure S40). These structural differences are likely the result of packing forces in the solid‐state. Complexes **3‐crown** and **3‐crypt** have distinct ^1^H NMR spectra, suggesting that counter cation coordination impacts the electronic structure of the complex, which is supported by a shift in the LMCT band to 567 nm. However, this does not affect the strength of the P─H bond since ν_PH_ is the same for both complexes.

Arsanide complex **2** also reacts with excess benzyl potassium in the presence of either one molar equivalent 18‐crown‐6 or 2,2,2‐cryptand to afford the deep violet arsinidene complexes [K(18‐crown‐6)][PhB(AdIm)_3_FeAsH] (**4‐crown**) and [K(2,2,2‐crypt][PhB(AdIm)_3_FeAsH] (**4‐crypt**), see Scheme 1. The molecular structure of **4‐crypt** has been determined by x‐ray diffraction (Figure 3d). The Fe─As bond length in **4‐crypt** (2.4250(3) Å) is within that predicted from the sum of their covalent radii (2.47 Å) [60, 61]. The arsinidene ligand lies on molecular three–fold axis (∠B ···Fe ···As 177.2(4)°) with the Fe─C distances (2.167(2)–2.110(2) Å) typical for high spin (*S* = 2) iron(II) tris(carbene)borate complexes. While low crystal quality prevents discussion of metrical data, we have been able to determine the connectivity of [K(18‐crown‐6)][PhB(AdIm)_3_FeAsH] (**4‐crown**) by x‐ray crystallography. Here, it is notable that the potassium ion is fully sequestered away from the arsinidene ligand, in contrast to the structure of **3‐crown**, indicating the arsinidene is less nucleophilic than the phosphinidene ligand.

To the best of our knowledge, the only other structurally characterized transition metal complex with a terminal parent arsinidene is (PN)_2_Ti═AsH [39]. It is notable that the Ti═As bond length in this complex (2.3775(4) Å) is shorter than the Fe─As distance in **4‐crypt**, despite the larger size of titanium (covalent radii: Ti 1.60 Å; Fe 1.52 Å) [60]. This suggests that **4‐crypt** has a lower Fe─As bond order than (PN)_2_Ti═AsH, which is corroborated by electronic structure calculations (see below). The Fe─As bond in **4‐crypt** is also longer than in [{(NHC)C(Ph)}As]Fe(CO)_4_ complexes (2.367(4)–2.375(4) Å), which feature Lewis base–stabilized arsinidene ligands [66].

Complexes **4‐crown** and **4‐crypt** have also been spectroscopically characterized. As with **3‐crown**, the ^57^Fe Mössbauer spectral parameters of **4‐crypt** at 80 K (δ = 0.61 mm s^−1^, Δ*E*_Q_ = 1.76 mm s^−1^) are similar to those observed for other high spin iron(II) tris(carbene)borate complexes. The AsH^2–^ ligand has been spectroscopically characterized by the AT–IR spectra of both **4‐crown** and **4‐crypt** by a broad band at ∼1958 cm^−1^, which is similar to that observed in (silox)_3_Ta═AsH (ν_AsH_ = 1950 cm^−1^) [35], but at lower frequency than in (PN)_2_Ti═AsH (ν_AsH_ = 2002 cm^−1^) [39]. The complexes have also been characterized by ^1^H NMR and UV‐vis spectroscopy.

### 2.3 Electronic Structure

The paramagnetic metal ion in complexes **3‐crown/crypt** and **4‐crown/crypt** distinguishes these complexes from other parent phosphinidene and arsinidene complexes, which have closed shell configurations. To better understand the bonding in these unique complexes, we turned to electronic structure calculations of the anionic iron complexes using DFT (B3LYP/ma‐def2‐TZVP), as implemented in Orca [67]. These calculations are benchmarked to the experimentally observed ^57^Fe Mössbauer parameters.

**FIGURE 4:**
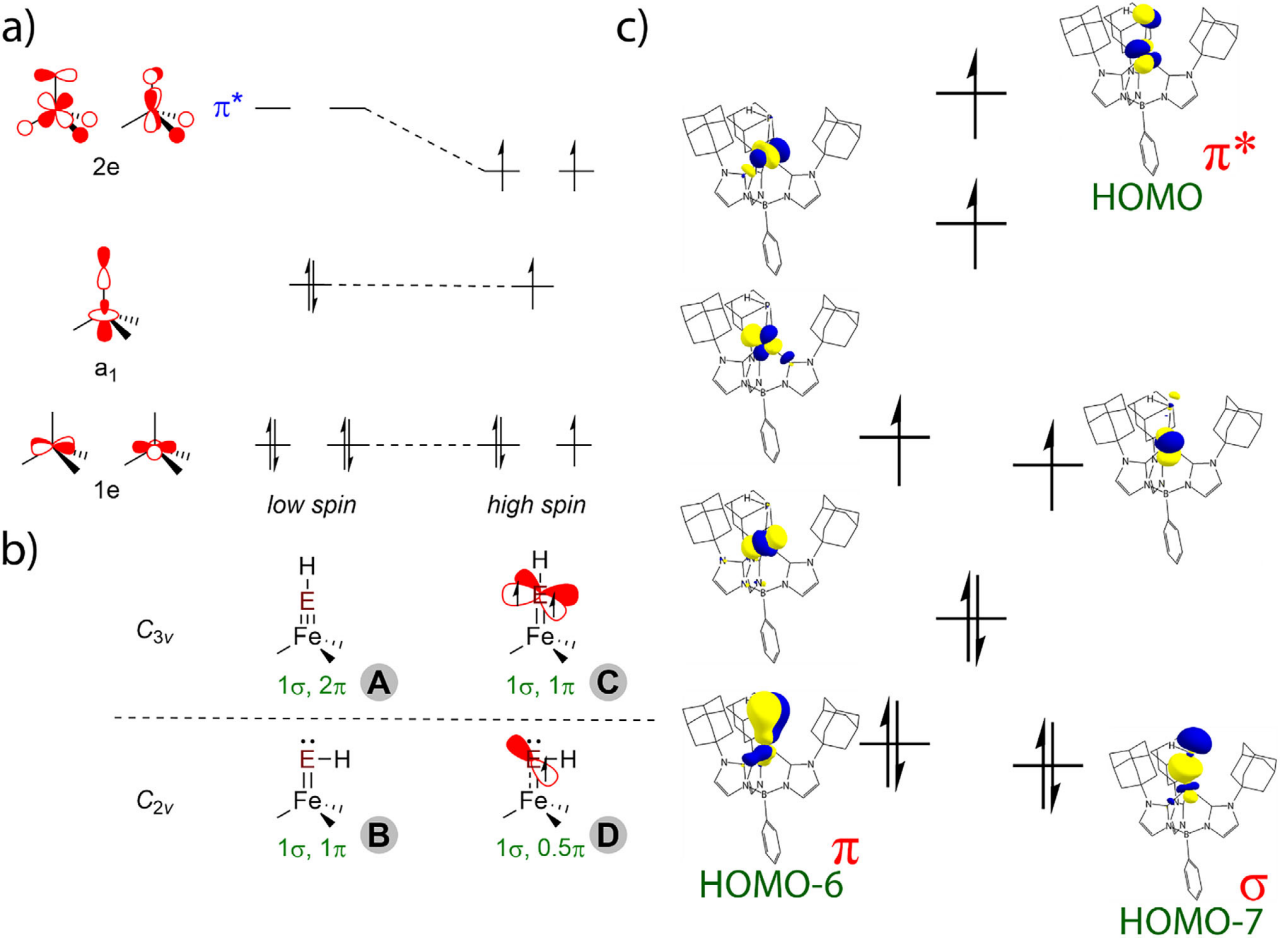
(a) Qualitative MO diagram for low (*S* = 0) and high (*S* = 2) spin four‐coordinate iron(II) tris(carbene)borate complexes in three‐fold symmetry; (b) bonding representations for terminal pnictogenide complexes with linear (*C*_3_ *_v_*) and bent (*C*_2_ *_v_*) Fe–E–H ligands; (c) natural orbital representation of the frontier orbitals for high spin [Ph(AdIm)_3_Fe═PH]^–^, as determined by DFT (B3LYP/ma‐def2‐TZVP), isodensity at 0.05.

To better understand the impact of spin state on bonding, we first consider a qualitative MO diagram for a four‐coordinate tris(carbene)borate complex in *C*_3_ *_v_* symmetry, where the iron d orbitals transform as 1a_1_ + 2e (Figure 4a). Ignoring additional e‐e mixing, the lower energy a_1_ and 1e sets are largely nonbonding, while the 2e orbitals are π* with respect to the axial ligand. Four coordinate iron(II) tris(carbene)borate complexes have been observed in both low spin (*S* = 0) and high spin (*S* = 2) states [68, 69]. In the case of low spin iron(II), the pnictogenidene ligand [EH]^2–^ is expected to be linear with an Fe≡E triple bond (Figure 4b, structure A). A nonlinear pnictogenidene ligand, which lowers the symmetry of the complex to *C*_2_ *_v_*, is expected to have an Fe═E double bond along with a pnictogen lone pair. This ligand structure has been observed for other pnictogenidene complexes (Figure 4b, structure B). For high spin iron(II), the singly occupied 2e orbitals decrease the extent of π‐bonding, resulting in an Fe═E double bond along with unpaired electron density in the perpendicular pnitogen p orbitals (Figure 4b, structure C). As with the low spin state, the lone pair character on the pnictogen atom in a nonlinear high spin pnictogenidene complex comes at the expense of π‐bonding to iron (Figure 4b, structure D).

Electronic structure calculations for hypothetical *S* = 0 [PhB(AdIm)_3_FePH]^–^ are consistent with structure A. Geometry optimization provides a very short Fe─P bond distance (2.071 Å) with a linear phosphinidene ligand (Fe─P─H 179.2°). The Fe≡P triple bond results from fully occupied Fe─P σ and π orbitals, see Figure S41. On the other hand, the structure of *S* = 0 [PhB(AdIm)_3_FeAsH]^–^ is consistent with B. Here, the optimized structure reveals a bent arsenide ligand (Fe─As─H 141.3°) with an Fe─As bond (2.211 Å) that is longer than expected based on differences in the covalent radii of phosphorus and arsenic. The Fe═As double bond arises from one σ and one π bond, with a lone pair on the As atom (Figure S42). The structural differences between the two pnictogenidene ligands are attributed to decreased π overlap as the principle quantum number increases.

In contrast to the low spin state, the computed electronic structures for both pnictogenidene complexes are consistent with structure D. As illustrated for [PhB(AdIm)_3_FePH]^–^, the doubly occupied Fe─P σ (HOMO–7) and π (HOMO–6) orbitals are partially offset by the Fe─P π* character of the singly occupied HOMO (Figure 4). All other iron d–orbitals are non–bonding with respect to the iron–phosphorus bond, leading to an iron‐phosphorus bond order of 1.5. The majority of the spin density is located on iron (Löwdin spin density 3.42) with a non–negligible amount on phosphorus (Löwdin spin density 0.31) which is a consequence of the Fe─P π* character of the SOMO which transfers unpaired electron density to the phosphorus atom. Likewise, the computed electronic structure for [PhB(AdIm)_3_FeAsH]^–^ reveals an iron‐arsenic bond order of 1.5 that arises from doubly occupied Fe─As σ and π orbitals that are partially offset by the Fe─As π* character of the singly occupied HOMO (Figure S43).

**FIGURE 5:**
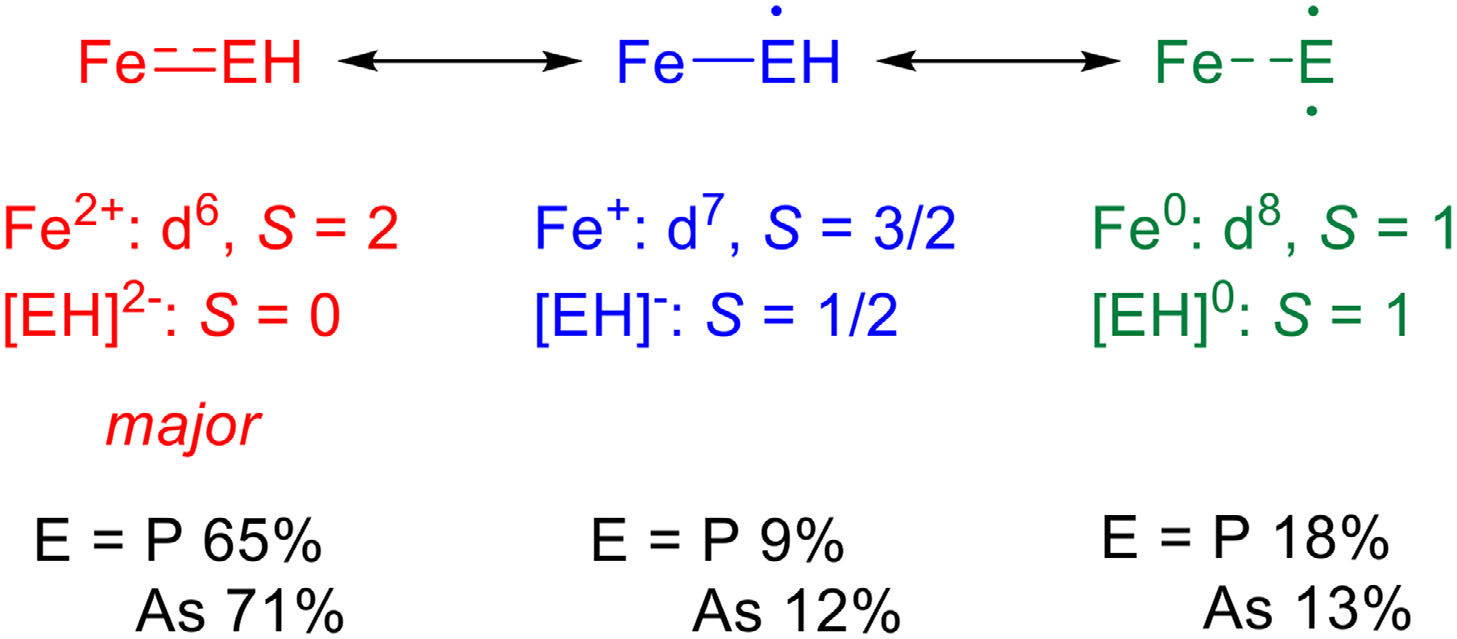
Resonance structure description of the bonding in [H(MeIm)_3_Fe = EH]^–^ (E = P, As), as determined from CASSCF(12,8) calculations without state averaging.

Additional insight into the electronic structure comes from multireference complete active space self–consistent field (CASSCF) calculations for the truncated complexes [HB(MeIm)_3_FeEH]^–^ (E = P, As) where the adamantyl groups were replaced by methyls and the phenyl group by a proton. The pnictogenidene complexes have similar electronic structures. An active space containing the iron 3d orbitals and the phosphorus 3p orbitals (12 electrons, 8 orbitals) reveals that the dominant configuration corresponds to *S* = 2 Fe^II^ bound to a dianionic pnictogenidene (EH^2–^) ligand (Figure 5) [70]. This configuration is the same as that determined by the single determinant DFT calculations. A second configuration involves high spin Fe^0^ (*S* = 1) ferromagnetically coupled to a neutral pnictogenidene ligand in its triplet state. The pnictogenidene electrons are no longer involved π bonding. A third configuration corresponds to high spin Fe^I^ (*S* = 3/2) ferromagnetically coupled to a pnitogenidenyl radical anion (EH^–^). The unpaired electron on the pnitogenidenyl is not involved in π bonding, leading to a bond order of 1.

### 2.4 Reactivity

**SCHEME 2:**
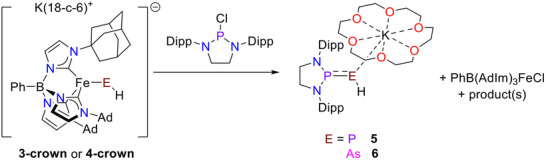
Nucleophilic reactivity of phosphinidene and arsinidene complexes **3‐crown** and **4‐crown**.

Initial reactivity studies reveal the phosphinidene and arsinidene complexes to be nucleophilic at the pnictogen atom, as suggested by the crystal structure of **3‐crown**. For example, **3‐crown** reacts instantaneously with pyridinium hydrochloride in THF to provide **1**, although a number of unidentified products are also formed. Similarly, chlorodiazaphospholidine (DippNCH_2_)_2_PCl reacts with **3‐crown** to provide [K(18‐crown‐6)][(DippNCH_2_)_2_PPH], previously reported as the potassium salt [71], (**5**) as characterized by ^31^P{^1^H}, ^31^P and ^1^H NMR spectroscopy (Scheme 2, Figures S14 and S15). The same reaction with **4‐crown** provides the phosphino–arsinidene [K(18‐crown–6)][(DippNCH_2_)_2_PAsH] (**6**), once thought to exist only transiently (Scheme 2) [72]. Indeed, while this compound is on the cusp of stability, we are able to isolate single crystals that allow us to confirm its connectivity by single crystal x‐ray diffraction (Figure S58). While the arsinidene hydrogen was located in the Fourier difference electron density map, the quality of the data prevents discussion of metrical data. Multinuclear NMR spectroscopy confirms the P─As─H linkage in **6** (Figures S16–S20). Notably, a doublet in the ^31^P NMR spectrum (δ = 204 ppm, ^2^ *J*_P–H_  =  27 Hz) has a similar chemical shift and P─H coupling constant to that observed for the phospholidine phosphorus atom in **5** (δ = 182 ppm, ^1^ *J*_P–P_  =  429 Hz, ^2^ *J*_P–H_  =  38 Hz). In addition, the chemical shift of this proton in the ^1^H NMR spectrum (δ = –0.25 ppm, ^2^J_P–H_  =  27 Hz) lies within the range observed for analogous examples (δ = –2.22 to 2.26 ppm) [73, 74], in line with the As─H assignment. The As─H bond was also characterized by IR spectroscopy (ν_AsH_ = 1958 cm^−1^).

Reactions of **3‐crown** and **4‐crown** with small molecules (e.g., H_2_, CO, and CO_2_) and electrophiles (e.g., Me_3_SiCl) resulted in multiple unidentified products. In some cases, there is evidence for competitive electron transfer reactions. For example, bibenzyl was identified from the reaction of **3‐crown** with benzyl bromide, among other unidentified products.

## 3 Conclusion

The pnictogenidene complexes in this paper adopt multiconfigurationial electronic structures, all of which have attenuated metal–ligand multiple bond character. The dominant configuration can be considered as being Schrock‐like in which multiple bonding to a dianionic ligand is diminished by the partially filled Fe–E π* orbital. A second configuration is Fischer‐like with a neutral pnictogenidene ligand bound to low valent iron. Since the iron center is high spin, there are no vacant orbitals to stabilize the ligand in its singlet form, which also decreases the bond order. The final configuration is similar to the previously proposed biradical resonance for Cp_2_Ti(PMe_3_)(=E^R^ter) [30] except that the coupling between unpaired electrons on iron and the pnictogenidene ligand is ferromagnetic, which prevents the formation of π bond. The pnictogenidene ligand is expected to be nucleophilic for all these configurations, although the negative charge on the complex is also important to the observed reactivity.

In summary, we have demonstrated access to structurally authenticated late metal phosphinidene and arsinidene complexes that are notable for the lack of stabilizing groups on the pnictogenide ligands. The high spin state of these complexes leads to a multiconfigurational electronic structure that attenuates the metal–ligand multiple bond character normally associated with pnictogenidene ligands. This work also provides a basis for extending the library of late metal–pnictogenidene linkages to other pnictogens.

## Conflicts of Interest

The authors declare no conflicts of interest.

## Supporting information




**Supporting File 1**: Additional experimental details, characterization data (NMR, ^57^Fe Mössbauer, IR, UV–vis, mass spectrometry) and computational details (pdf). DFT optimized coordinates (xyz). The authors have cited additional references within the Supporting Information [75–88].

**Supporting File 2**: anie71880‐sup‐0002‐Data.zip

## Data Availability

The data that support the findings of this study are available in the Supporting Information of this article.

## References

[1] H. Stafford , T. M. Rookes , E. P. Wildman , et al., “Terminal Parent Phosphanide and Phosphinidene Complexes of Zirconium(IV),” Angewandte Chemie International Edition 56 (2017): 7669–7673, 10.1002/anie.201703870. 28489308 PMC5575506

[2] D. W. Stephan , “Zirconium—Phosphorus Chemistry: Strategies in Syntheses, Reactivity, Catalysis, and Utility,” Angewandte Chemie International Edition 39 (2000): 314–329, 10.1002/(SICI)1521-3773(20000117)39:2<314::AID-ANIE314>3.0.CO;2-D. 10649398

[3] K. Lammertsma , New Aspects in Phosphorus Chemistry III, Vol. 229, Ed. J.‐P. Majoral (Springer Berlin Heidelberg, 2003) 95–119.

[4] H. Aktas , J. C. Slootweg , and K. Lammertsma , “Nucleophilic Phosphinidene Complexes: Access and Applicability,” Angewandte Chemie (International ed in English) 49 (2010): 2102–2113, 10.1002/anie.200905689. 20157897

[5] P. B. Hitchcock , M. F. Lappert , and W.‐P. Leung , “The First Stable Transition Metal (Molybdenum or Tungsten) Complexes Having a Metal–Phosphorus( III) Double Bond: The Phosphorus Analogues of Metal Aryl‐ and Alkyl‐Imides; *X*‐ray Structure of [Mo(η‐C_5_H_5_)_2_ (═PAr)](Ar = C_6_H_2_Bu^t^ _3_‐2,4,6),” Journal of the Chemical Society, Chemical Communications (1987): 1282–1283, 10.1039/C39870001282.

[6] Z. Hou , T. L. Breen , and D. W. Stephan , “Formation and Reactivity of the Early Metal Phosphides and Phosphinidenes Cp*2Zr:PR, Cp*2Zr(PR)2, and Cp*2Zr(PR)3,” Organometallics 12 (1992): 3158–3167, 10.1021/om00032a044.

[7] C. C. Cummins , R. R. Schrock , and W. M. Davis , “Phosphinidenetantalum(V) Complexes of the Type [(N_3_N)Ta═PR] as Phospha‐Wittig Reagents,” Angewandte Chemie (International ed in English) 32 (1993): 756–759, 10.1002/anie.199307561.

[8] J. B. Bonanno , P. T. Wolczanski , and E. B. Lobkovsky , “Arsinidene, Phosphinidene, and Imide Formation via 1,2‐H2‐Elimination From (silox)3HTaEHPh (E = N, P, As): Structures of (silox)3Ta:EPh (E = P, As),” Journal of the American Chemical Society 116 (1994): 11159–11160, 10.1021/ja00103a042.

[9] J. S. Freundlich , R. R. Schrock , and W. M. Davis , “Synthetic and Mechanistic Investigations of Trimethylsilyl‐Substituted Triamidoamine Complexes of Tantalum That Contain Metal−Ligand Multiple Bonds,” Journal of the American Chemical Society 118 (1996): 3643–3655, 10.1021/ja953826n.

[10] R. Melenkivitz , D. J. Mindiola , and G. L. Hillhouse , “Monomeric Phosphido and Phosphinidene Complexes of Nickel,” Journal of the American Chemical Society 124 (2002): 3846–3847, 10.1021/ja017787t. 11942818

[11] A. T. Termaten , T. Nijbacker , M. Schakel , M. Lutz , A. L. Spek , and K. Lammertsma , “Synthesis of Novel Terminal Iridium Phosphinidene Complexes,” Organometallics 21 (2002): 3196–3202, 10.1021/om020062t.

[12] F. Basuli , J. Tomaszewski , J. C. Huffman , and D. J. Mindiola , “Four‐Coordinate Phosphinidene Complexes of Titanium Prepared by α‐H‐Migration: Phospha‐Staudinger and Phosphaalkene‐Insertion Reactions,” Journal of the American Chemical Society 125 (2003): 10170–10171, 10.1021/ja036559r. 12926929

[13] R. Menye‐Biyogo , F. Delpech , A. Castel , H. Gornitzka , and P. Riviere , “Ruthenium‐Stabilized Low‐Coordinate Phosphorus Atoms: Structural Evidence for Monomeric Metaphosphonate,” Angewandte Chemie (International ed in English) 42 (2003): 5610–5612, 10.1002/anie.200352306. 14639729

[14] A. T. Termaten , H. Aktas , M. Schakel , et al., “Terminal Phosphinidene Complexes Cp^R^(L)═MPAr of the Group 9 Transition Metals Cobalt, Rhodium, and Iridium. Synthesis, Structures, and Properties,” Organometallics 22 (2003): 1827–1834, 10.1021/om0208624.

[15] A. T. Termaten , T. Nijbacker , M. Schakel , M. Lutz , A. L. Spek , and K. Lammertsma , “Synthesis and Reactions of Terminal Osmium and Ruthenium Complexed Phosphinidenes [(*η* ^6^‐Ar)(L)M═PMes*],” Chemistry–A European Journal 9 (2003): 2200–2208, 10.1002/chem.200204582. 12772294

[16] A. T. Termaten , M. Schakel , A. W. Ehlers , M. Lutz , A. L. Spek , and K. Lammertsma , “N‐Heterocyclic Carbene Functionalized Iridium Phosphinidene Complex [Cp*(NHC)Ir═PMes*]: Comparison of Phosphinidene, Imido, and Carbene Complexes,” Chemistry–A European Journal 9 (2003): 3577–3582, 10.1002/chem.200304744. 12898684

[17] F. Basuli , B. C. Bailey , J. C. Huffman , M.‐H. Baik , and D. J. Mindiola , “Terminal and Four‐Coordinate Vanadium(IV) Phosphinidene Complexes. A Pseudo Jahn−Teller Effect of Second Order Stabilizing the V─P Multiple Bond,” Journal of the American Chemical Society 126 (2004): 1924–1925, 10.1021/ja0392216. 14971911

[18] B. C. Bailey , J. C. Huffman , D. J. Mindiola , W. Weng , and O. V. Ozerov , “Remarkably Stable Titanium Complexes Containing Terminal Alkylidene, Phosphinidene, and Imide Functionalities,” Organometallics 24 (2005): 1390–1393, 10.1021/om0490583.

[19] G. Zhao , F. Basuli , U. J. Kilgore , et al., “Neutral and Zwitterionic Low‐Coordinate Titanium Complexes Bearing the Terminal Phosphinidene Functionality. Structural, Spectroscopic, Theoretical, and Catalytic Studies Addressing the Ti─P Multiple Bond,” Journal of the American Chemical Society 128 (2006): 13575–13585, 10.1021/ja064853o. 17031972

[20] H. Aktas , J. C. Slootweg , A. W. Ehlers , M. Lutz , A. L. Spek , and K. Lammertsma , “N‐Heterocyclic Carbene Functionalized Group 7−9 Transition Metal Phosphinidene Complexes,” Organometallics 28 (2009): 5166–5172, 10.1021/om900496y.

[21] H. Aktas , J. C. Slootweg , M. Schakel , et al., “N‐Heterocyclic Carbene‐Functionalized Ruthenium Phosphinidenes: What a Difference a Twist Makes,” Journal of the American Chemical Society 131 (2009): 6666–6667, 10.1021/ja901540h. 19397260

[22] U. J. Kilgore , H. Fan , M. Pink , E. Urnezius , J. D. Protasiewicz , and D. J. Mindiola , “Phosphinidene Group‐Transfer With a Phospha‐Wittig Reagent: A New Entry to Transition Metal Phosphorus Multiple Bonds,” Chemical Communications (2009): 4521–4523, 10.1039/b910410k. 19617970

[23] V. M. Iluc and G. L. Hillhouse , “Hydrogen‐Atom Abstraction From Ni(I) Phosphido and Amido Complexes Gives Phosphinidene and Imide Ligands,” Journal of the American Chemical Society 132 (2010): 15148–15150, 10.1021/ja107115q. 20929225

[24] R. Waterman and T. D. Tilley , “Terminal Hafnium Phosphinidene Complexes and Phosphinidene Ligand Exchange,” Chemical Science 2 (2011): 1320–1325, 10.1039/C1SC00119A.

[25] M. A. Rankin and C. C. Cummins , “Terminal Phosphinidene Formation via Tantalaziridine Complexes,” Dalton Transactions 41 (2012): 9615, 10.1039/c2dt31082a. 22790093

[26] A. Grundmann , M. B. Sárosi , P. Lönnecke , R. Frank , and E. Hey‐Hawkins , “Terminal Alkylphosphanylidene Organo­Tantalum(V) Complexes,” European Journal of Inorganic Chemistry 2013 (2013): 3137–3140, 10.1002/ejic.201300500.

[27] K. Searles , P. J. Carroll , and D. J. Mindiola , “Anionic and Mononuclear Phosphinidene and Imide Complexes of Niobium,” Organometallics 34 (2015): 4641–4643, 10.1021/acs.organomet.5b00518.

[28] J. Abbenseth , D. Delony , M. C. Neben , C. Wurtele , B. de Bruin , and S. Schneider , “Interconversion of Phosphinyl Radical and Phosphinidene Complexes by Proton Coupled Electron Transfer,” Angewandte Chemie (International ed in English) 58 (2019): 6338–6341, 10.1002/anie.201901470. 30840783 PMC6519162

[29] A. Doddi , D. Bockfeld , T. Bannenberg , and M. Tamm , “N‐Heterocyclic Carbene Analogues of Nucleophilic Phosphinidene Transition Metal Complexes,” Chemistry–A European Journal 26 (2020): 14878–14887, 10.1002/chem.202003099. 32721063 PMC7756676

[30] M. Fischer , F. Reiß , and C. Hering‐Junghans , “Titanocene Pnictinidene Complexes,” Chemical Communications 57 (2021): 5626–5629, 10.1039/D1CC01305J. 33989372

[31] T. E. Rieser , P. Wetzel , C. Maichle‐Mossmer , P. Sirsch , and R. Anwander , “A Terminal Yttrium Phosphinidene,” Journal of the American Chemical Society 145 (2023): 17720–17733, 10.1021/jacs.3c04335. 37531590

[32] A. W. Ehlers , E. J. Baerends , and K. Lammertsma , “Nucleophilic or Electrophilic Phosphinidene Complexes ML n PH; What Makes the Difference?,” Journal of the American Chemical Society 124 (2002): 2831–2838, 10.1021/ja017445n. 11890835

[33] A. W. Ehlers , K. Lammertsma , and E. J. Baerends , “Phosphinidene Complexes M(CO) 5−PR: A Density Functional Study on Structures and Electronic States,” Organometallics 17 (1998): 2738–2742, 10.1021/om980057i.

[34] J. B. M. Wit , G. T. van Eijkel , M. Schakel , and K. Lammertsma , “The Electrophilic Phosphinidene Complex * ^i^ *Pr_2_N–P Fe(CO)_4_ Trapped by Alkynes,” Tetrahedron 56 (2000): 137–141, 10.1016/S0040-4020(99)00782-6.

[35] E. B. Hulley , J. B. Bonanno , P. T. Wolczanski , T. R. Cundari , and E. B. Lobkovsky , “Pnictogen‐Hydride Activation by (silox)_3_Ta (silox = * ^t^ *Bu_3_SiO); Attempts to Circumvent the Constraints of Orbital Symmetry in N_2_ Activation,” Inorganic Chemistry 49 (2010): 8524–8544, 10.1021/ic101147x. 20722448

[36] K. F. Hirsekorn , A. S. Veige , and P. T. Wolczanski , “PC Bond Cleavage of (silox)_3_ NbPMe_3_ (silox = * ^t^ *Bu_3_SiO) Under Dihydrogen Leads to (silox)_3_ Nb═CH_2_, (silox)_3_Nb═PH or (silox)_3_ NbP(H)Nb(silox)_3_, and CH_4_ ,” Journal of the American Chemical Society 128 (2006): 2192–2193, 10.1021/ja057747f. 16478155

[37] B. M. Gardner , G. Balázs , M. Scheer , et al., “Triamidoamine–Uranium(IV)‐Stabilized Terminal Parent Phosphide and Phosphinidene Complexes,” Angewandte Chemie International Edition 53 (2014): 4484–4488, 10.1002/anie.201400798. 24644135

[38] E. P. Wildman , G. Balázs , A. J. Wooles , M. Scheer , and S. T. Liddle , “Thorium–Phosphorus Triamidoamine Complexes Containing Th–P Single‐ and Multiple‐Bond Interactions,” Nature Communications 7 (2016): 12884–12884, 10.1038/ncomms12884. PMC5056418 27682617

[39] M. Bhunia , J. S. Mohar , C. Sandoval‐Pauker , et al., “Softer Is Better for Titanium: Molecular Titanium Arsenido Anions Featuring Ti≡As Bonding and a Terminal Parent Arsinidene,” Journal of the American Chemical Society 146 (2024): 3609–3614, 10.1021/jacs.3c12939. 38290427

[40] M. K. Goetz , E. A. Hill , A. S. Filatov , and J. S. Anderson , “Isolation of a Terminal Co(III)‐Oxo Complex,” Journal of the American Chemical Society 140 (2018): 13176–13180, 10.1021/jacs.8b07399. 30078327

[41] M. Ding , G. E. Cutsail , D. Aravena , et al., “A Low Spin Manganese(iv) Nitride Single Molecule Nagnet,” Chemical Science 7 (2016): 6132–6140, 10.1039/C6SC01469K. 27746891 PMC5058364

[42] D. C. Wannipurage , E. S. Yang , A. D. Chivington , et al., “A Transient Iron Carbide Generated by Cyaphide Cleavage,” Journal of the American Chemical Society 146 (2024): 27173–27178, 10.1021/jacs.4c10704. 39287969

[43] J. A. Valdez‐Moreira , D. M. Beagan , H. Yang , et al., “Hydrocarbon Oxidation by an Exposed, Multiply Bonded Iron(III) Oxo Complex,” ACS Central Science 7 (2021): 1751–1755, 10.1021/acscentsci.1c00890. 34729418 PMC8554833

[44] J. A. Valdez‐Moreira , D. C. Wannipurage , M. Pink , et al., “Hydrogen Atom Abstraction by a High‐Spin [Fe^III^=S] Complex,” Chem 9 (2023): 2601–2609, 10.1016/j.chempr.2023.05.007. 39021493 PMC11251717

[45] J. J. Scepaniak , C. S. Vogel , M. M. Khusniyarov , F. W. Heinemann , K. Meyer , and J. M. Smith , “Synthesis, Structure, and Reactivity of an Iron(V) Nitride,” Science 331 (2011): 1049–1052, 10.1126/science.1198315. 21350172

[46] J. J. Scepaniak , J. A. Young , R. P. Bontchev , and J. M. Smith , “Formation of Ammonia From an Iron Nitrido Complex,” Angewandte Chemie International Edition 48 (2009): 3158–3160, 10.1002/anie.200900381. 19322861

[47] J. J. Scepaniak , M. D. Fulton , R. P. Bontchev , E. N. Duesler , M. L. Kirk , and J. M. Smith , “Structural and Spectroscopic Characterization of an Electrophilic Iron Nitrido Complex,” Journal of the American Chemical Society 130 (2008): 10515–10517, 10.1021/ja8027372. 18630913

[48] L. Bucinsky , M. Breza , W.‐T. Lee , et al., “Spectroscopic and Computational Studies of Spin States of Iron(IV) Nitrido and Imido Complexes,” Inorganic Chemistry 56 (2017): 4751–4768, 10.1021/acs.inorgchem.7b00512. 28379707

[49] I. Nieto , F. Ding , R. P. Bontchev , H. Wang , and J. M. Smith , “Thermodynamics of Hydrogen Atom Transfer to a High‐Valent Iron Imido Complex,” Journal of the American Chemical Society 130 (2008): 2716–2717, 10.1021/ja0776834. 18266366

[50] M. E. García , D. García‐Vivó , M. A. Ruiz , and D. Sáez , “Divergent Reactivity of a Phosphinidene‐Bridged Dimolybdenum Complex Toward 1‐Alkynes: P–C, P–H, C–C, and C–H Couplings,” Organometallics 36 (2017): 1756–1764, 10.1021/acs.organomet.7b00121.

[51] R. Waterman , “A “Bottle‐Able” Phosphinidene,” Chem 1 (2016): 27–29, 10.1016/j.chempr.2016.06.005.

[52] L. Liu , D. A. Ruiz , D. Munz , and G. Bertrand , “A Singlet Phosphinidene Stable at Room Temperature,” Chem 1 (2016): 147–153, 10.1016/j.chempr.2016.04.001.

[53] M. A. Alvarez , M. E. García , M. A. Ruiz , and J. Suárez , “Enhanced Nucleophilic Behavior of a Dimolybdenum Phosphinidene Complex: Multicomponent Reactions With Activated Alkenes and Alkynes in the Presence of CO or CNXyl,” Angewandte Chemie International Edition 50 (2011): 6383–6387, 10.1002/anie.201101940. 21604348

[54] E. Zars , M. R. Mena , M. R. Gau , and D. J. Mindiola , “Flash Communication: A Ferrous Adduct of a Phosphanylidene‐σ 4‐Phosphorane,” Organometallics 43 (2024): 1947–1951, 10.1021/acs.organomet.4c00329.

[55] D. Ergöçmen and J. M. Goicoechea , “Synthesis, Structure and Reactivity of a Cyapho‐Cyanamide Salt,” Angewandte Chemie International Edition 60 (2021): 25286–25289, 10.1002/anie.202111619. 34554622

[56] Deposition numbers 2487466, 2487474, 2487476, 2487477, 2487479, 2487484, 2487485 contain the supplementary crystallographic data for this paper. These data are provided free of charge by the joint Cambridge Crystallographic Data Centre and Fachinformationszentrum Karlsruhe Access Structures service.

[57] L. Yang , D. R. Powell , and R. P. Houser , “Structural Variation in Copper(i) Complexes With Pyridylmethylamide Ligands: Structural Analysis With a New Four‐Coordinate Geometry Index, *τ* _4_ ,” Dalton Transactions (2007): 955–964, 10.1039/B617136B. 17308676

[58] S. Lau , M. F. Mahon , and R. L. Webster , “Synthesis and Characterization of a Terminal Iron(II)–PH_2_ Complex and a Series of Iron(II)–PH_3_ Complexes,” Inorganic Chemistry 63 (2024): 6998–7006, 10.1021/acs.inorgchem.4c00605. 38563561 PMC11022175

[59] KAsH2 and [Na(18‐crown‐6)][AsH2] were synthesized by a new procedure, see Supporting Information for details.

[60] B. Cordero , V. Gómez , A. E. Platero‐Prats , et al., “Covalent Radii Revisited,” Dalton Transactions (2008): 2832, 10.1039/b801115j. 18478144

[61] P. Pyykkö , “Additive Covalent Radii for Single‐, Double‐, and Triple‐Bonded Molecules and Tetrahedrally Bonded Crystals: A Summary,” Journal of Physical Chemistry A 119 (2015): 2326–2337, 10.1021/jp5065819. 25162610

[62] K. Dollberg , J. Moritz , K. Mészáros , et al., “Synthesis and Characterization of Novel Transition Metal Pnictogenide Compounds,” Zeitschrift für anorganische und allgemeine Chemie 650 (2024): e202400023, 10.1002/zaac.202400023.

[63] F. Lehnfeld , O. Hegen , G. Balázs , A. Y. Timoshkin , and M. Scheer , “Coordination Chemistry of Pnictogenylboranes Towards Group 6 Transition Metal Lewis Acids,” Zeitschrift für anorganische und allgemeine Chemie 649 (2023): e202200265, 10.1002/zaac.202200265.

[64] R. Menye‐Biyogo , F. Delpech , A. Castel , V. Pimienta , H. Gornitzka , and P. Rivière , “Ruthenium‐Stabilized Low‐Coordinate Phosphorus Atoms. p‐Cymene Ligand as Reactivity Switch,” Organometallics 26 (2007): 5091–5101, 10.1021/om7004854.

[65] D. C. Wannipurage , A. D. Chivington , Y. Losovyj , M. Pink , and J. M. Smith , “Synthesis and Reactivity of a Paramagnetic Iron Phosphaethynolate Complex,” Organometallics 44 (2025): 468–471, 10.1021/acs.organomet.4c00447.

[66] M. K. Sharma , B. Neumann , H.‐G. Stammler , D. M. Andrada , and R. S. Ghadwal , “Electrophilic Terminal Arsinidene‐Iron(0) Complexes With a Two‐Coordinated Arsenic Atom,” Chemical Communications 55 (2019): 14669–14672, 10.1039/C9CC08630G. 31746858

[67] F. Neese , “The SHARK Integral Generation and Digestion System,” Journal of Computational Chemistry 44 (2022): 381–396, 10.1002/jcc.26942. 35678278

[68] J. J. Scepaniak , T. D. Harris , C. S. Vogel , J. Sutter , K. Meyer , and J. M. Smith , “Spin Crossover in a Four‐Coordinate Iron(II) Complex,” Journal of the American Chemical Society 133 (2011): 3824–3827, 10.1021/ja2003473. 21366252

[69] H.‐J. Lin , D. Siretanu , D. A. Dickie , et al., “Steric and Electronic Control of the Spin State in Three‐Fold Symmetric, Four‐Coordinate Iron(II) Complexes,” Journal of the American Chemical Society 136 (2014): 13326–13332, 10.1021/ja506425a. 25157642

[70] CASSCF/NEVPT2 calculations indicate that the quintet state is 28 kcal/mol more stable than the triplet, and 36 kcal/ mol more stable than the singlet.

[71] B. Feng , L. Xiang , K. N. McCabe , L. Maron , X. Leng , and Y. Chen , “Synthesis and Versatile Reactivity of Scandium Phosphinophosphinidene Complexes,” Nature Communications 11 (2020): 2916–2916, 10.1038/s41467-020-16773-w. PMC7283324 32518314

[72] A. Hinz , M. M. Hansmann , G. Bertrand , and J. M. Goicoechea , “Intercepting a Transient Phosphino‐Arsinidene,” Chemistry–A European Journal 24 (2018): 9514–9519, 10.1002/chem.201802175. 29723432

[73] A. Doddi , M. Weinhart , A. Hinz , et al., “N‐Heterocyclic Carbene‐Stabilised Arsinidene (AsH),” Chemical Communications 53 (2017): 6069–6072, 10.1039/C7CC02628E. 28466884

[74] C. Präsang , M. Stoelzel , S. Inoue , A. Meltzer , and M. Driess , “Metal‐Free Activation of EH_3_ (E=P, As) by an Ylide‐Like Silylene and Formation of a Donor‐Stabilized Arsasilene With a HSi═AsH Subunit,” Angewandte Chemie International Edition 49 (2010): 10002–10005, 10.1002/anie.201005903. 21104716

