# Supplementary material for: High Spin Iron–Phosphinidene and Arsinidene Complexes With Attenuated Metal–Ligand Multiple Bond Character
Source: Angew Chem Int Ed Engl. 2026 Mar 19;65(18):e23239. doi: 10.1002/anie.202523239 (PMC13110769; doi:10.1002/anie.202523239)
Supplement: Supplementary file 1 — Supporting File 1: Additional experimental details, characterization data (NMR, 57Fe Mössbauer, IR, UV–vis, mass spectrometry) and computational details (pdf). DFT optimized coordinates (xyz). The authors have cited additional references within the Supporting Information [75–88]. [file ANIE-65-e23239-s002.docx]

Supporting Information for

**High Spin Iron–Phosphinidene and Arsinidene Complexes with Attenuated Metal–Ligand Multiple Bond Character**

*Austin D. Chivington,*^†^ *Álvaro García-Romero*^†^*, David C. Meier*^†^*, Maren Pink,* ^†^ *Jose M. Goicoechea* ^†^ *and Jeremy M. Smith* ^†^

^†^ Department of Chemistry, 800 E. Kirkwood Ave, Indiana University, Bloomington IN 47405, USA

Table of Contents

[Experimental S3](#_Toc210993157)

[Synthesis of Complexes S4](#_Toc210993158)

[Reactivity S10](#_Toc210993159)

[NMR Spectra S11](#_Toc210993160)

[IR Spectra S27](#_Toc210993161)

[UV-Vis Spectra S32](#_Toc210993162)

[^57^Fe Mössbauer Spectra S36](#_Toc210993163)

[High Resolution Mass Spectrometry S38](#_Toc210993164)

[Computational Details S39](#_Toc210993165)

[X-Ray Crystallography S53](#_Toc210993166)

[References S79](#_Toc210993167)

# Experimental

***Safety Note: Hydrolysis of phosphinidene and arsinidene complexes, precursors, and intermediates releases phosphine and arsine gases. Caution should be taken in all manipulations to mitigate risk of exposure. Arsenic containing waste should be disposed of with care and in accordance with local environmental health and safety protocols.***

**General Considerations**

All manipulations involving air- or moisture sensitive compounds and their preparation were performed under an inert atmosphere of dry N_2_ in an M. Braun glovebox or by standard Schlenk techniques. Glassware was oven dried for at least 12 h at 140 °C before use. Celite was oven dried for 12 h at 200 ˚C. All solvents were purchased from Sigma-Aldrich and used after being dried using alumina and Q5 drying columns. [Na(18-crown-6)][PH_2_] and NaPH_2_ was prepared via reported protocol.^[75]^ The compounds PhB(AdIm)_3_FeCl, anhydrous pyridinium hydrochloride, 2-chloro-1,3-bis(2,6-diisopropylphenyl)-1,3,2-diazaphospholidine and benzyl potassium were prepared according to literature procedures.^[75-79]^ Deuterated solvents were purchased from Cambridge isotope labs. C_6_D_6_ and THF-d_8_ were degassed and stored over molecular sieves for at least one day before use. Trimethylsilyl chloride and benzyl bromide were degassed and distilled and dried over sieves prior to use. 18-crown-6 was dried by three sequential recrystallizations from MeCN under strictly anhydrous conditions. All other compounds purchased from commercial vendors and used as received. Arsenic containing compounds were destroyed in a well-ventilated hood by decanting into/or washing with a solution of sodium hypochlorite.

^1^H NMR and ^31^P NMR spectroscopic measurements of air and moisture sensitive compounds were made in J-Young NMR tubes, with the spectroscopic data recorded on Varian 400 MHz NMR and Bruker 500 MHz NMR spectrometers at 25 ˚C. UV-visible spectra were recorded with an Agilent Cary 60 UV-visible spectrometer. IR spectra were recorded with a Thermo-Fischer Spectrometer with an Everest ATIR accessory.

Mössbauer spectra were recorded on a SEE Co (Edina, MN; https://www.seeco.us/) spectrometer. The sample temperature was controlled using an SVT-400 Dewar from Janis equipped with a Lake Shore 255 Temperature Controller. Zero-field ^57^Fe Mössbauer spectra were collected with a constant acceleration spectrometer and a ^59^Co/Rh source over a 8 mm s^−1^ window. Prior to the measurements, the spectrometer was calibrated at 298 K with 30 µm thick α-Fe foil. Samples were prepared in a dry N_2_-filled glovebox. A typical sample was prepared by adding solid material suspended in Aflonx cryoprotectant to a circular polyethylene holder of 1 cm^2^ area which is sealed with a cap that is coated with a layer of silicon grease. The sample was frozen in liquid nitrogen prior to handling in air. Isomer shifts are reported relative to the centroid of the Fe metal spectrum recorded at 80 K, and quadrupole doublets were fitted to Lorentzian lineshapes. Data analysis was performed using the program WMOSS Mössbauer Spectral Analysis Software ([www.wmoss.org](http://www.wmoss.org)).

# Synthesis of Complexes

**PhB(AdIm)_3_FePH_2_ (1).** In the glovebox, a 20 mL scintillation vial was charged with PhB(AdIm)_3_FeCl (37 mg, 0.048 mmol) dissolved in 5 mL THF. A solution of [Na(18-crown-6)][PH_2_] (20 mg in minimal THF, 0.047 mmol) was added with stirring. The colorless solution immediately evolved to a golden yellow color. The reaction was stirred for 10 mins then dried, extracted with 5 mL benzene, and filtered through a plug of Celite and the volatiles removed *in vacuo*. Yellow crystals suitable for single crystal X-ray diffraction were grown from a concentrated solution of THF (*ca.* 1 mL) stored at -30° C overnight (33 mg, 90% yield). ^1^H NMR (THF-d_8_, 400 MHz): δ 66 (3H, Im-H), 50 (3H, Im-H), 39 (2H, B(C_6_H_5_) *o*/*m*-H), 16 (2H, B(C_6_H_5_) *o*/*m*-H), 14 (1H, B(C_6_H_5_) *p*-H), -24 (Ad, 15H) IR (ATIR) ν_PH2_ = 2265, 2255 cm^-1^; µ_eff_ =4.9(2) µ_B_ (Evans’ method). UV-Vis (THF, 0.38 mmol) λ_max_ = 417 nm, ε = 702 M^-1^ cm^-1^. HRMS ESI-MS: m/z [M+H]^+^ Calcd for C_45_H_59_N_6_BFeP 781.3975 Found 781.3981. *Nota Bene:* Crown-free NaPH_2_ can also be employed for large scale preparations or for preparing the crypt variant.


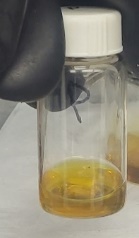


**Figure S1.** THF solution of **1**.

**[K(18-crown-6)] [PhB(AdIm)_3_FePH] (3-crown).** In the glovebox, a 20 mL scintillation vial was charged with PhB(AdIm)_3_FePH_2_ (40 mg, 0.051 mmol) dissolved in 5 mL THF. 18-crown-6 (13.5 mg, 0.051 mmol) in THF (2 mL) was added. An excess of benzyl potassium (13.6 mg, 0.104 mmol) in minimal THF (~2 mL) was added all at once while rapidly stirring at room temperature. The solution immediately became dark red. The reaction was stirred for 10 mins and then the THF is concentrated to *ca*. 1 mL. Pentane (10 mL) is added to the concentrate facilitating the precipitation of a dark red solid. The orange supernatant is decanted, and the red powder is washed with pentane until the pentane ran clear. The product is crystallized as red blocks from a concentrated THF solution at -30° C for 2 d. (36 mg recovered, yield 63%). ^1^H NMR (THF-d_8_, 400 MHz): δ 72 (3H, Im-H), 62 (3H, Im-H), 45 (2H, B(C_6_H_5_) *o*/*m*-H), 22 (2H, B(C_6_H_5_) *o*/*m*-H), 19 (1H, B(C_6_H_5_) *p*-H), 9 (24 H, K(18-crown-6)), -48 (Ad, 15H) IR (ATIR) ν_PH_ = 2215 cm^-1^; µ_eff_ =4.9(4) µ_B_ (Evans’ method). Due to the high thermal instability of the complex, we were unable to obtain elemental analysis data. Similarly, attempts to obtain mass spectral data were unsuccessful.


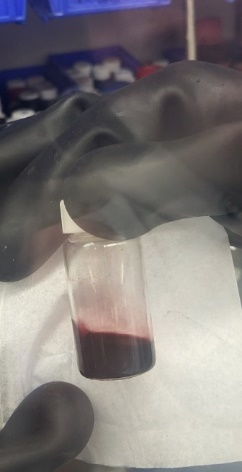


**Figure S2.** THF solution of **3-crown**.

**[K(2,2,2-Crypt)][PhB(AdIm)_3_FePH] (3-crypt).** In the glovebox, a 20 mL scintillation vial was charged with PhB(AdIm)_3_FePH_2_ (31 mg, 0.042 mmol) dissolved in 5 mL THF. 2,2,2-crypt (15.9 mg, 0.042 mmol) in THF (2 mL) was added. Then, at room temperature, benzyl potassium (11 mg 0.084 mmol) in THF (2 mL) was added at once. The solution was then concentrated *in vacuo.* The complex is crystallized as red blocks from concentrated THF at -30 °C. Once crystallized, the complex is insoluble in THF and benzene. The ^1^H NMR spectrum was recorded from an *in situ* prepared sample prior to crystallization. The solution magnetometry could not be measured due to the insolubility of the complex. Recovered 28 mg (Yield 58%). ^1^H NMR (THF-d_8_, 400 MHz): δ 87 (3H, Im-H), 36 (3H, Im-H), 15.8 (2H, B(C_6_H_5_) *o*/*m*-H), 10.5 (2H, B(C_6_H_5_) *o*/*m*-H), 9.7 (1H, B(C_6_H_5_) *p*-H), 3.60 (23H, 2,2,2-crypt obscured by solvent peak), 3.1 (9 H, 2,2,2-crypt), -13 (Ad, 15H) IR (ATIR) ν_PH_ =2212 cm^-1^. *Nota Bene*: The crypt complex is exceedingly sensitive to protonation. This is evident by ATR-IR where Fe-PH_2_ bands are observed likely due to reaction with adventitious moisture on the surface of the sample holder. Due to thermal instability and lack of solubility of crystallized samples elemental analysis nor mass spectrum could not be obtained.

**KAsH_2_. Caution! KAsH_2_ hydrolyzes violently to form AsH_3_**.

Potassium metal (1.66 g, 42.63 mmol) was melted in a 250 mL Schlenk flask fitted with a J-Young valve to form a mirror. In the glovebox, arsenic powder (1.00g, 13.36 mmol) was added to the Schlenk flask. With stirring, ~150 mL of DME is added along with naphthalene (85 mg, 0.66 mmol) dissolved in *ca.* 10 mL DME. The flask was sealed and the slurry stirred for 2 d at room temperature, by which time the solution had become dark brown and the potassium mirror had been consumed. The flask was connected a Schlenk line and a bubbler that vented through a bleach wash bottle at the back of the fume hood. The flask was cooled in an ice bath while flowing a stream of argon through the bubbler and wash bottle. Anhydrous anhydrous ethanol (1.23 g, 26.73 mmol) was added dropwise from a syringe through a septum. The reaction was removed from the ice bath and stirred for 1 h. At this point, a suspension of white and black solids in a yellow solution was observed. The flask was sealed and transferred back to the glovebox. The slurry was filtered through Celite to afford a clear yellow-orange solution. The volatiles were removed *in vacuo* to afford a yellow solid that was washed with pentane to give a free-flowing yellow-orange solid (78% yield). ^1^H NMR (THF-d_8_, 400 MHz): δ -2.74 (2H, As-H_2_). Due to safety concerns, the mass spectrum of this compound was not measured.

**[Na(18-crown-6)]_1.3_[NaAsH_2_]** **Caution! NaAsH_2_ hydrolyzes to form AsH_3_**.

Small pieces of sodium (*ca*. 0.5 cm^3^) cut into thin shards (464 mg, 0.020 mol), arsenic powder (501 mg, 0.006 mol) and naphthalene (40 mg, 0.312 mmol) were combined in a Schlenk flask. DME (50 mL) was added, which immediately formed a dark green/black solution. The mixture was stirred at 70 °C for 3 d, leading to a dark solution and black precipitate. *tert*-Butanol (1.2 mL) was added to this suspension via syringe at 0 °C. The suspension was stirred for two hours at room temperature, then filtered through Celite to provide a yellow solution. The yellow solution was transferred by cannula to a separate flask containing 18-crown-6 (5.25 g) in DME (10 mL). The resulting solution was concentrated and left to crystallize at room temperature (86 % yield). ^1^H NMR (THF-d_8_, 400 MHz): δ -2.66 (2H, As-H_2_).

**PhB(AdIm)_3_FeAsH_2_ (3).**

In the glovebox, a 20 mL scintillation vial was charged with PhB(AdIm)_3_FeCl (24 mg, 0.031 mmol) and THF (5 mL). A solution of KAsH_2_ (3.7 mg, 0.031 mmol) in THF (*ca*. 2 mL) was added with stirring. The colorless solution immediately became golden yellow. The reaction was stirred for 30 mins and then dried *in vacuo*. The residue extracted with benzene (5 mL), filtered through Celite and solvent removed *in vacuo*. The resulting yellow solid was then washed with pentane until clear. Crystals suitable for single crystal X-ray diffraction were grown from a concentrated solution of THF (*ca*. 1 mL) at -30 °C over the course of 1 week (75 % yield). ^1^H NMR (THF-d_8_, 400 MHz): δ 67 (3H, Im-H), 49 (3H, Im-H), 30 (2H, B(C_6_H_5_) *o*/*m*-H), 16 (2H, B(C_6_H_5_) *o*/*m*-H), 14 (1H, B(C_6_H_5_) *p*-H), -23 (Ad, 15H) IR (ATIR) ν_AsH2_ = 2068, 2053 cm^-1^; µ_eff_ =4.8(8) µ_B_ (Evans’ method). Due to safety concerns associated with the formation of arsine, the mass spectrum of this compound was not measured. [NaAsH_2_][Na(18-crown-6)]_1.3_ can also be employed in the procedure, providing a product with identical spectroscopic features (95 % yield).

**
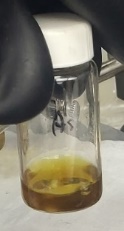
**

**Figure S3*.*** THF solution of **3**.

**[K(18-crown-6)][PhB(AdIm)_3_FeAsH] (4-crown).** A scintillation vial was charged with PhB(AdIm)_3_FeAsH_2_ (22 mg, 0.027 mmol), and 18-crown-6 (7.3 mg, 0.027 mmol) and dissolved in minimal THF (~10 mL). An excess of benzyl potassium (7.2 mg, 0.054 mmol) dissolved in minimal THF (~ 2 mL) was added, leading to a deep red-purple color. The reaction mixture was stirred for 10 mins and then concentrated *in vacuo* to *ca.* 1 mL. A 5 mL portion of pentane was then added to the concentrate to afford a red precipitate. The mixture was allowed to settle and the orange pentane/THF supernatant was decanted. The remaining red solid was washed with pentane (3 × ~5mL) and dried *in vacuo*. The product was crystallized as red blocks from concentrated THF at -30 °C over the course of 2 d. Isolated yield: 18 mg, 66%. ^1^H NMR (THF-d_8_, 400 MHz): δ 62 (3H, Im-H), 62 (3H, Im-H), 44 (2H, B(C_6_H_5_) *o*/*m*-H), 22 (2H, B(C_6_H_5_) *o*/*m*-H), 19 (1H, B(C_6_H_5_) *p*-H), 11 (24 H 18-crown-6), 1.4 (Ad, 7H), 1.2 (Ad, 2H), -1.7 (Ad, 9H), -4.5 (Ad, 9H) -24 (Ad, 15H) IR (ATIR) ν_AsH_ = 1958 cm^-1^; µ_eff_ =4.9(8) (Evans’ method).

**
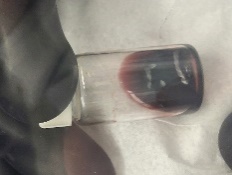
**

**Figure S4.** THF solution of **4-crown**.

**[K(2,2,2-Crypt)][PhB(AdIm)_3_FeAsH] (4-crypt).** A scintillation vial was charged with PhB(AdIm)_3_FeAsH_2_ (27 mg, 0.033 mmol), 2,2,2-cryptand (12 mg, 0.033 mmol) and minimal THF (~2 mL). Benzyl potassium (4.5 mg, 0.034 mmol) dissolved in minimal THF(~1 mL) was added, leading to a deep purple color. The reaction was stirred for 10 min and the solvent concentrated to *ca.* 1 mL. Pentane (5 mL) was added to induce the precipitation of dark purple material. The mixture was then allowed to settle and the orange pentane/THF supernatant was decanted. The remaining purple solid was washed with pentane (3 × ~5mL) until it ran clear, then dried *in vacuo*. Crystals suitable for single crystal X-ray diffraction were obtained by storing a THF (*ca.* 3 mL) solution at -30° C overnight. Isolated yield: 34 mg, 83%. ^1^H NMR (THF-d_8_, 400 MHz): δ 70 (3H, Im-H), 62 (3H, Im-H), 43 (2H, B(C_6_H_5_) *o*/*m*-H), 21 (2H, B(C_6_H_5_) *o*/*m*-H), 18 (1H, B(C_6_H_5_) *p*-H), 4.67 (36 H 18-crown-6), -45 (Ad, 15H). IR (ATIR) ν_AsH_ = 1958 cm^-1^; µ_eff_ =5.0(1) (Evans’ method). UV-Vis. λ_max_ = 568 nm, ε = 2264 L mol^-1^ cm^-1^ *Nota Bene*: As with the phosphinidene product, the crypt variant is exceedingly sensitive to protonation. This is evident by the ATR-IR adventitious moisture on the surface of the sample holder leads to the formation of ν_AsH2_ bands.


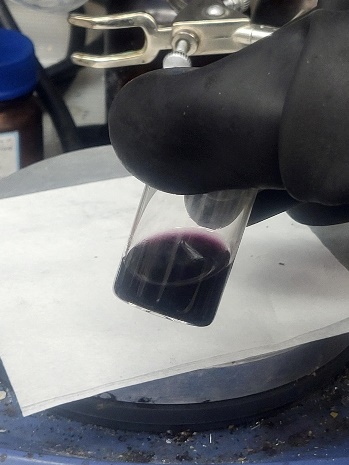


**Figure S5.** THF solution of **4-crypt**.

**Synthesis of [K(18-crown-6)][(DippNCH_2_)_2_PAsH].** A scintillation vial was charged with 2-chloro-1,3-bis(2,6-diisopropylphenyl)-1,3,2-diazaphospholidine (40.8 mg, 0.091 mmol) and THF (10 mL). The solution was cooled to -78 °C in the glovebox cold well, then a solution of [Na(18-crown-6)][AsH_2_] (33 mg, 0.090 mmol) in THF (5 mL) was added dropwise. The reaction was stirred at -78 °C for 15 min, leading to the formation of a light yellow solution along with a precipitate, presumably [Na(18-crown-6)][Cl]. Attempts to isolate and characterize the putative phosphinoarsanide DippNCH_2_)_2_PAsH_2_ product were unsuccessful due to the formation of a black solid (presumed to be metallic arsenic) as the solution warmed to room temperature.

Solid 18-crown-6 (24 mg, 0.090 mmol) was added to the solution, followed by the slow dropwise addition of benzyl potassium (11.9 mg, 0.091 mmol) in THF (5 mL). Complete consumption of each aliquot of orange benzyl potassium solution was observed before the next drop was added. The reaction was stirred at -78 °C for 15 min before the volatiles removed *in vacuo* to provide a golden yellow glass. The ^31^P{^1^H} NMR spectrum of this material reveals at least six phosphorus-containing products.

To purify the product, the solid was washed with pentane (3 × ~5 mL) and dried *in vacuo* to afford a sticky solid. This residue was dissolved in benzene (2 mL) and filtered through Celite to remove [Na(18-crown-6)][Cl]. The benzene solution was dried *in vacuo* to give a sticky orange oil. Addition of ether induces the precipitation of a fine orange powder suspended in yellow ethereal solution. The orange powder was removed by filtration through Celite. The supernatant was concentrated to ~0.5 mL, layered with heptane and stored at -35 °C overnight to precipitate additional orange solid. The ether/heptane mixture was decanted, dried under vacuum and the residue redissolved in minimal diethylether. The solution was dried under vacuum to afford a yellow oil that was repeatedly extracted with heptane until no material remained. Cooling the heptane solution at -30 °C for 3 d provided X-ray quality crystals of [K(18-crown-6)][(DippNCH_2_)_2_PAsH] *Safety Note*: It is likely that arsine condensed in the vacuum trap during workup, as exposure to air/moisture resulted in the immediate formation of black material along emanation of a vile and bitter perfume. The trap should be cleaned immediately with bleach in a well-ventilated area.

^1^H NMR (THF-d_8_, 500 MHz) δ = 6.97 (m, 6 H), 3.30 (m, 2 H), 3.13 (m, 4 H), 1.34 (d, J = 6.7 Hz, 6 H), 1.29 (d, J = 6.9 Hz, 6 H), 1.26 (d, J = 6.8 Hz, 6 H) 1.16 (d, J = 6.9 Hz, 6H). ^31^P{^1^H} NMR (THF-d_8_, 202 MHz) δ = 204.8 ppm. ^31^P NMR (THF-d_8_, 202 MHz) δ = 204.8 ppm (d *^2^*J_PH_ = 27.4 Hz). IR (ATIR) ν_AsH_ = 1958 cm^-1^. The mass spectrum was not recorded due to safety concerns.

# Reactivity

**Reaction with 2-chloro-1,3-bis(2,6-diisopropylphenyl)-1,3,2-diazaphospholidine**

Equimolar 2-chloro-1,3-bis(2,6-diisopropylphenyl)-1,3,2-diazaphospholidine was added to J-Young NMR tube containing a solution of [K(18-crown-6)][PhB(AdIm)_3_FeAsH] or [K(18-crown-6)][PhB(AdIm)_3_FePH] in THF-d_8_ (10 mg in 2 mL). The tube shaken. An equivalent of PPh_3_ equal to that of the starting iron complex was then added as an internal standard. The spectroscopic yields were determined by relative integration of the standard versus the product in the ^31^P{^1^H} NMR spectrum (see Figures S13, S14, S18, S19).

Yield of phosphinophosphinidene 54%

Yield of phosphinoarsinidene 42%

**Protonation of 3-crown and** **3-crypt**

Anhydrous pyridinium hydrochloride (17.5 mg , 0.015 mmol) was added to a J-Young NMR tube containing **3-crown** (16.5 mg, 0.015 mmol) in THF-d_8_. There was an immediate color change on mixing to a golden yellow solution. The formation of **1** was observed by ^1^H NMR spectroscopy along with another unidentified paramagnetic product (see supporting NMR Figure S20).

The analogous reaction with **3-crypt** and **4-crown** also afforded **1** and **2** as the major product of the reaction.

**Attempted Alkylation Reactions**

**Reaction of [K(18-crown-6)][PhB(AdIm)_3_FePH] with benzyl bromide**

Benzyl bromide (1.38 µL, 0.011 mmol) was added to a scintillation vial containing **3**-crown (13 mg, 0.0119 mmol) in THF-d_8_ (~0.5 mL). The solution immediately becomes golden yellow evolving to colorless and cloudy over 10 mins. The solution was then transferred to a J-Young NMR tube. A forest of paramagnetically-shifted resonances were observed in the ^1^H NMR spectrum, as well as resonances consistent with dibenzyl.

**Reaction of [K(18-crown-6)][PhB(AdIm)_3_FePH] with trimethylsilyl chloride**

Trimethylsilyl chloride (1.9 µL, 0.0016 mmol) was added to a vial containing a solution of **3**-crown (17.5 mg, 0.011 mmol) in THF (~0.5mL) inducing an immediate color change to golden yellow. Solution was then transferred to a J-Young NMR tube The ^1^H NMR spectrum of the crude material revealed the formation of **1** and PhB(AdIm)_3_FeCl.

Reactions with H_2_, CO_2_ and CO resulted in the formation of multiple paramagnetic products, none of which could be identified.

# NMR Spectra

**Figure S6.** ^1^H NMR spectrum (400 MHz, THF-d_8_, 25 °C) of PhB(AdIm)_3_FePH_2_ (1).


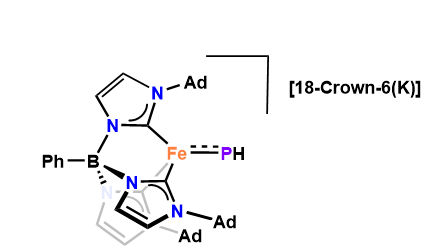


**Figure S7.** ^1^H NMR spectrum (400 MHz, THF-d_8_, 25 °C) of [K(18-crown-6)] [PhB(AdIm)_3_FePH].


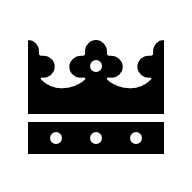


**Figure S8*.*** ^1^H NMR spectrum (400 MHz, THF-d_8_, 25 °C) of [K(18-crown-6)] [PhB(AdIm)_3_FePH] following 12 h in solution at RT. The resonance assigned to the crown ether shifts to higher field, consistent with THF solvation of the potassium ion.

**Figure S9.** ^1^H NMR spectrum (400 MHz, THF-d_8_, 25 °C) of crude [K(2,2,2-crypt)][PhB(AdIm)_3_FePH] (1).

**Figure S10.** ^1^H NMR spectrum (400 MHz, THF-d_8_, 25 °C) of KAsH_2_. ** Denotes pentane residue.

**Figure S11.** ^1^H NMR spectrum (400 MHz, THF-d_8_, 25 °C) of PhB(AdIm)_3_FeAsH_2_ * Denotes benzene residue from workup.

**Figure S12.** ^1^H NMR spectrum (400 MHz, THF-d_8_, 25 °C) of [K(18-crown-6)][PhB(AdIm)_3_FeAsH].


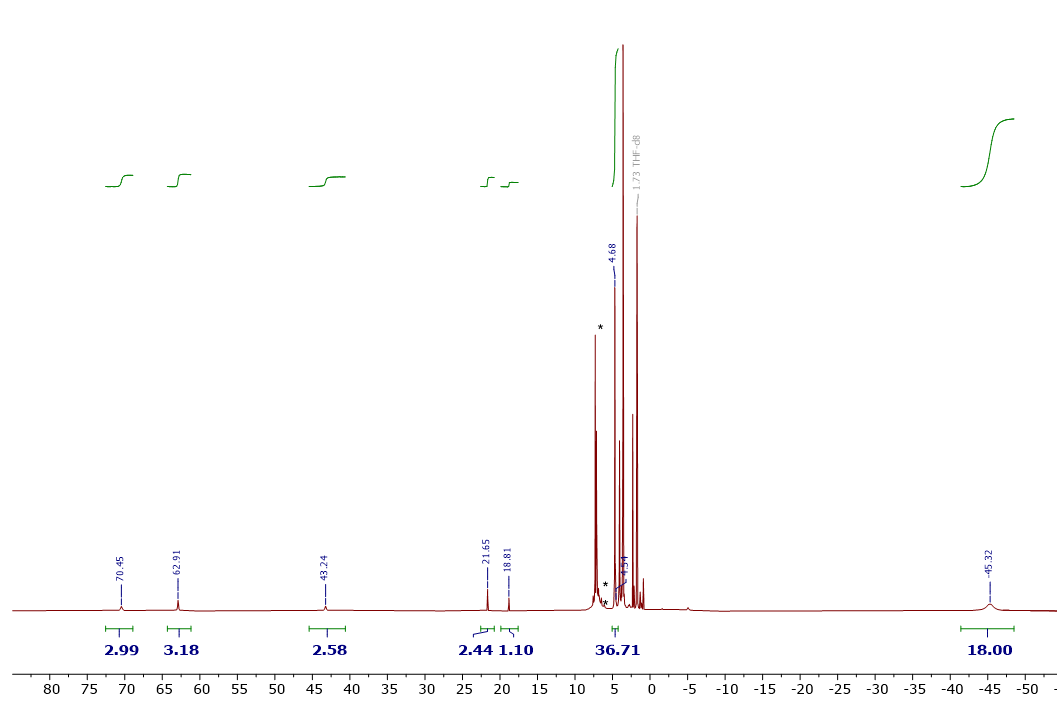


**Figure S13.** ^1^H NMR spectrum (400 MHz, THF-d_8_, 25 °C) of [K(2,2,2-crypt)][PhB(AdIm)_3_FeAsH]. * Denotes benzene residue from workup. **Denoted toluene residue from deprotonation.

**Figure S14.** ^31^P{^1^H} NMR of the rection of **3-crown** with 2-chloro-1,3-bis(2,6-diisopropylphenyl)-1,3,2-diazaphospholidine (202 MHz, THF-d_8_, 25 °C). δ = 181.8 ppm (d, ^1^J_P–P_ = 424.3 Hz) and -116.3 (d, ^1^J_P–P_ = 422.8 Hz). δ = 112.73 is unidentified material.

**Figure S15.** ^31^P NMR of the reaction of **3-crown** with 2-chloro-1,3-bis(2,6-diisopropylphenyl)-1,3,2-diazaphospholidine (202 MHz, THF-d_8_, 25 C). δ = 181.8 ppm (dd, ^1^J_PP_ = 424.3 Hz, ^2^J_PH_ = 38.2 Hz) and -116.3 (d, ^1^J_PP_ = 421.9 Hz, ^2^J_PH_ = 125.8 Hz. δ = 112.73 is unidentified material.

**Figure S16.** ^1^H NMR spectrum of [K(18-crown-6)][(DippNCH_2_)_2_PAsH] (500 MHz, THF-d_8_, 25 °C).

**Figure S17.** ^31^P{^1^H} NMR spectrum of [K(18-crown-6)][(DippNCH_2_)_2_PAsH] after purification (202 MHz, THF-d_8_, 25 °C). Unidentified resonances δ = 122.1 and 112.6 ppm.

**Figure S18.** ^31^P NMR spectrum of [K(18-crown-6)][(DippNCH_2_)_2_PAsH] (202 MHz, THF-d_8_, 25 °C). Peak of interest: δ = 204.8 ppm (d ^2^J_PH_ = 27.4 Hz).

*

*

**Figure S19.** ^31^P{^1^H} NMR spectrum of the reaction of **4-crown** with 2-chloro-1,3-bis(2,6-diisopropylphenyl)-1,3,2-diazaphospholidine (202 MHz, THF-d_8_, 25 °C).

**Figure S20.** ^31^P NMR spectrum of the rection of **4-crown** with 2-chloro-1,3-bis(2,6-diisopropylphenyl)-1,3,2-diazaphospholidine (202 MHz, THF-d8, 25 °C) Insets are zoomed-in on peaks. P-H coupling is only observed at δ (ppm) 204.37 (d, ^2^J _PH_ = 28.4 Hz).

**Figure S21.** ^1^H NMR spectrum of the reaction of **3-crown** with pyridinium hydrochloride (500 MHz, THF-d_8_, 25 °C). Labeled peaks are for **1.**

# IR Spectra

**Figure S22.** ATIR spectrum (solid-state, 25 °C) of [Na(18-crown-6)][AsH_2_].

**Figure S23.** ATIR spectrum (solid-state, 25 °C) of PhB(AdIm)_3_FePH_2_ (1).

**Figure S24.** ATIR spectrum (solid-state, 25 °C) of PhB(AdIm)_3_FeAsH_2_ (2).

**Figure S25.** ^1^ATIR spectra (solid-state, 25 °C) of PhB(AdIm)_3_FeAsH_2_ and of PhB(AdIm)_3_FePH_2_ overlayed.

**Figure S26.** ATIR spectrum (solid-state, 25 °C) of [K(18-crown-6)] [PhB(AdIm)_3_FePH] (3-crown).

**Figure S27.** ATIR spectrum (solid-state, 25 °C) of [K(2,2,2-crypt)][PhB(AdIm)_3_FePH] (3-crypt).

**Figure S28.** ATIR spectrum (solid-state, 25 °C) of [K(18-crown-6)][PhB(AdIm)_3_FeAsH] (4-crown).

**Figure S29.** ATIR spectrum (solid-state, 25 °C) of [K(2,2,2-crypt)][PhB(AdIm)_3_FeAsH][ (4-crypt).

**Figure S30.** ATIR spectrum (solid-state, 25 °C) of [K(18-crown-6)][(DippNCH_2_)_2_PAsH]

# UV-Vis Spectra

**Figure S31.** UV-Vis spectrum of PhB(AdIm)_3_FePH_2_ (0.38 mmol in THF) at 25 °C. λ_max_ = 417 nm, ε = 702 M^-1^ cm^-1^.

**Figure S32.** UV-Vis spectrum of [K(18-crown-6)][PhB(AdIm)_3_FePH] (0.30 mmol in THF) at 25 C. λ_max_ = 500 nm, ε = 2117 L mol^-1^ cm^-1^.

**Figure S33.** UV-Vis spectrum of [K(2,2,2-Crypt)][PhB(AdIm)_3_FePH], 0.24 mmol in THF at 25 C. λ_max_ = 567 nm, ε = 2279 L mol^-1^ cm^-1^.

**Figure S34.** UV-Vis spectrum of [K(2,2,2-Crypt)][PhB(AdIm)_3_FeAsH], 0.66 mmol in THF at 25 C. λ_max_ = 566 nm, ε = 1427 L mol^-1^ cm^-1^.

# ^57^Fe Mössbauer Spectra

**Figure S35.** Zero-field ^57^Fe Mössbauer spectrum of [K(18-crown-6)][PhB(AdIm)_3_FePH] at 80 K. Spectral parameters: δ = 0.63 mm s^−1^, ∆E_Q_ = 1.59 mm s^−1^. The spectral noise is attributed to the low concentration of iron in the sample.

**
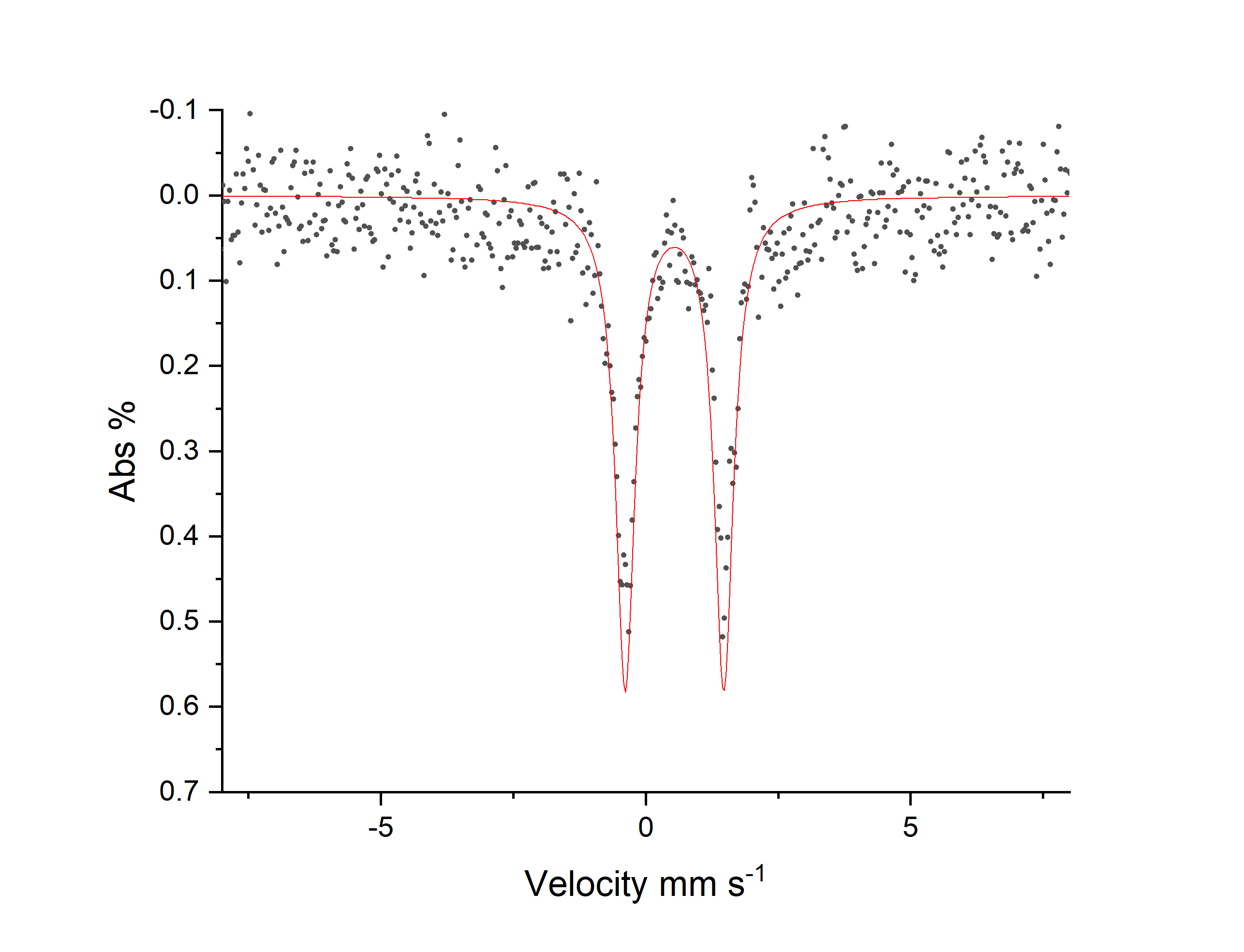
**

**Figure S36.** Zero-field ^57^Fe Mössbauer spectrum of [K(2,2,2-crypt)][PhB(AdIm)_3_FeAsH] measured at 80 K. Spectral parameters: δ = 0.51 mm s^−1^, ∆E_Q_ = 1.87 mm s^−1^. The spectral noise is attributed to the low concentration of iron in the sample.

# High Resolution Mass Spectrometry


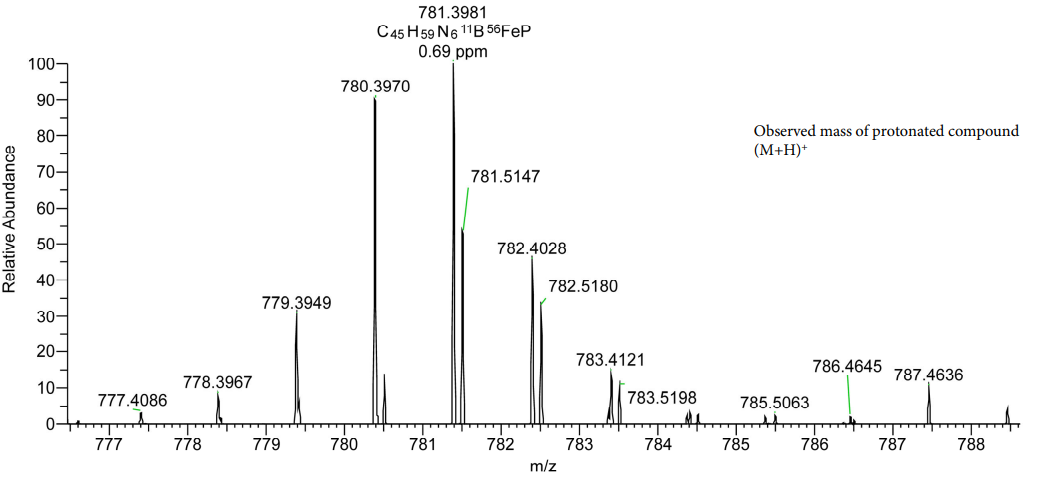


**Figure S37.** Observed HRMS of **1** [M+H]^+^.


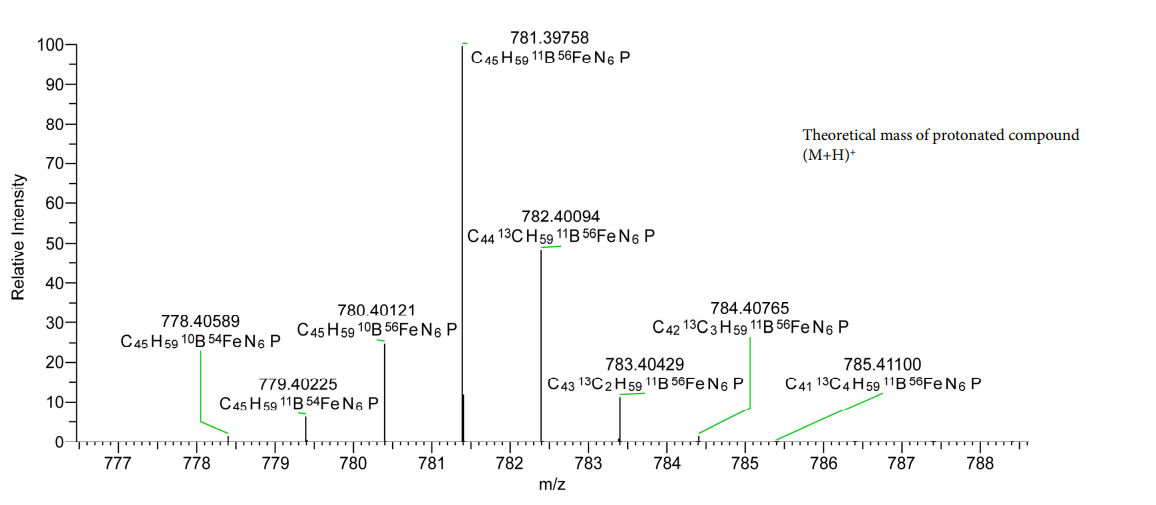


**Figure S38.** Theoretical HRMS of **1** [M+H]^+^.

# Computational Details

All calculations were performed using density functional theory or complete active space self-consistent field as implemented in the ORCA 6.0.1 computational software package.^[80-87]^ DFT computations used the molecular structures determined by single crystal X-ray diffraction with the positions of the hydrogen atoms optimized in the gas phase using the *r*^2^SCAN-3c composite electronic structure method. Linear synchronous transit (LST) calculations were also performed in the gas phase using *r*^2^SCAN-3c.{Grimme, 2021 #14} The geometries of other possible spin states spin (*S* = 0, 1) complexes were also optimized in the gas phase using *r*^2^SCAN-3c (**Table S1**).

Single point energies for the anionic phosphinidene and arsenidene complexes were calculated using the B3LYP functional,{Becke, 1993 #15;Stephens, 1994 #16} the minimally augmented triple zeta basis set ma-def2-TZVP and the def/J auxiliary basis set.{Weigend, 2006 #17;Weigend, 2005 #18;Zheng, 2011 #19} The ^57^Fe Mössbauer spectroscopic parameters were calculated with scalar zeroth-order level relativistic approximations (ZORA) using B3LYP/ma-ZORA-def2-TZVP and the decontracted def/J auxiliary basis set (SARC/J). The spectroscopic parameters were calibrated according to the method of Neese (**Table S2**).^[88]^

CASSCF calculations were performed using truncated complexes [HB(MeIm)_3_FeEH]^–^ (E = P, As) in which the adamantyl groups were replaced by methyls and the phenyl group by a proton. The truncated complexes were optimized in the gas phase (*S* = 2) using *r*^2^SCAN-3c with the Fe-E distances constrained to those observed in the crystal structures of **3-crown** and **4-crown**.

A number of active spaces were explored, but only the active space with 12 electrons in 8 orbitals (CASSCF(12,8)) provided ^57^Fe Mössbauer spectral parameters that agree with experimental values (**Table S2**). These calculations were performed without state averaging. As with the DFT calculations, the minimally augmented triple zeta basis set ma-def2-TZVP basis set was used for all atoms. The resolution of identity (RI) approximation for coulomb and HF-exchange (RIJK) in combination with the corresponding auxiliary basis set def2/JK{Wiegend, 2008 #28} was implemented to speed up the calculations. These calculations were used to determine the electronic configurations for the ground spin state (*S* = 2) in the absence of state averaging (Figures 5, ; **Table S4** and **Table S5** )

The relative energies of likely spin states were determined using state averaged complete active space self-consistent field (CASSCF(12,8)) calculations with 4, 11 and 10 roots for the quintet, triplet and singlet states, respectively. Dynamic electron correlation energies were then added using strongly contracted N-electron valence state perturbation theory (SC-NEVPT2), and computational cost was improved with the RI approximation. These calculations show that *S* = 2 is the ground state (**Table S3**).


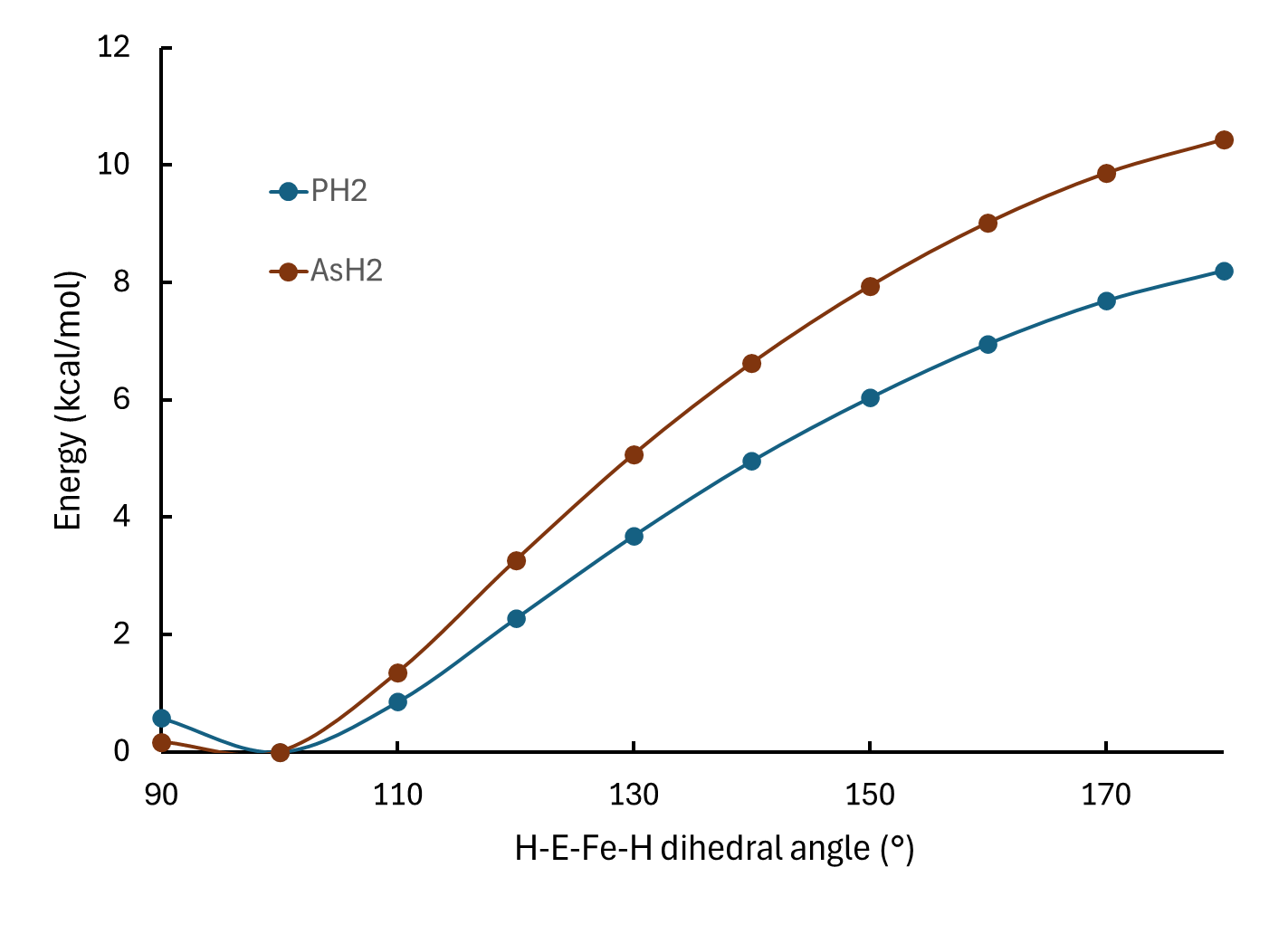


**Figure S39.** Linear synchronous transit (LST) scan for the H-E-Fe-H dihedral angle (E = P, As) in PhB(AdIm)_3_Fe(EH_2_). The LST maps the change in geometry of the EH_2_ ligand from pyramidal (H-E-Fe-H = 90 °) to planar (H-E-Fe-H = 180 °).


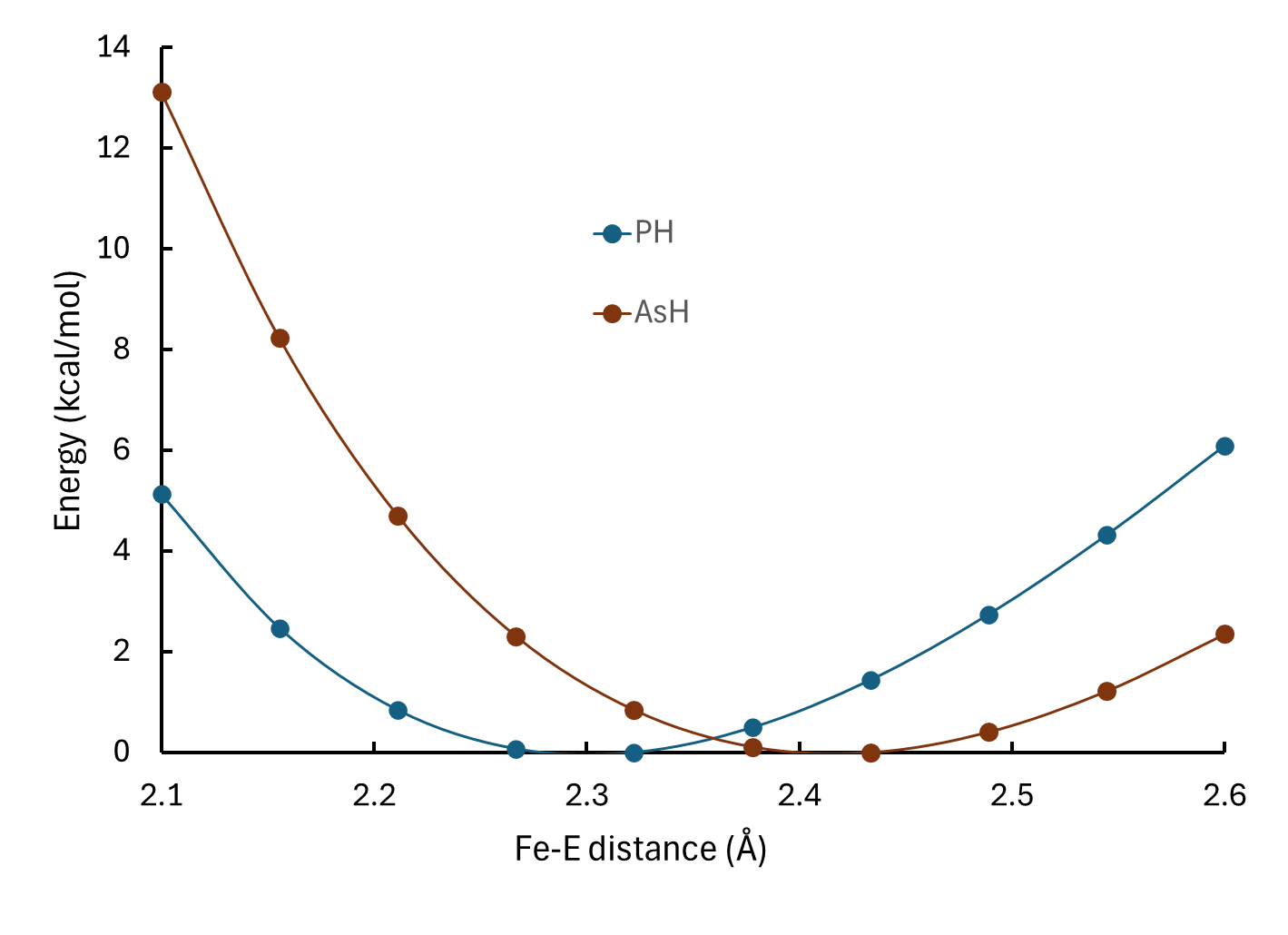


**Figure S40.** Linear synchronous transit (LST) scan for the Fe-E distance (E = P, As) in [PhB(AdIm)_3_Fe(EH)]^-^. The LST reveals the relatively shallow potential energy surface for the Fe-E bond length in both complexes.

**Table S1.** Comparison of selected metrical parameters for different spin states of [PhB(AdIm)_3_FeEH]^-^, as determined by DFT.

| E | *S* | Fe-E (Å) | Fe-C (Å) | B ···Fe-E (°) | Fe-E-H (°) |
| --- | --- | --- | --- | --- | --- |
| P | 0 | 2.071 | 1.913-1.934 | 179.8 | 179.2 |
|  | 2 | 2.301 | 2.097-2.120 | 176.9 | 104.5 |
|  | 2^a^ | 2.412(5) | 2.027(14) - 2.063(14) | 177.93(4) | 112.4 |
| As | 0 | 2.211 | 1.903-1.941 | 168.3 | 141.3 |
|  | 2 | 2.416 | 2.083-2.132 | 178.3 | 98.6 |
|  | 2^a^ | 2.4249(6) | 2.110(2)-1.167(2) | 177.24(4) | 108.3 |

^a^ Metrical parameters from the molecular structures of **3-crypt** and **4-crypt** determined by single crystal X-ray diffraction.

**Table S2.** Comparison of computed and experimental ^57^Fe Mössbauer spectral parameters.

| Compound | δ (mm/s) | Δ*E*_Q_ (mm/s) |
| --- | --- | --- |
| [PhB(AdIm)_3_FePH]^-^ | | |
| DFT^a^ | 0.61 | -1.60 |
| CASSCF^b^ | 0.58 | +1.81 |
| Experimental^c^ | 0.63 | \|1.76\| |
| [PhB(AdIm)_3_FeAsH]^-^ | | |
| DFT^a^ | 0.53 | +2.06 |
| CASSCF^b^ | 0.61 | +2.03 |
| Experimental^b^ | 0.51 | \|1.87\| |

^a^ Gas phase calculation using B3LYP/ma-ZORA-def2-TZVP.

^b^ Calculated using CASSCF(12,8) for the truncated complexes [HB(MeIm)_3_FeEH]^–^ (E = P, As).

^c^ Recorded at 80 K.


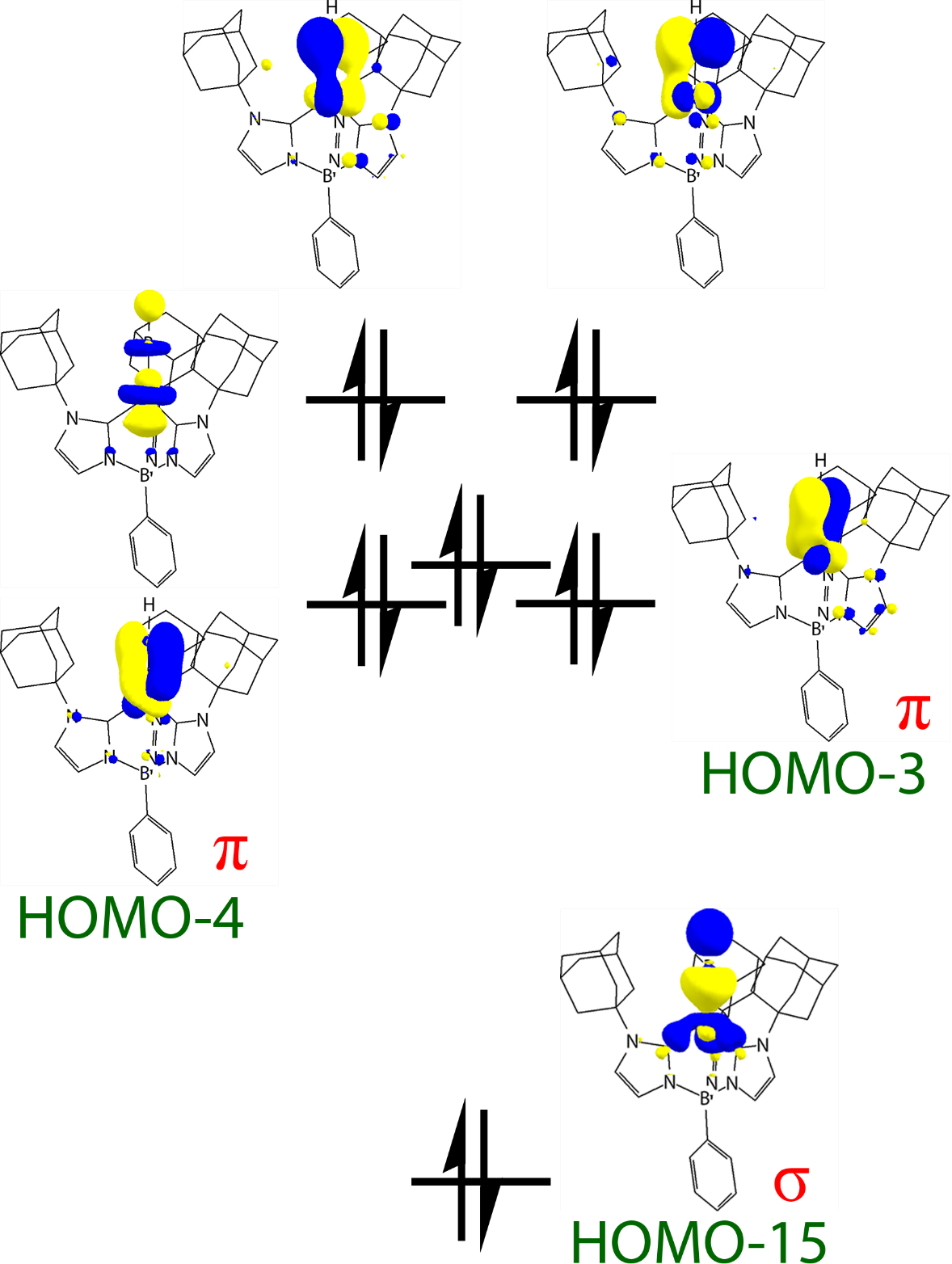


**Figure S41.** Selected frontier orbitals for the hypothetical complex [Ph(AdIm)_3_FePH]^-^ (*S* = 0) as determined by DFT (B3LYP/ma-def2-TZVP). The Fe-P π (HOMO-3, HOMO-4) and σ (HOMO-15) orbitals are doubly occupied. All the other iron d-orbitals are largely non-bonding. Note that there is symmetry-allowed mixing of the largely non-bonding HOMO and HOMO-1 with HOMO-3 and HOMO-4, respectively.


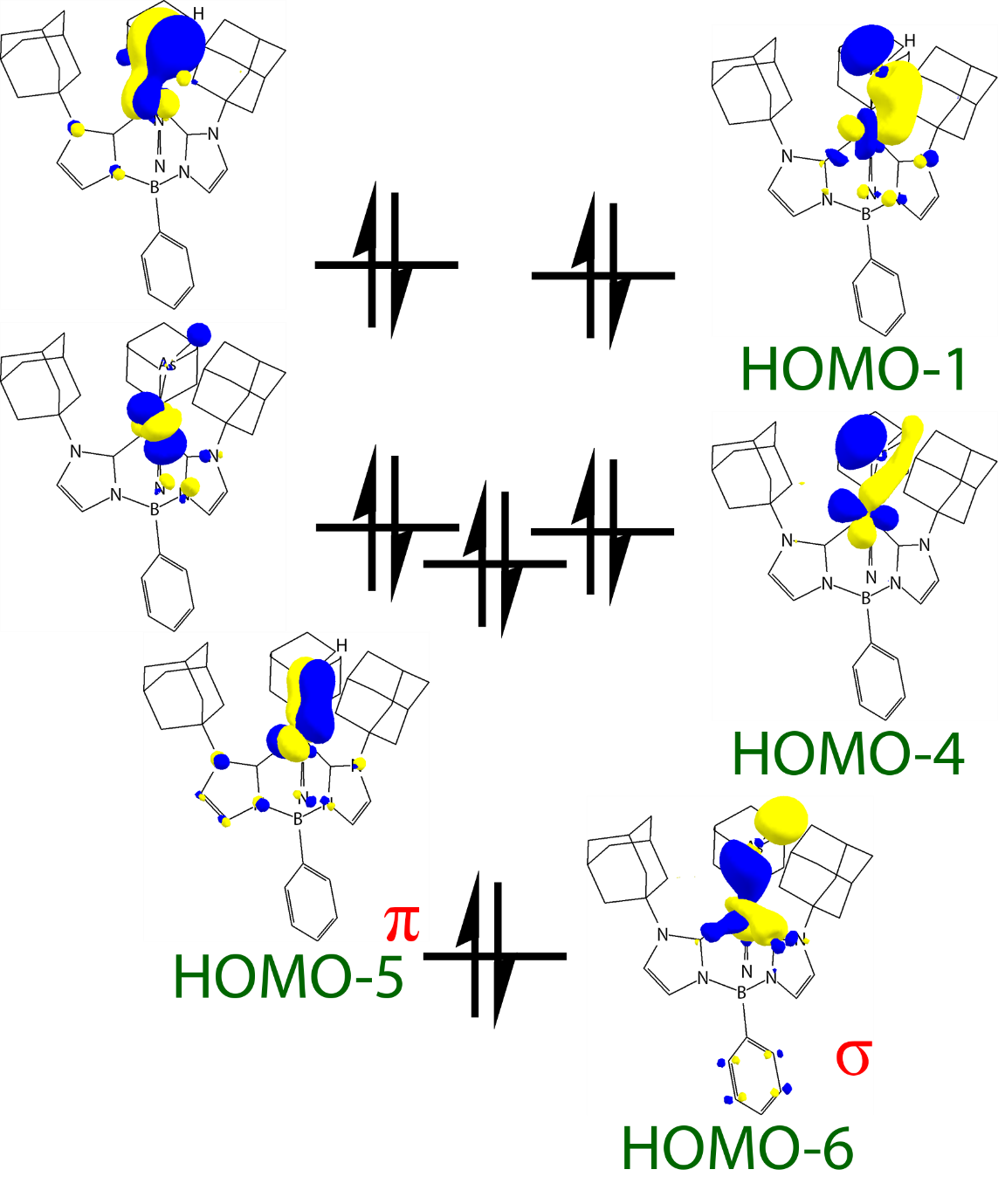


**Figure S42.** Selected frontier orbitals for the hypothetical complex [Ph(AdIm)_3_FeAsH]^-^ (*S* = 0) as determined by DFT (B3LYP/ma-def2-TZVP). The Fe-As π (HOMO-5) and σ (HOMO-6) orbitals are doubly occupied. There is As lone pair character in HOMO-4 and HOMO-1. All the other iron d-orbitals are largely non-bonding. Note that there is symmetry-allowed mixing of the largely non-bonding HOMO with HOMO-5.


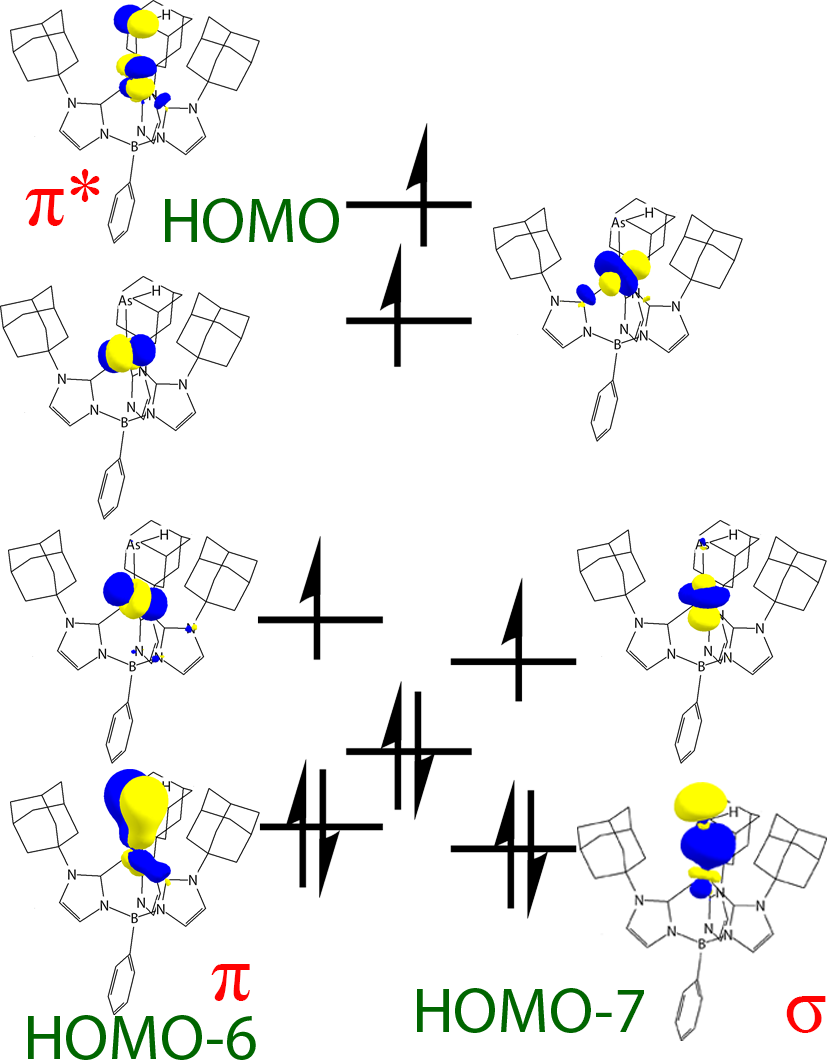


**Figure S43.** Natural orbital representation of the frontier orbitals for [Ph(AdIm)_3_FeAsH]^-^, as determined by DFT (B3LYP/ma-def2-TZVP). The Fe-As σ (SOMO-7) and π (SOMO-6) orbitals are both doubly occupied while the SOMO has Fe-As π* character. All the other iron d-orbitals are largely non-bonding.


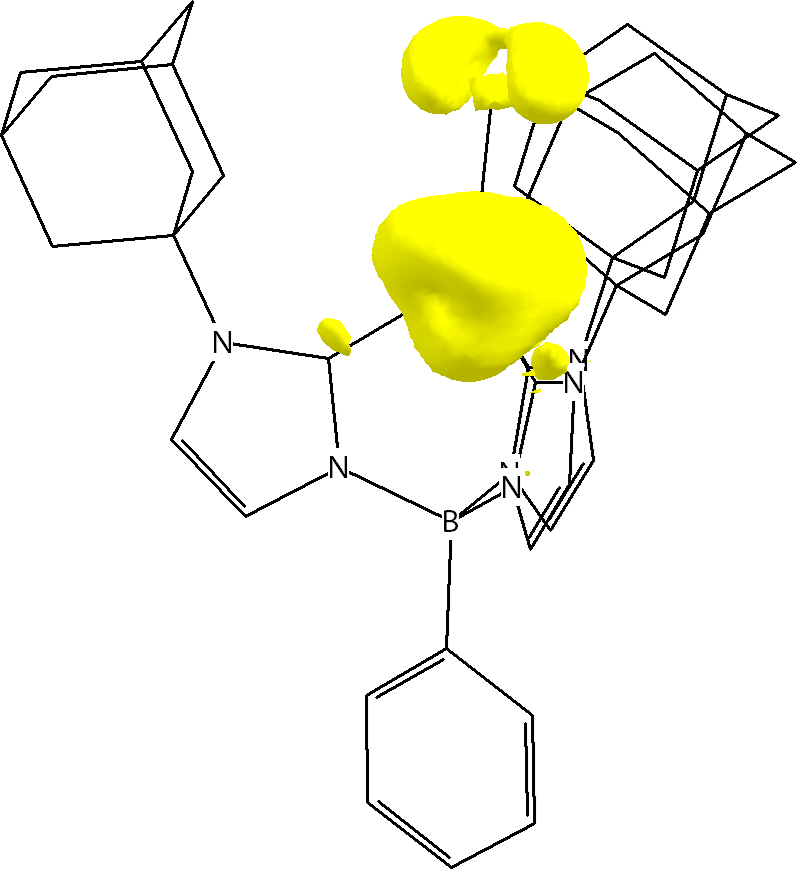


**Figure S44.** Spin density of [Ph(AdIm)_3_FePH]^-^, as determined by DFT (B3LYP/ma-def2-TZVP), isodensity at 0.005. Lowdin spin densities Fe: 3.45; As 0.28.


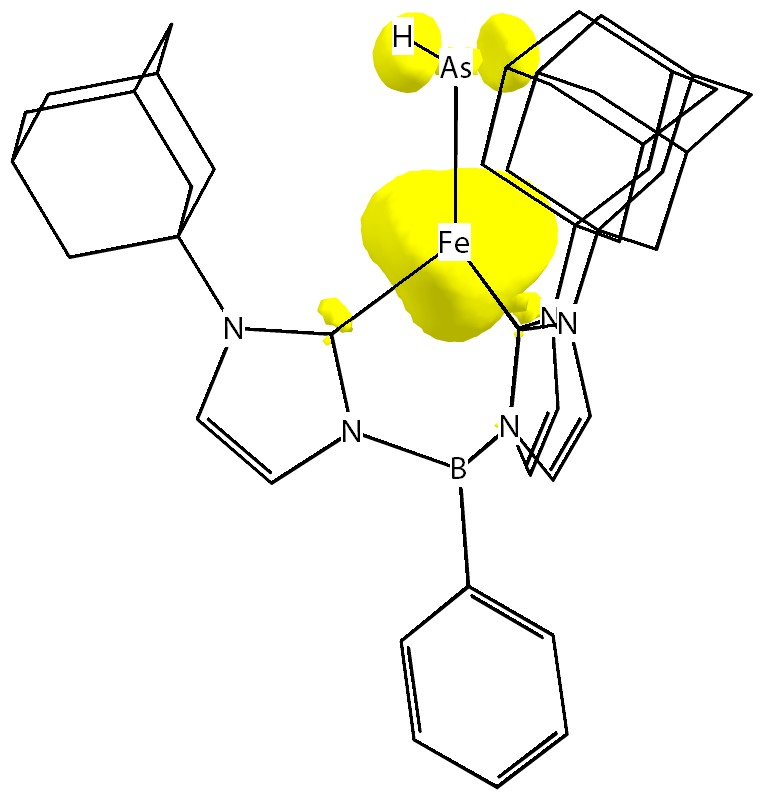


**Figure S45.** Spin density of [Ph(AdIm)_3_FeAsH]^-^, as determined by DFT (B3LYP/ma-def2-TZVP), isodensity at 0.005. Lowdin spin densities Fe: 3.45; As 0.28.


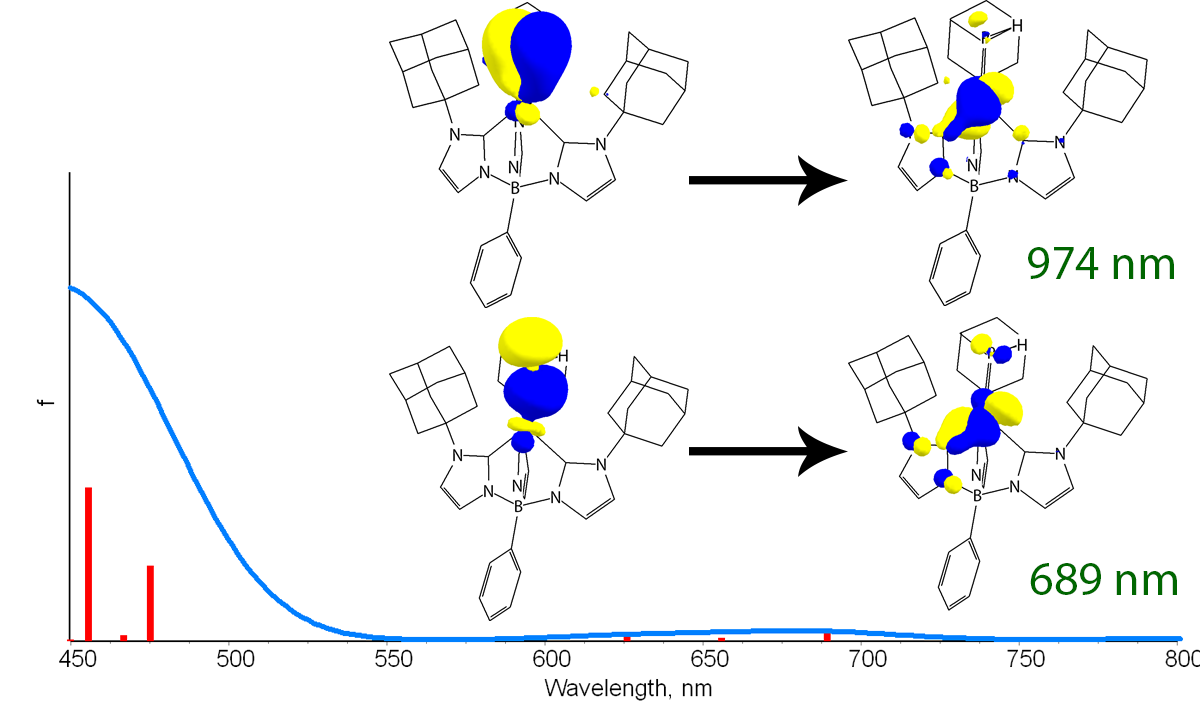


**Figure S46.** TD-DFT spectrum of [Ph(AdIm)_3_FePH]^-^, as determined by DFT (cam-B3LYP/ma-def2-TZVP). Natural transition orbitals for the major transitions at 974 and 689 nm are shown.


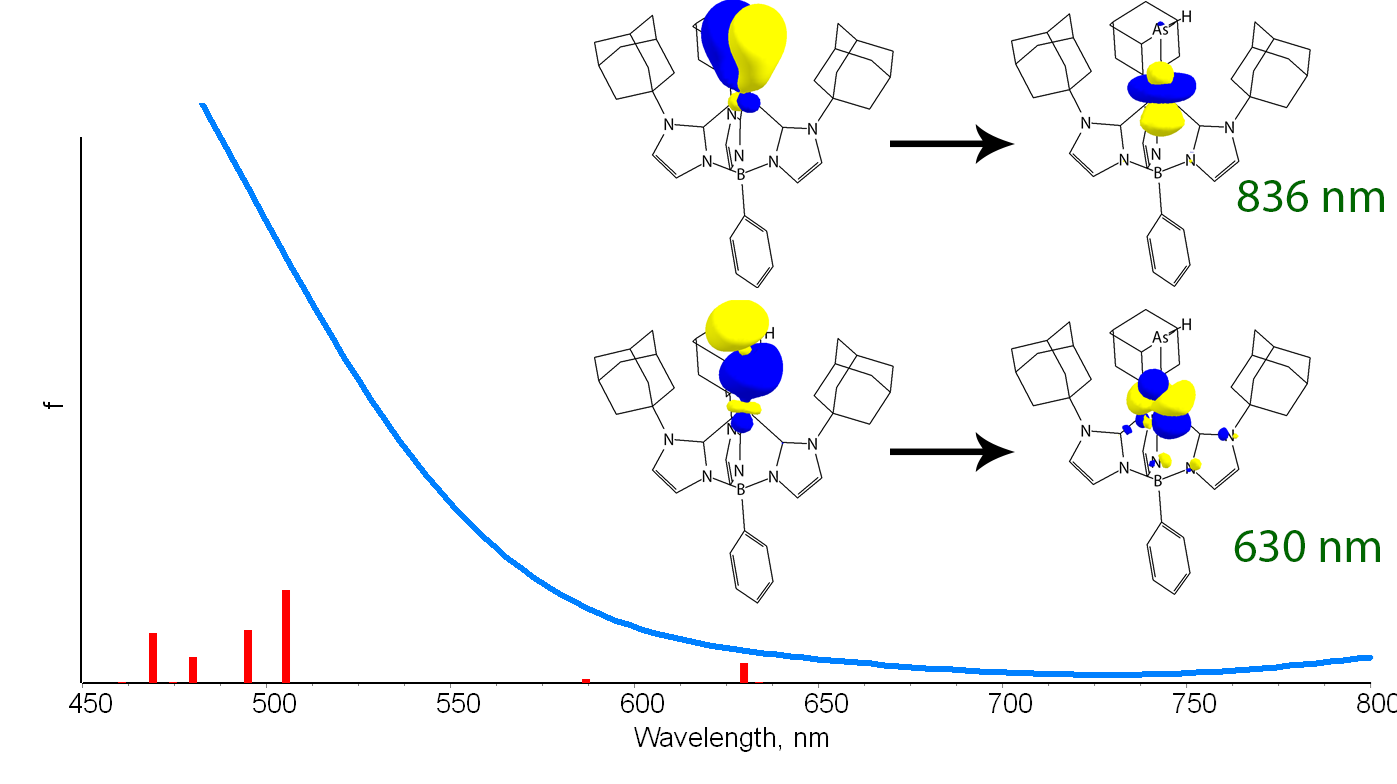


**Figure S47.** TD-DFT spectrum of [Ph(AdIm)_3_FeAsH]^-^, as determined by DFT (cam-B3LYP/ma-def2-TZVP). Natural transition orbitals for the major transitions at 836 and 630 nm are shown.

**Table S3.** Spin state energetics (kcal/mol) as determined from state averaged CASSCF(12,8)/NEVPT2 calculations

| Spin state | [HB(MeIm)_3_FePH]^–^ | [HB(MeIm)_3_FeAsH]^–^ |
| --- | --- | --- |
| *S* = 0 | 12.7 | 15.3 |
| *S* = 1 | 7.4 | 11.8 |
| *S* = 2 | 0 | 0 |

**Table S4.** Configuration of ground state of [HB(MeIm)_3_FePH]^–^ as determined by CASSCF(12,8) without state averaging.

| 0.61225 | [69]: | 22221111 |
| --- | --- | --- |
| 0.08624 | [64]: | 22122111 |
| 0.07961 | [44]: | 21122211 |
| 0.05178 | [67]: | 22211211 |
| 0.04852 | [40]: | 21121212 |
| 0.03343 | [60]: | 22112211 |
| 0.02717 | [61]: | 22121112 |
| 0.01309 | [56]: | 22111212 |
| 0.00804 | [66]: | 22211121 |
| 0.00623 | [43]: | 21122121 |
| 0.00589 | [59]: | 22112121 |
| 0.00581 | [47]: | 21211221 |
| 0.00477 | [52]: | 21221121 |
| 0.00406 | [39]: | 21121122 |
| 0.00387 | [53]: | 21221211 |

**Table S5.** Configuration of ground state of [HB(MeIm)_3_FeAsH]^–^ as determined by CASSCF(12,8) without state averaging.

| 0.61907 | [69]: | 22221111 |
| --- | --- | --- |
| 0.08383 | [67]: | 22211211 |
| 0.06840 | [44]: | 21122211 |
| 0.06015 | [64]: | 22122111 |
| 0.04957 | [40]: | 21121212 |
| 0.03594 | [60]: | 22112211 |
| 0.01633 | [61]: | 22121112 |
| 0.01408 | [56]: | 22111212 |
| 0.01347 | [66]: | 22211121 |
| 0.00626 | [47]: | 21211221 |
| 0.00507 | [53]: | 21221211 |
| 0.00416 | [43]: | 21122121 |
| 0.00413 | [52]: | 21221121 |
| 0.00335 | [59]: | 22112121 |
| 0.00329 | [35]: | 21111222 |


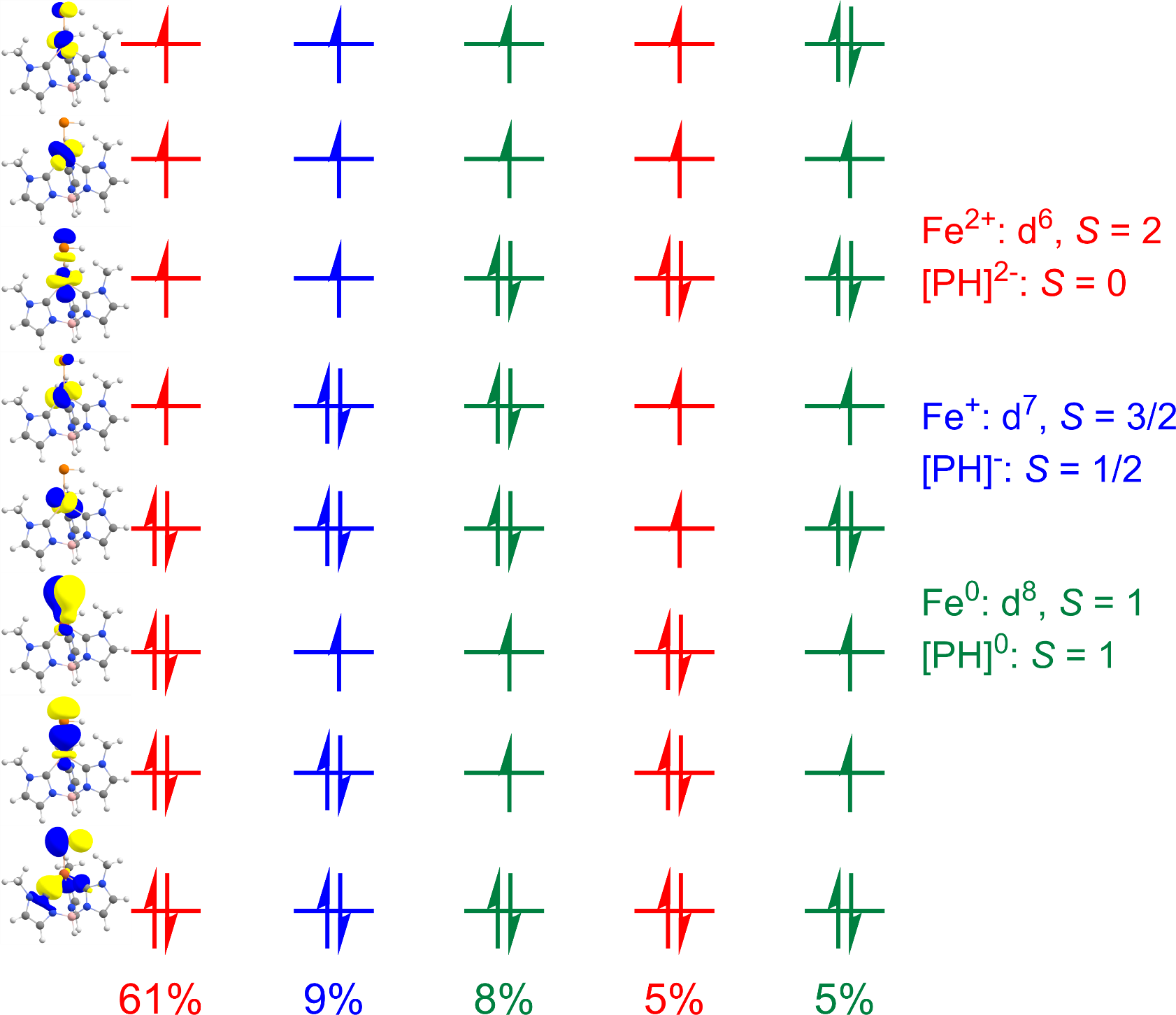


**Figure S48.** Most significant configurations from the CASSCF(12,8) calculations for the truncated complex [HB(MeIm)_3_FePH]^–^. Three smaller configurations that represent a total of 5% of the wavefunction, corresponding to the {Fe^0^-[PH]^0^} resonance structure are not shown. Isodensity 0.05


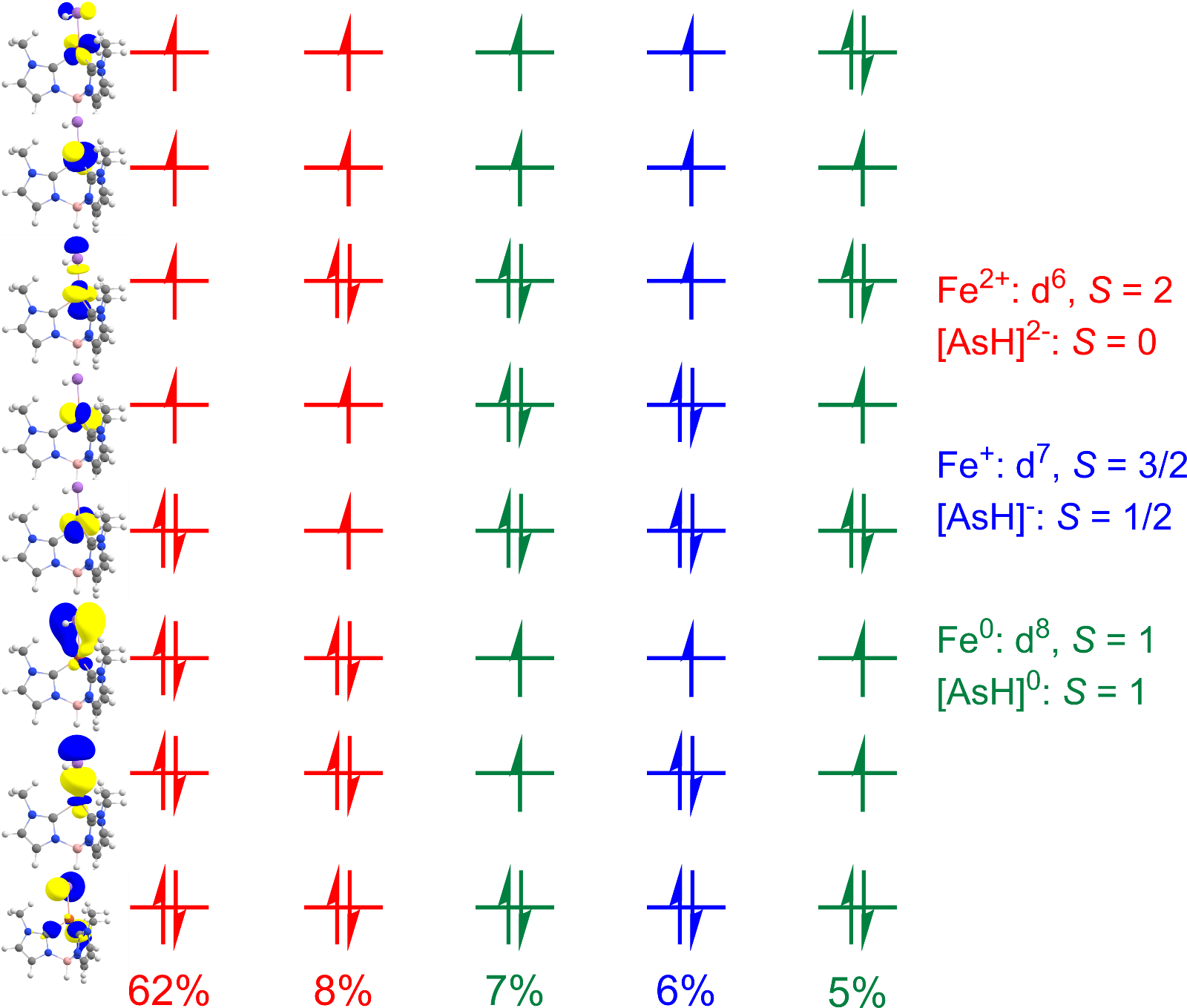


**Figure S49.** Most significant configurations from the CASSCF(12,8) calculations for [HB(MeIm)_3_FeAsH]^–^. Three smaller configurations that represent a total of 6% of the wavefunction, which correspond to the {Fe^I^-[AsH^-^]} resonance structure are not shown, as is one configuration representing 1% of the wavefunction that corresponds to the {Fe^II^-[AsH]^2-^} resonance structure. Isodensity 0.05.

# X-Ray Crystallography

**
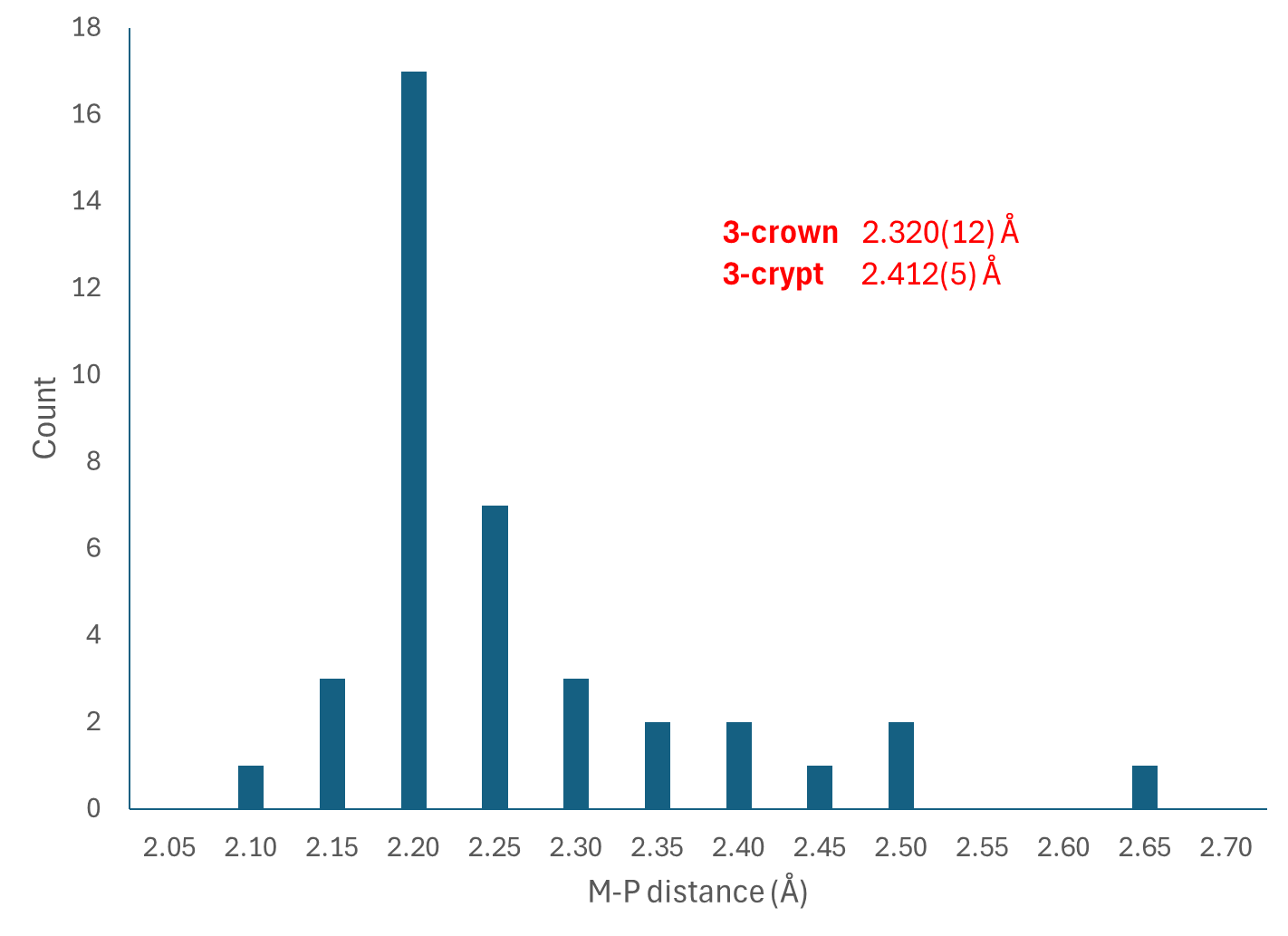
**

**Figure S50.** Distribution of M-P distances in transition metal complexes with terminal phosphinidene ligands from crystallographic data in the Cambridge Structural Database.


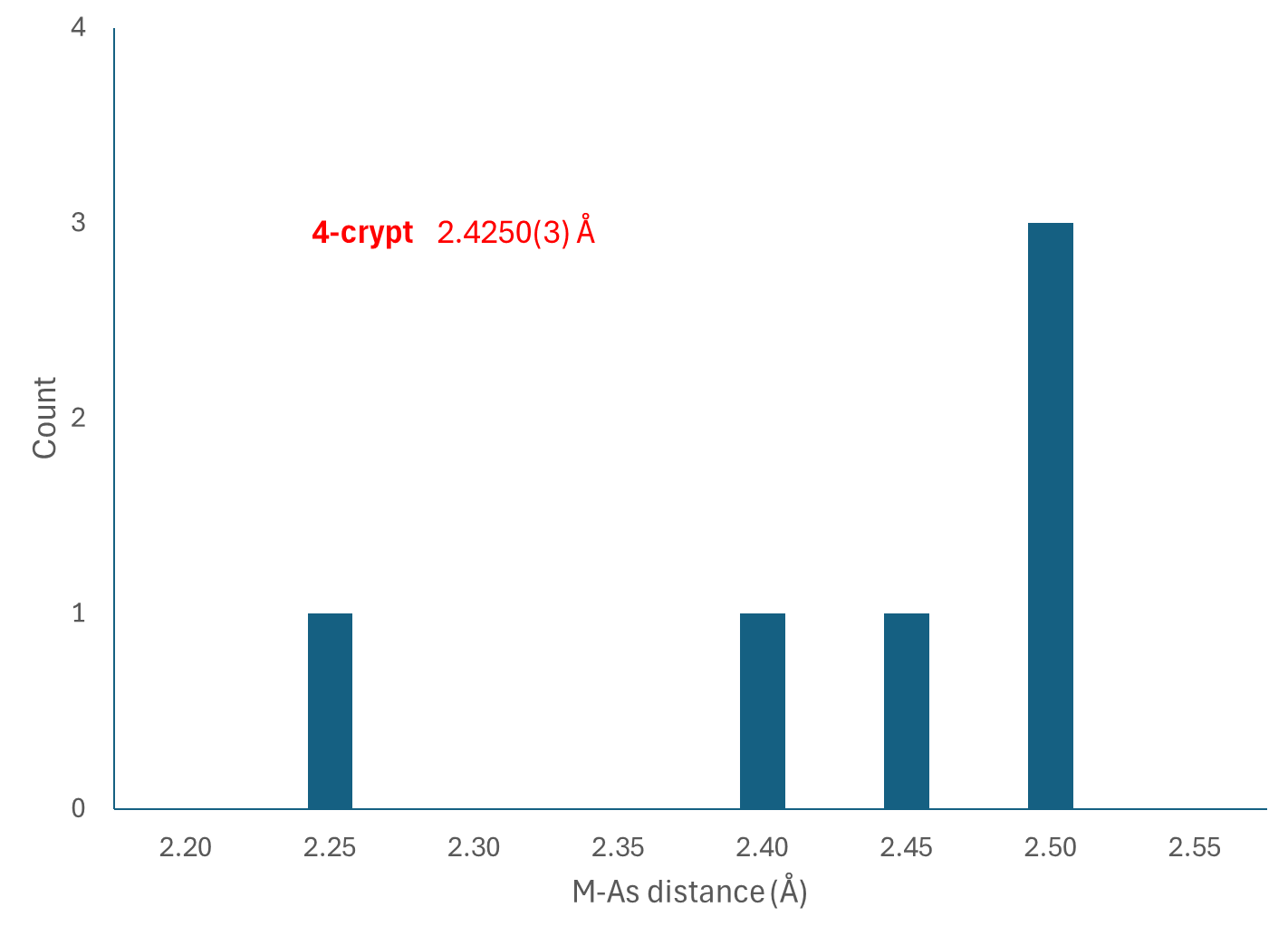


**Figure S51.** Distribution of M-As distances in transition metal complexes with terminal arsinidene ligands from crystallographic data in the Cambridge Structural Database.


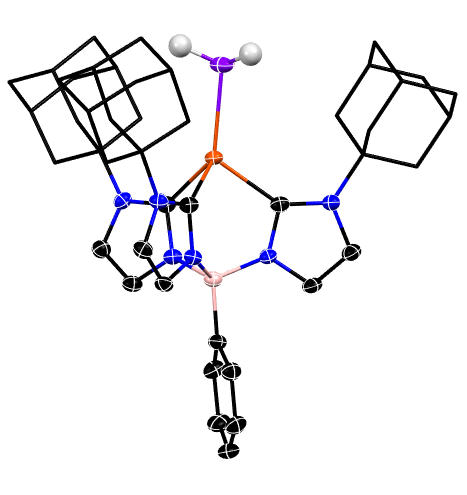


**Figure S52.** X-ray crystal structure of PhB(AdIm)_3_FePH_2_ (**1**); thermal ellipsoids at 50 % probability, most hydrogen atoms omitted for clarity. Solvent has been omitted for clarity. Boron, carbon, iron, nitrogen, hydrogen, and phosphorus atoms shown with pink, black, orange, blue, white, and purple respectively.


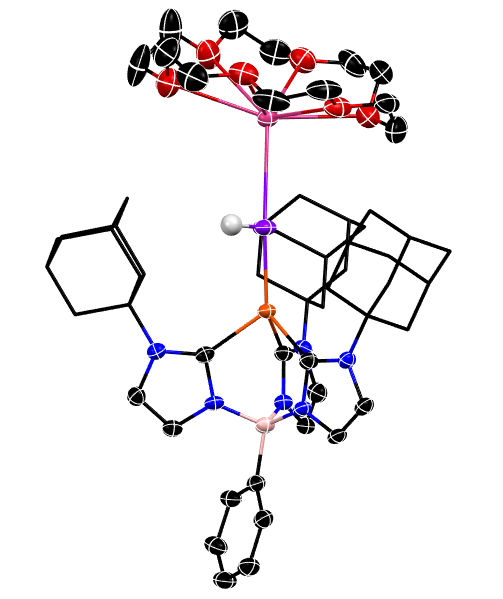


**Figure S53.** X-ray crystal structure of [K(18-crown-6)][PhB(AdIm)_3_FePH] (**3-Crown**); thermal ellipsoids at 50 % probability, most hydrogen atoms omitted for clarity. Boron, carbon, iron, nitrogen, hydrogen, phosphorus, oxygen, and potassium atoms shown with pink, black, orange, blue, white, purple, red, and magenta respectively.


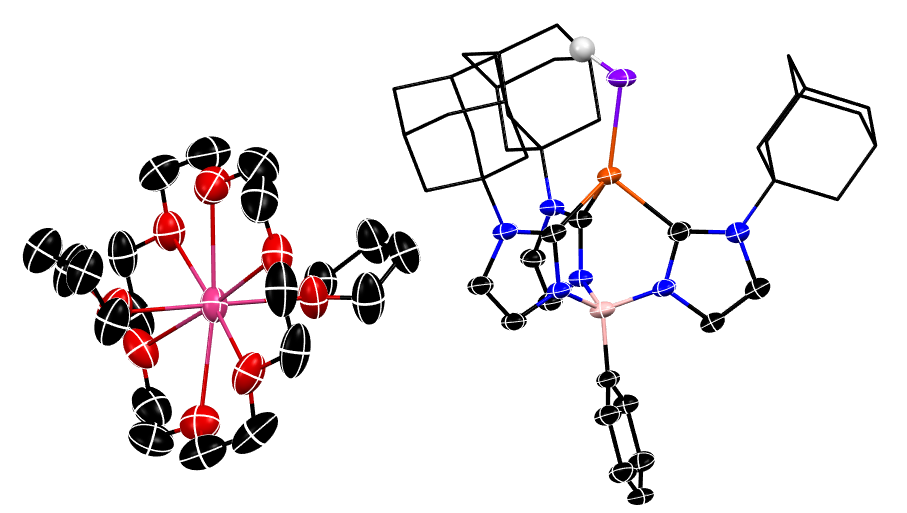


**Figure S54.** X-ray crystal structure of [K(18-crown-6)(THF)_2_][PhB(AdIm)_3_FePH]; thermal ellipsoids at 50 % probability, most hydrogen atoms omitted for clarity. Boron, carbon, iron, nitrogen, hydrogen, phosphorus, oxygen, and potassium atoms shown with pink, black, orange, blue, white, purple, red, and magenta respectively.

a)

b)
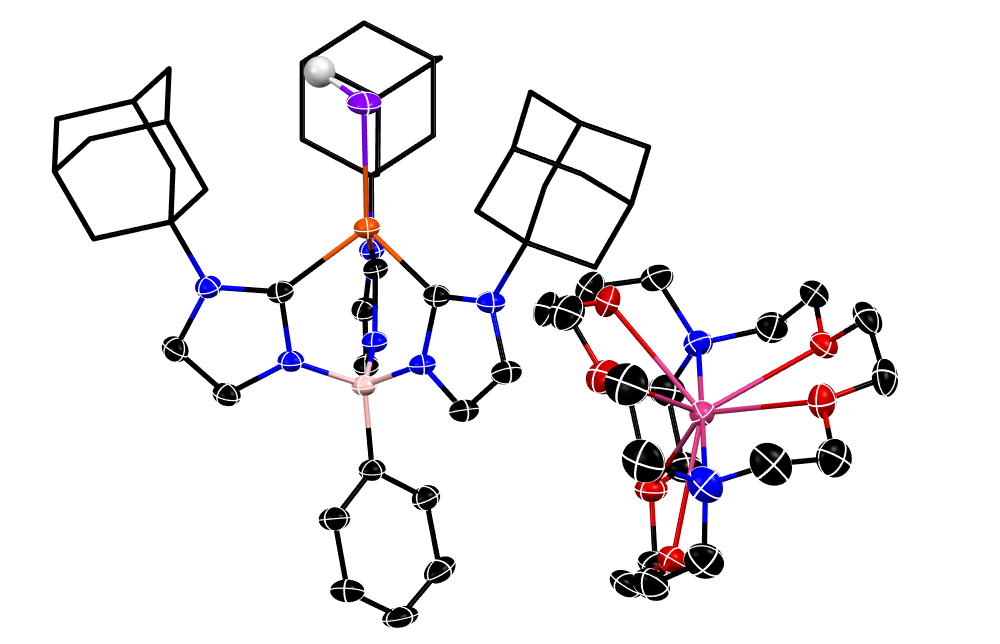


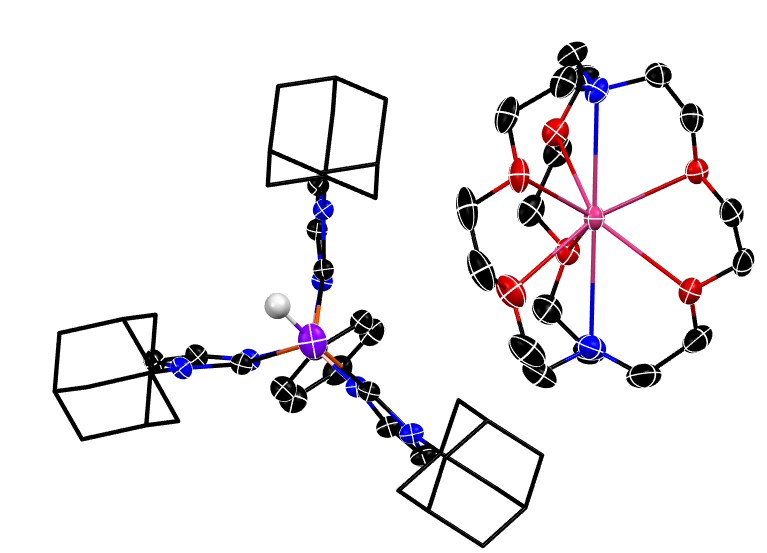


**Figure S55.** a) X-ray crystal structure of [K(2,2,2-crypt)][PhB(AdIm)_3_FePH] (**3-crypt**); thermal ellipsoids at 50 % probability, most hydrogen atoms omitted for clarity. Boron, carbon, iron, nitrogen, hydrogen, phosphorus, oxygen, and potassium atoms shown with pink, black, orange, blue, white, purple, red, and magenta respectively. b) A top-down view of X-ray crystal structure of [K(2,2,2-crypt)][PhB(AdIm)_3_FePH] (**3-crypt**).


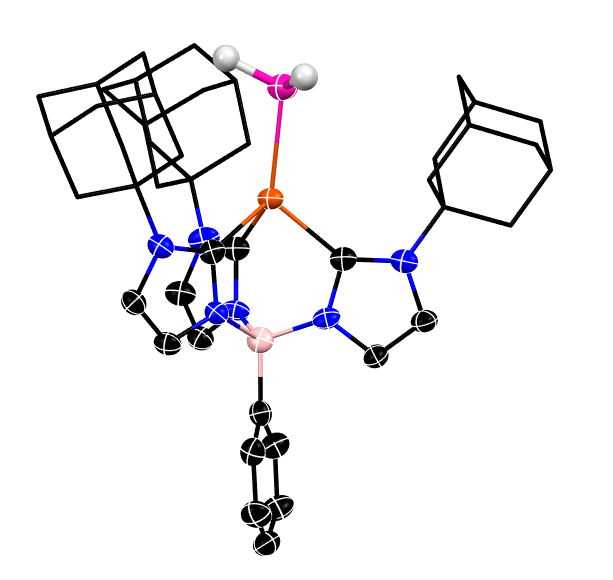


**Figure S56.** X-ray crystal structure of PhB(AdIm)_3_FeAsH_2_ (**2**); thermal ellipsoids at 50 % probability, most hydrogen atoms and solvent molecules are omitted for clarity. Boron, carbon, iron, nitrogen, hydrogen, and arsenic atoms shown with pink, black, orange, blue, white, and fuchsia respectively.

a)


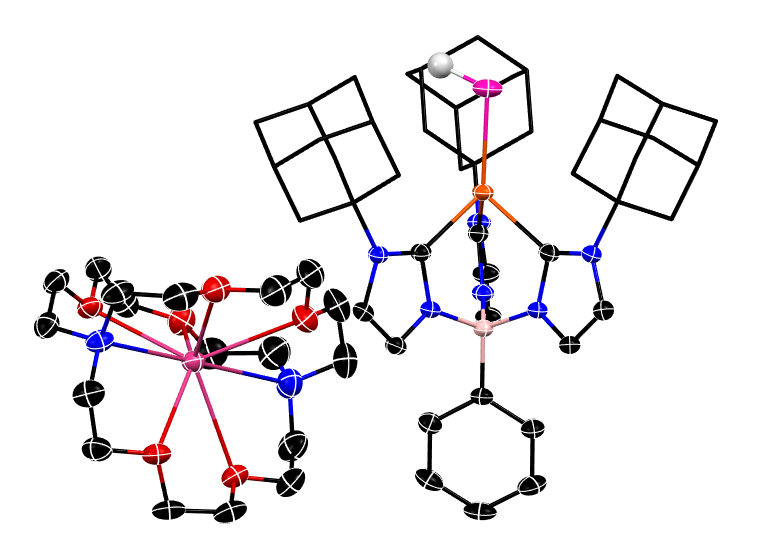


b)


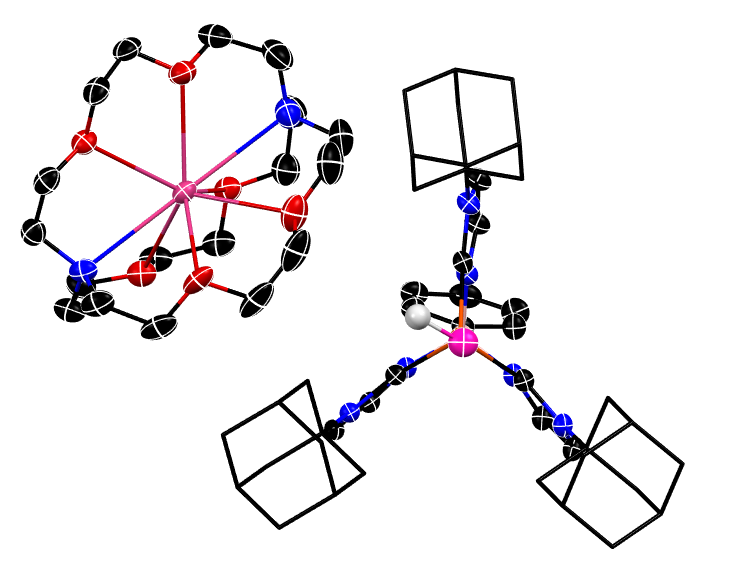


**Figure S57.** a) X-ray crystal structure of [K(2,2,2-crypt)][PhB(AdIm)_3_FeAsH] (**4-crypt**); thermal ellipsoids at 50 % probability, most hydrogen atoms omitted for clarity. Boron, carbon, iron, nitrogen, hydrogen, arsenic, oxygen, and potassium atoms shown with pink, black, orange, blue, white, fuchsia, red, and magenta respectively. b) A top-down view of X-ray crystal structure of [K(2,2,2-crypt)][PhB(AdIm)_3_FePH] (**4-crypt**).

**Figure S58.** Ball and stick representation of the molecular structure of [K(18-crown-6)][(DippNCH_2_)_2_PAsH] (**6**). Carbon, nitrogen, hydrogen, phosphorus, arsenic, oxygen, and potassium atoms shown with black, blue, white, purple, fuchsia, red, and magenta respectively.


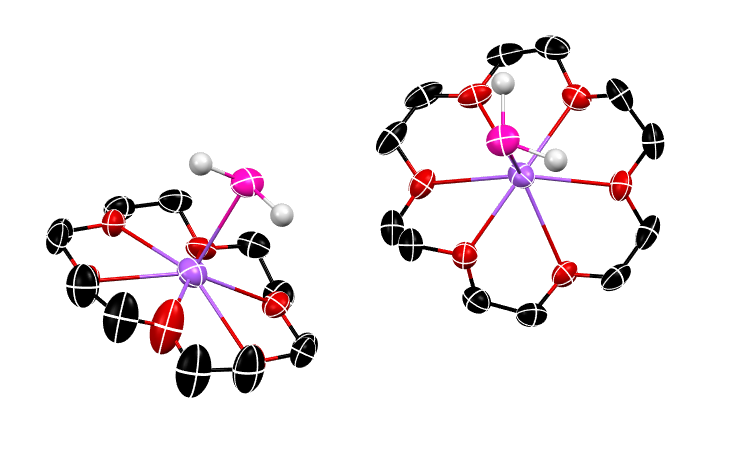


**Figure S59.** X-ray crystal structure of [Na(18-crown-6)][AsH_2_]; thermal ellipsoids at 50 % probability, most hydrogen atoms omitted for clarity. Carbon, hydrogen, arsenic, oxygen, and sodium atoms shown with black, white, fuchsia, red, and violet respectively.

**PhB(AdIm)_3_FePH_2_ (1).**

**Data collection**

The data collection was carried out using Mo Kα radiation (multilayer mirror monochromator) with a frame time of 0.7 and 7 seconds and a detector distance of 50 mm. A collection strategy was calculated and complete data to a resolution of 0.77 Å (six sets of frames) were collected with 0.5º ω and φ scans. A total of 2420 frames were collected. The total exposure time was 3.02 hours. The frames were integrated with the SAINT V8.41 package using a narrow-frame algorithm.{, 2024 #24} The integration of the data using a monoclinic unit cell yielded a total of 170555 reflections to a maximum θ angle of 26.40° (0.80 Å resolution), of which 9723 were independent (average redundancy 17.41, completeness = 100.0%, R_int_ = 5.36%, R_sig_ = 3.48%) and 8819 (90.7%) were greater than 2σ(*F*^2^). The final cell constants of a = 13.8644(6) Å, b = 17.6271(7) Å, c = 19.4292(8) Å, volume = 4741.5(3) Å^3^, are based upon the refinement of the XYZ-centroids of 9780 reflections above 20 σ(*I*) with 2.40° < 2θ < 26.30°. Data were corrected for absorption effects using the Multi-Scan method in TWINABS Bruker. The calculated minimum and maximum transmission coefficients (based on crystal size) are 0.857 and 0.964.{Krause, 2015 #25} Table S2 contains additional crystal and refinement information.

**Structure solution and refinement**

The space group P2_1_/c was determined based on intensity statistics and systematic absences. The structure was solved and refined using the SHELX suite of programs.{Sheldrick, 2015 #26;Sheldrick, 2015 #27} An intrinsic-methods solution was calculated, which provided most non-hydrogen atoms from the E-map. Full-matrix least squares / difference Fourier cycles were performed, which located the remaining non-hydrogen atoms. All non-hydrogen atoms were refined with anisotropic displacement parameters. The hydrogen atoms were placed in ideal positions and refined as riding atoms with relative isotropic displacement parameters. The final anisotropic full-matrix least-squares refinement on F2 with 614 variables against 9723 data points and 817 restraints converged at R1 = 4.43%, for the observed data and wR2 = 10.48% for all data. The goodness-of-fit on F2 was 1.06. The largest peak in the final difference electron density synthesis was 0.53 e^−^/Å^3^ and the deepest hole was −0.33 e^−^/Å^3^ with an RMS deviation of 0.065 e^−^/Å^3^. On the basis of the final model, the calculated density was 1.30 g/cm3 and F(000), 1984 e−. PH_2_ was refined as disordered over two positions; hydrogen atoms were placed and refined as riding. The difference map and linear synchronous transit calculations provided guidance on the pyramidalized PH_2_ geometry as opposed to a planar geometry. Two solvent molecules (THF) cocrystallized with the complex per formula unit. Both are disordered over two positions and were refined with restraints and constraints. The structure is nonmerohedrally twinned; details are given in Table S2.

**Table S6. Crystal data and structure refinement for PhB(AdIm)_3_FePH_2_ (1).**

Empirical formula C53 H74 B Fe N6 O2 P

Formula weight 924.81

Crystal color, shape, size yellow plate, 0.398 × 0.211 × 0.092 mm3

Temperature 173(2) K

Wavelength 0.71073 Å

Crystal system, space group Monoclinic, P2**_1_**/c

Unit cell dimensions a = 13.8644(6) Å α = 90°.

b = 17.6271(7) Å β = 93.064(2)°.

c = 19.4292(8) Å γ = 90°.

Volume 4741.5(3) Å3

Z 4

Density (calculated) 1.296 Mg/m3

Absorption coefficient 0.400 mm-1

F(000) 1984

***Data collection***

Diffractometer Venture D8, Bruker

Source Iμ3.0, Incoatec

Detector Photon III

Theta range for data collection 1.561 to 26.399°.

Index ranges -17<=h<=17, 0<=k<=22, 0<=l<=24

Reflections collected 169313

Independent reflections 9723 [Rint = 0.0931]

Observed Reflections 8819

Completeness to theta = 25.242° 100.0 %

***Solution and Refinement***

Absorption correction Semi-empirical from equivalents

Max. and min. transmission 0.745372 and 0.690346

Solution Intrinsic methods

Refinement method Full-matrix least-squares on F2

Weighting scheme w = [σ2Fo2+ AP2+ BP]-1, with

P = (Fo2+ 2 Fc2)/3, A = 0.0168, B = 6.0682

Data / restraints / parameters 9723 / 817 / 614

Goodness-of-fit on F2 1.060

Final R indices [I>2σ(I)] R1 = 0.0443, wR2 = 0.0977

R indices (all data) R1 = 0.0541, wR2 = 0.1048

Largest diff. peak and hole 0.526 and -0.330 e.Å-3

**[K(18-crown-6)][PhB(AdIm)_3_FePH]** (**3-crown**).

**Data collection**

The data collection was carried out using Mo Kα radiation (multilayer mirror monochromator) with a frame time of 2 and 50 seconds and a detector distance of 4.00 cm. A collection strategy was calculated and complete data to a resolution of 0.75 Å (eight sets of frames) were collected with 0.05º ω and φ scans. A total of 2466 frames were collected. The total exposure time was 23.44 hours. The frames were integrated with the SAINT V8.40B package using a narrow-frame algorithm.⁠{, 2024 #24} The integration of the data using an orthorhombic unit cell yielded a total of 133160 reflections to a maximum θ angle of 25.04° (0.84 Å resolution), of which 10990 were independent (average redundancy 12.12, completeness = 99.8%, R_int_ = 4.76%, R_sig_ = 1.94%) and 10409 (94.7%) were greater than 2σ(*F*^2^). The final cell constants of a = 21.6676(11) Å, b = 19.8700(11) Å, c = 14.4839(8) Å, α = 90°, β = 90°, γ = 90°, volume = 6235.8(6) Å^3^, are based upon the refinement of the XYZ-centroids of 9912 reflections above 20 σ(*I*) with 2.26° < 2θ < 24.98°. Data were corrected for absorption effects using the Multi-Scan method in SADABS 2016/2. The calculated minimum and maximum transmission coefficients (based on crystal size) are 0.945 and 0.956.{Krause, 2015 #25}⁠ Table S3 contains additional crystal and refinement information.

**Structure solution and refinement**

The space group *Pna*2_1_ (33) was determined based on intensity statistics and systematic absences. The structure was solved by XT, VERSION 2018/2 and refined with full-matrix least squares / difference Fourier cycles using SHELXL-2019/2; Z = 4 for the formula unit C_64_H_89_BFeKN_6_O_6_P.{Sheldrick, 2015 #26;Sheldrick, 2015 #27} Non-hydrogen atoms were refined with anisotropic displacement parameters. The hydrogen atoms were placed in ideal positions and refined as riding atoms with relative isotropic displacement parameters. The final anisotropic full-matrix least-squares refinement on *F*^2^ with 759 variables against 10990 data points and 1275 restraints converged at *R*_1_ = 4.64%, for the observed data and w*R*_2_ = 12.29% for all data. The goodness-of-fit on *F*^2^ was 1.07. The largest peak in the final difference electron density synthesis was 1.38 e^−^/Å^3^ and the deepest hole was −0.40 e^−^/Å^3^ with an RMS deviation of 0.068 e^−^/Å^3^. On the basis of the final model, the calculated density was 1.25 g/cm^3^ and *F*(000), 2512 e^−^. The structure was refined as a merohedral twin with a 52:47 domain ratio. Solvent toluene, disordered over two sites, is part of the structure. Disorder was also refined for part of the crown ether using restraints and constraints.

**Table S7. Crystal data and structure refinement for [K(18-crown-6)][PhB(AdIm)_3_FePH]** (**3-crown**).

Empirical formula C64 H89 B Fe K N6 O6 P

Formula weight 1175.14

Crystal color, shape, size red block, 0.148 × 0.146 × 0.116 mm3

Temperature 153(2) K

Wavelength 0.71073 Å

Crystal system, space group Orthorhombic, Pna2**_1_**

Unit cell dimensions a = 21.6676(11) Å α = 90°.

b = 19.8700(11) Å β = 90°.

c = 14.4839(8) Å γ = 90°.

Volume 6235.8(6) Å3

Z 4

Density (calculated) 1.252 Mg/m3

Absorption coefficient 0.389 mm-1

F(000) 2512

***Data collection***

Diffractometer Venture D8, Bruker

Source Iμ3.0, Incoatec

Detector Photon III

Theta range for data collection 2.050 to 25.040°.

Index ranges -24<=h<=25, -23<=k<=23, -17<=l<=17

Reflections collected 133160

Independent reflections 10990 [Rint = 0.0476]

Observed Reflections 10409

Completeness to theta = 25.040° 99.8 %

***Solution and Refinement***

Absorption correction Semi-empirical from equivalents

Max. and min. transmission 0.7452 and 0.7048

Solution Intrinsic methods

Refinement method Full-matrix least-squares on F2

Weighting scheme w = [σ2Fo2+ AP2+ BP]-1, with

P = (Fo2+ 2 Fc2)/3, A = 0.0787, B = 3.3466

Data / restraints / parameters 10990 / 1275 / 759

Goodness-of-fit on F2 1.070

Final R indices [I>2σ(I)] R1 = 0.0464, wR2 = 0.1206

R indices (all data) R1 = 0.0490, wR2 = 0.1229

Twin domain ratio for merohedral twin 0.53(2):0.47(2)

Largest diff. peak and hole 1.375 and -0.398 e.Å-3

**[K(18-crown-6)(THF)_2_][PhB(AdIm)_3_FePH]**

**Data collection**

The data collection was performed using 0.5° ω and φ scans, frame times of 2 and 60 s, and a detector distance of 40 mm. Overall, 3502 frames were collected with a total exposure time of 43.37 hours. The frames were integrated with the SAINT V8.41 package using a narrow-frame algorithm.{, 2024 #24} The integration of the data using a triclinic unit cell yielded 168738 reflections to a maximum θ angle of 27.47° (0.77 Å resolution), of which 34508 were independent (average redundancy 4.89, completeness = 99.9%, R_int_ = 3.53%, R_sig_ = 2.84%) and 31023 (89.9%) were greater than 2σ(*F*^2^). The final cell constants of a = 13.6226(8) Å, b = 15.5428(9) Å, c = 19.3700(13) Å, α = 83.814(2)°, β = 88.659(2)°, γ = 68.725(2)°, volume = 3799.0(4) Å^3^, are based upon the refinement of the XYZ-centroids of 9390 reflections above 20 σ(*I*) with 2.49° < 2θ < 27.37°. Data were corrected for absorption effects using the Multi-Scan method in SADABS 2016/2. The calculated minimum and maximum transmission coefficients (based on crystal size) are 0.886 and 0.951.⁠{Krause, 2015 #25} Additional crystal and refinement information can be found in Table S4.

**Structure solution and refinement**

The space group *P*1 (1) was determined based on intensity statistics and systematic absences. The structure was solved by SHELXT 2018/2 and refined with full-matrix least squares / difference Fourier cycles using SHELXL-2019/2; Z = 2 for the formula unit C_73_H_113_BFeKN_6_O_10_P.⁠{Sheldrick, 2015 #26;Sheldrick, 2015 #27} Non-hydrogen atoms were refined with anisotropic displacement parameters. The hydrogen atoms were placed in ideal positions and refined as riding atoms with relative isotropic displacement parameters. The final anisotropic full-matrix least-squares refinement on *F*^2^ with 1864 variables against 34508 data points and 3063 restraints converged at *R*_1_ = 4.68%, for the observed data and w*R*_2_ = 13.98% for all data. The goodness-of-fit on *F*^2^ was 1.03. The largest peak in the final difference electron density synthesis was 0.59 e^−^/Å^3^ and the deepest hole was −0.35 e^−^/Å^3^ with an RMS deviation of 0.064 e^−^/Å^3^. On the basis of the final model, the calculated density was 1.20 g/cm^3^ and *F*(000), 1476 e^−^. Disorder was refined for an adamantyl group, coordinated THF and THF solvent using restraints and constraints. The structure was refined as a two-component merohedral twin with a domain ratio of 83:17. The structure was carefully checked for missed crystallographic symmetry, ^[e.g., 5]^ none of which was found. However, the two Fe complexes present in the unit cell are approximately related by a pseudo inversion center at ½ ½ ½, while the crown moiety is not.

**Table S8. Crystal data and structure refinement for [K(18-crown-6)][PhB(AdIm)_3_FePH]**

Empirical formula C73 H113 B Fe K N6 O10 P

Formula weight 1371.42

Crystal color, shape, size red block, 0.373 × 0.241 × 0.152 mm3

Temperature 153(2) K

Wavelength 0.71073 Å

Crystal system, space group Triclinic, P1

Unit cell dimensions a = 13.6226(8) Å α = 83.814(2)°.

b = 15.5428(9) Å β = 88.659(2)°.

c = 19.3700(13) Å γ = 68.725(2)°.

Volume 3799.0(4) Å3

Z, Z’ 2, 2

Density (calculated) 1.199 Mg/m3

Absorption coefficient 0.332 mm-1

F(000) 1476

***Data collection***

Diffractometer Venture D8, Bruker

Source, detector Incoatec Iμ3.0, Photon III

Theta range for data collection 2.076 to 27.472°.

Index ranges -17<=h<=17, -20<=k<=20, -25<=l<=25

Reflections collected 168738

Independent reflections 34508 [Rint = 0.0353]

Observed Reflections 31023

Completeness to theta = 25.242° 99.9 %

***Solution and Refinement***

Absorption correction Semi-empirical from equivalents

Max. and min. transmission 0.7456 and 0.6602

Solution Intrinsic methods

Refinement method Full-matrix least-squares on F2

Weighting scheme w = [σ2Fo2+ AP2+ BP]-1, with

P = (Fo2+ 2 Fc2)/3, A = , B =

Data / restraints / parameters 34508 / 3063 / 1864

Goodness-of-fit on F2 1.027

Final R indices [I>2σ(I)] R1 = 0.0468, wR2 = 0.1337

R indices (all data) R1 = 0.0526, wR2 = 0.1398

Absolute structure parameter 0.170(10), merohedral twin

Largest diff. peak and hole 0.591 and -0.349 e.Å-3

**[K(2,2,2-crypt)][PhB(AdIm)_3_FePH] (3-crypt).**

**Data collection**

The data collection was carried out using Mo Kα radiation (multilayer mirror monochromator) with a frame time of 30 and 1 seconds and a detector distance of 4.00 cm. A collection strategy was calculated and complete data to a resolution of 0.75 Å (seven sets of frames) were collected with 0.5º ω and φ scans. A total of 3004 frames were collected. The total exposure time was 17.53 hours. The frames were integrated with the SAINT V8.41 package using a narrow-frame algorithm.{, 2024 #24} The integration of the data using a monoclinic unit cell yielded a total of 296341 reflections to a maximum θ angle of 28.29° (0.75 Å resolution), of which 17835 were independent (average redundancy 16.62, completeness = 99.9%, R_int_ = 3.96%, R_sig_ = 1.44%) and 15347 (86.0%) were greater than 2σ(*F*^2^). The final cell constants of a = 16.4587(4) Å, b = 17.7400(4) Å, c = 25.3434(6) Å, α = 90°, β = 103.7750(10)°, γ = 90°, volume = 7186.9(3) Å^3^, are based upon the refinement of the XYZ-centroids of 9985 reflections above 20 σ(*I*) with 2.44° < 2θ < 28.21°. Data were corrected for absorption effects using the Multi-Scan method in SADABS 2016/2. The calculated minimum and maximum transmission coefficients (based on crystal size) are 0.877 and 0.95.{Krause, 2015 #25} Table S5 contains additional crystal and refinement information.

**Structure solution and refinement**

The space group *P*2_1_/*n* (14) was determined based on intensity statistics and systematic absences. The structure was solved by SHELXT 2018/2 and refined with full-matrix least squares / difference Fourier cycles using SHELXL-2019/2; Z = 4 for the formula unit C_73_H_113_BFeKN_8_O_8.50_P.{Sheldrick, 2015 #26;Sheldrick, 2015 #27} Non-hydrogen atoms were refined with anisotropic displacement parameters. The hydrogen atoms were placed in ideal positions and refined as riding atoms with relative isotropic displacement parameters with exception of H1p which was found in in the difference map and refined as a riding atom. The final anisotropic full-matrix least-squares refinement on *F*^2^ with 853 variables against 17835 data points and 56 restraints converged at *R*_1_ = 4.02%, for the observed data and w*R*_2_ = 11.87% for all data. The goodness-of-fit on *F*^2^ was 1.03. The largest peak in the final difference electron density synthesis was 0.68 e^−^/Å^3^ and the deepest hole was −0.68 e^−^/Å^3^ with an RMS deviation of 0.051 e^−^/Å^3^. On the basis of the final model, the calculated density was 1.27 g/cm^3^ and *F*(000), 2960 e^−^. Solvent molecules are included in the structure, one of which is disordered over a special position.

**Table S9. Crystal data and structure refinement for [K(2,2,2-crypt)][PhB(AdIm)_3_FePH] (3-crypt).**

Empirical formula C73 H113 B Fe K N8 O8.50 P

Formula weight 1375.44

Crystal color, shape, size red block, 0.386 × 0.153 × 0.144 mm3

Temperature 153(2) K

Wavelength 0.71073 Å

Crystal system, space group Monoclinic, P2**_1_**/n

Unit cell dimensions a = 16.4587(4) Å α = 90°.

b = 17.7400(4) Å β = 103.7750(10)°.

c = 25.3434(6) Å γ = 90°.

Volume 7186.9(3) Å3

Z 4

Density (calculated) 1.271 Mg/m3

Absorption coefficient 0.351 mm-1

F(000) 2960

***Data collection***

Diffractometer Venture D8, Bruker

Source Iμ3.0, Incoatec

Detector Photon III

Theta range for data collection 2.162 to 28.288°.

Index ranges -21<=h<=21, -23<=k<=23, -33<=l<=33

Reflections collected 296341

Independent reflections 17835 [Rint = 0.0396]

Observed Reflections 15347

Completeness to theta = 25.242° 99.9 %

***Solution and Refinement***

Absorption correction Semi-empirical from equivalents

Max. and min. transmission 0.7457 and 0.7204

Solution Intrinsic methods

Refinement method Full-matrix least-squares on F2

Weighting scheme w = [σ2Fo2+ AP2+ BP]-1, with

P = (Fo2+ 2 Fc2)/3, A = 0.0623, B = 3.5353

Data / restraints / parameters 17835 / 56 / 853

Goodness-of-fit on F2 1.034

Final R indices [I>2σ(I)] R1 = 0.0402, wR2 = 0.1128

R indices (all data) R1 = 0.0472, wR2 = 0.1187

Largest diff. peak and hole 0.680 and -0.684 e.Å-3

**PhB(AdIm)_3_FeAsH_2_ (2)**

**Data collection**

The data collection was performed using 0.5° ω and φ scans, frame times of 120 and 5 s, and a detector distance of 40 mm. Overall, 2793 frames were collected with a total exposure time of 70.10 hours. The frames were integrated with the SAINT V8.41 package using a narrow-frame algorithm.{, 2024 #24} The integration of the data using a monoclinic unit cell yielded 85425 reflections to a maximum θ angle of 25.12° (0.84 Å resolution), of which 8384 were independent (average redundancy 10.19, completeness = 99.3%, R_int_ = 17.59%, R_sig_ = 10.85%) and 6323 (75.4%) were greater than 2σ(*F*^2^). The final cell constants of a = 13.8714(6) Å, b = 17.6014(8) Å, c = 19.4029(9) Å, α = 90°, β = 93.0743(14)°, γ = 90°, volume = 4730.5(4) Å^3^, are based upon the refinement of the XYZ-centroids of 9871 reflections above 20 σ(*I*) with 2.40° < 2θ < 24.97°. Data were corrected for absorption effects using the Multi-Scan method in TWINABS Bruker. The calculated minimum and maximum transmission coefficients (based on crystal size) are 0.891 and 0.923.{Krause, 2015 #25} Additional crystal and refinement information can be found in the Table S6.

**Structure solution and refinement**

The space group *P*2_1_/*c* (# 14) was determined based on intensity statistics and systematic absences. The structure was solved by XT, VERSION 2018/2 and refined with full-matrix least squares / difference Fourier cycles using SHELXL-2019/2; Z = 4 for the formula unit C_53_H_73.18_As_0.59_BCl_0.41_FeN_6_O_2_.{Sheldrick, 2015 #26;Sheldrick, 2015 #27} Non-hydrogen atoms were refined with anisotropic displacement parameters. The hydrogen atoms were placed in ideal positions and refined as riding atoms with relative isotropic displacement parameters. The final anisotropic full-matrix least-squares refinement on *F*^2^ with 614 variables against 8384 data points and 812 restraints converged at *R*_1_ = 8.02%, for the observed data and w*R*_2_ = 21.85% for all data. The goodness-of-fit on *F*^2^ was 1.06. The largest peak in the final difference electron density synthesis was 0.78 e^−^/Å^3^ and the deepest hole was −0.52 e^−^/Å^3^ with an RMS deviation of 0.118 e^−^/Å^3^. On the basis of the final model, the calculated density was 1.34 g/cm^3^ and *F*(000), 2027 e^−^. The structure was refined as a 2-component non-merohedral twin (180.0° rotation about reciprocal axis -0.033 0 1 and real axis 0 0 1; twin law to convert hkl from first to second domain by the rows -0.999 0.001 -0.065, -0.001 -1.000 0.003, -0.018 -0.009 0.999; twin domain ratio 54:46). Disorder was refined for ligand As/Cl and co-crystallized THF solvent molecules using restraints and constraints. Hydrogen atoms on As1 were based on computational results. PhB(AdIm)_3_FeAsH_2_ is isostructural to the P analog.

**Table S10. Crystal data and structure refinement for PhB(AdIm)_3_FeAsH_2_ (2)**

Empirical formula C53 H73.18 As0.59 B Cl0.41 Fe N6 O2

Formula weight 951.80

Crystal color, shape, size yellow block, 0.148 × 0.137 × 0.102 mm3

Temperature 153(2) K

Wavelength 0.71073 Å

Crystal system, space group Monoclinic, P2**_1_**/c

Unit cell dimensions a = 13.8714(6) Å α = 90°.

b = 17.6014(8) Å β = 93.0743(14)°.

c = 19.4029(9) Å γ = 90°.

Volume 4730.5(4) Å3

Z 4

Density (calculated) 1.336 Mg/m3

Absorption coefficient 0.801 mm-1

F(000) 2027

***Data collection***

Diffractometer Venture D8, Bruker

Source, detector Incoatec Iμ3.0, Photon III

Theta range for data collection 2.184 to 25.116°.

Index ranges -16<=h<=16, 0<=k<=20, 0<=l<=23

Reflections collected 85425

Independent reflections 8384 [Rint = 0.1759]

Observed Reflections 6323

Completeness to theta = 25.116° 99.3 %

***Solution and Refinement***

Absorption correction Semi-empirical from equivalents

Max. and min. transmission 0.74516 and 0.36776

Solution Intrinsic methods

Refinement method Full-matrix least-squares on F2

Weighting scheme w = [σ2Fo2+ AP2+ BP]-1, with

P = (Fo2+ 2 Fc2)/3, A = , B =

Data / restraints / parameters 8384 / 812 / 614

Goodness-of-fit on F2 1.063

Final R indices [I>2σ(I)] R1 = 0.0802, wR2 = 0.1847

R indices (all data) R1 = 0.1219, wR2 = 0.2185

Largest diff. peak and hole 0.779 and -0.516 e.Å-3

Twin details nonmerohedral, 180.0 about d.s. 0 0 1, ratio 54:46

**[K(2,2,2-crypt)][PhB(AdIm)_3_FeAsH] (4-crypt).**

**Data collection**

The data collection was performed using 0.5° ω and φ scans, frame times of 25 and 1 s, and a detector distance of 40 mm. Overall, 3004 frames were collected with a total exposure time of 14.65 hours. The frames were integrated with the SAINT V8.41 package using a narrow-frame algorithm.{, 2024 #24} The integration of the data using a monoclinic unit cell yielded 271183 reflections to a maximum θ angle of 26.89° (0.79 Å resolution), of which 15531 were independent (average redundancy 17.46, completeness = 99.9%, R_int_ = 3.60%, R_sig_ = 1.34%) and 13889 (89.4%) were greater than 2σ(*F*^2^). The final cell constants of a = 16.4945(5) Å, b = 17.7267(5) Å, c = 25.3741(7) Å, α = 90°, β = 103.7140(10)°, γ = 90°, volume = 7207.7(4) Å^3^, are based upon the refinement of the XYZ-centroids of 9211 reflections above 20 σ(*I*) with 2.54° < 2θ < 26.87°. Data were corrected for absorption effects using the Multi-Scan method in SADABS 2016/2. The calculated minimum and maximum transmission coefficients (based on crystal size) are 0.750 and 0.824.{Krause, 2015 #25} Additional crystal and refinement information can be found in Table S7.

**Structure solution and refinement**

The space group *P*2_1_/*n* (*n*=14) was determined based on intensity statistics and systematic absences. The structure was solved by SHELXT 2018/2 and refined with full-matrix least squares / difference Fourier cycles using SHELXL-2019/2; Z = 4 for the formula unit C_73_H_113_AsBFeKN_8_O_8.50_.⁠{Sheldrick, 2015 #26;Sheldrick, 2015 #27} Non-hydrogen atoms were refined with anisotropic displacement parameters. The hydrogen atoms were placed in ideal positions and refined as riding atoms with relative isotropic displacement parameters. The final anisotropic full-matrix least-squares refinement on *F*^2^ with 853 variables against 15531 data points and 964 restraints converged at *R*_1_ = 3.85%, for the observed data and w*R*_2_ = 11.51% for all data. The goodness-of-fit on *F*^2^ was 1.03. The largest peak in the final difference electron density synthesis was 0.86 e^−^/Å^3^ and the deepest hole was −0.89 e^−^/Å^3^ with an RMS deviation of 0.056 e^−^/Å^3^. On the basis of the final model, the calculated density was 1.31 g/cm^3^ and *F*(000), 3032 e^−^. Solvent molecules are included in the structure, one of which is disordered over a special position. The structure is isostructural to the P analog [K(2,2,2-crypt)][PhB(AdIm)_3_FePH] (3**-Crypt**).

**Table S11. Crystal data and structure refinement for [K(2,2,2-crypt)][PhB(AdIm)_3_FeAsH] (4-crypt).**

Empirical formula C73 H113 As B Fe K N8 O8.50

Formula weight 1419.39

Crystal color, shape, size red prism, 0.390 × 0.352 × 0.258 mm3

Temperature 153(2) K

Wavelength 0.71073 Å

Crystal system, space group Monoclinic, P2**_1_**/n

Unit cell dimensions a = 16.4945(5) Å α = 90°.

b = 17.7267(5) Å β = 103.7140(10)°.

c = 25.3741(7) Å γ = 90°.

Volume 7207.7(4) Å3

Z 4

Density (calculated) 1.308 Mg/m3

Absorption coefficient 0.783 mm-1

F(000) 3032

***Data collection***

Diffractometer Venture D8, Bruker

Source, detector Incoatec Iμ3.0, Photon III

Theta range for data collection 2.161 to 26.889°.

Index ranges -20<=h<=20, -22<=k<=22, -32<=l<=32

Reflections collected 271183

Independent reflections 15531 [Rint = 0.0360]

Observed Reflections 13889

Completeness to theta = 25.242° 99.9 %

***Solution and Refinement***

Absorption correction Semi-empirical from equivalents

Max. and min. transmission 0.7455 and 0.6983

Solution Intrinsic methods

Refinement method Full-matrix least-squares on F2

Weighting scheme w = [σ2Fo2+ AP2+ BP]-1, with

P = (Fo2+ 2 Fc2)/3, A = 0.0644, B = 4.8054

Data / restraints / parameters 15531 / 964 / 853

Goodness-of-fit on F2 1.027

Final R indices [I>2σ(I)] R1 = 0.0385, wR2 = 0.1111

R indices (all data) R1 = 0.0429, wR2 = 0.1151

Largest diff. peak and hole 0.856 and -0.886 e.Å-3

**[K(18-crown-6)][(DippNCH_2_)_2_PAsH] (5).**

**Data collection**

The screening data collection was carried out using Mo Kα radiation (graphite monochromator) with a frame time of 10 seconds and a detector distance of 5.00 cm and performing a 180° φ scans until reflections ceased due to decomposition.

The integration of the data using a triclinic unit cell yielded 18466 reflections to a maximum θ angle of 18.90° (1.10 Å resolution), of which 9930 were independent (average redundancy 1.86, completeness = 92.2%, R_int_ = 37.99%, R_sig_ = 54.53%) and 2399 (24.2%) were greater than 2σ(*F*^2^). The final cell constants of a = 11.71(3) Å, b = 25.50(6) Å, c = 26.33(6) Å, α = 114.37(5)°, β = 99.76(4)°, γ = 100.01(4)°, volume = 6792(25) Å^3^, are based upon the refinement of the XYZ-centroids of 180 reflections above 20 σ(*I*) with 2.24° < 2θ < 10.99°. Data were corrected for absorption effects using the Multi-Scan method in SADABS 2016/2. The calculated minimum and maximum transmission coefficients (based on crystal size) are 0.895 and 0.928.{Krause, 2015 #25} Additional crystal and refinement information can be found in the tables. Please refer to Table S8 for additional information.

**Structure solution and refinement**

The space group P-1 (# 2) was determined based on intensity statistics and systematic absences. The structure was solved and refined using the SHELX suite of programs.^{Sheldrick, 2015 #26;Sheldrick, 2015 #27}^ An intrinsic-methods solution was calculated, which provided most non-hydrogen atoms from the E-map. Full-matrix least squares / difference Fourier cycles were performed, which located the remaining non-hydrogen atoms. If possible non-hydrogen atoms were refined with anisotropic displacement parameters, including K, As, and P, and the hydrogen atoms were placed based on geometric considerations.

Due to decomposition during the experiment, data are insufficient to perform a full refinement and obtain a publication-ready result. However, connectivity was established and is reasonably reliable. Details of the structure such as disorder, co-crystallized solvents, twinning, may not be included in the refinement. Decomposition of the crystal is attributed to a number of factors but is not limited to inherit instability and climate effects during measurement, namely summer humidity.

**Table S12. Crystal data and structure refinement for [K(18-crown-6)][(DippNCH_2_)_2_PAsH] (5).**

Empirical formula C38 H63 As K N2 O6 P

Formula weight 788.89

Crystal color, shape, size plate colorless, 0.124 × 0.108 × 0.083 mm3

Temperature 153(2) K

Wavelength 0.71073 Å

Crystal system, space group Triclinic, P-1

Unit cell dimensions a = 11.71(3) Å α = 114.37(5)°.

b = 25.50(6) Å β = 99.76(4)°.

c = 26.33(6) Å γ = 100.01(4)°.

Volume 6792(25) Å3

Z, Z’ 6, 3

Density (calculated) 1.157 Mg/m3

Absorption coefficient 0.918 mm-1

F(000) 2520

***Data collection (incomplete screening data)***

Diffractometer Venture D8, Bruker

Source, detector Incoatec Iμ3.0, Photon III

Theta range for data collection 1.871 to 18.902°.

Index ranges -10<=h<=10, -23<=k<=23, -23<=l<=23

Reflections collected 18466

Independent reflections 9930 [Rint = 0.3799]

Observed Reflections 2399

Completeness to theta = 18.902° 92.2 %

***Solution and Refinement***

Absorption correction Semi-empirical from equivalents

Max. and min. transmission 0.7443 and 0.5730

Solution Intrinsic methods

Refinement method Full-matrix least-squares on F2

**[Na(18-crown-6)][AsH_2_]**

**Data Collection**

The data collection was performed using 0.5° ω and φ scans, frame times of 45 and 1 s, and a detector distance of 40 mm. Overall, 2284 frames were collected with a total exposure time of 17.17 hours. The frames were integrated with the SAINT V8.41 package using a narrow-frame algorithm.⁠{, 2024 #24} The integration of the data using a monoclinic unit cell yielded 79036 reflections to a maximum θ angle of 26.42° (0.80 Å resolution), of which 7253 were independent (average redundancy 10.90, completeness = 99.8%, R_int_ = 5.12%, R_sig_ = 2.48%) and 6155 (84.9%) were greater than 2σ(*F*^2^). The final cell constants of a = 20.8129(7) Å, b = 8.3237(4) Å, c = 22.0262(9) Å, α = 90°, β = 112.2680(10)°, γ = 90°, volume = 3531.2(3) Å^3^, are based upon the refinement of the XYZ-centroids of 1858 reflections above 20 σ(*I*) with 2.64° < 2θ < 26.31°. Data were corrected for absorption effects using the Multi-Scan method in SADABS 2016/2. The calculated minimum and maximum transmission coefficients (based on crystal size) are 0.669 and 0.811.⁠{Krause, 2015 #25} Additional crystal and refinement information can be found in Table S9.

**Structure solution and refinement**

The space group *P*2_1_/*n* (14) was determined based on intensity statistics and systematic absences. The structure was solved by SHELXT 2018/2 and refined with full-matrix least squares / difference Fourier cycles using SHELXL-2019/2; Z = 8, Z’ = 2 for the formula unit C_12_H_26_AsNaO_6_.⁠{Sheldrick, 2015 #26;Sheldrick, 2015 #27} Non-hydrogen atoms were refined with anisotropic displacement parameters. The hydrogen atoms were placed in ideal positions and refined as riding atoms with relative isotropic displacement parameters. The final anisotropic full-matrix least-squares refinement on *F*^2^ with 474 variables against 7253 data points and 3697 restraints converged at *R*_1_ = 7.38%, for the observed data and w*R*_2_ = 20.46% for all data. The goodness-of-fit on *F*^2^ was 1.04. The largest peak in the final difference electron density synthesis was 2.08 e^−^/Å^3^ and the deepest hole was −1.24 e^−^/Å^3^ with an RMS deviation of 0.097 e^−^/Å^3^. On the basis of the final model, the calculated density was 1.37 g/cm^3^ and *F*(000), 1520 e^−^. Disorder was refined for the crowns and As1B using restraints and constraints. Two formula units are in the asymmetric unit.

**Table S13. Crystal data and structure refinement for [Na(18-crown-6)][AsH_2_].**

Empirical formula C12 H26 As Na O6

Formula weight 364.24

Crystal color, shape, size colorless block, 0.222 × 0.140 × 0.111 mm3

Temperature 173(2) K

Wavelength 0.71073 Å

Crystal system, space group Monoclinic, P2**_1_**/n

Unit cell dimensions a = 20.8129(7) Å α = 90°.

b = 8.3237(4) Å β = 112.2680(10)°.

c = 22.0262(9) Å γ = 90°.

Volume 3531.2(3) Å3

Z, Z’ 8, 2

Density (calculated) 1.370 Mg/m3

Absorption coefficient 1.968 mm-1

F(000) 1520

***Data collection***

Diffractometer Venture D8, Bruker

Source, detector Incoatec Iμ3.0, Photon III

Theta range for data collection 1.998 to 26.421°.

Index ranges -23<=h<=26, -10<=k<=10, -27<=l<=27

Reflections collected 79036

Independent reflections 7253 [Rint = 0.0512]

Observed Reflections 6155

Completeness to theta = 25.242° 99.8 %

***Solution and Refinement***

Absorption correction Semi-empirical from equivalents

Max. and min. transmission 0.7454 and 0.6274

Solution Intrinsic methods

Refinement method Full-matrix least-squares on F2

Weighting scheme w = [σ2Fo2+ AP2+ BP]-1, with

P = (Fo2+ 2 Fc2)/3, A = 0.0978, B = 9.2025

Data / restraints / parameters 7253 / 3697 / 474

Goodness-of-fit on F2 1.037

Final R indices [I>2σ(I)] R1 = 0.0738, wR2 = 0.1953

R indices (all data) R1 = 0.0833, wR2 = 0.2046

Largest diff. peak and hole 2.077 and -1.235 e.Å-3

# References

[75] D. Ergöçmen, J. M. Goicoechea, *Angew. Chem. Int. Ed.* **2021**, *60*, 25286-25289.

[76] O. Puntigam, I. Hajdók, M. Nieger, M. Niemeyer, S. Strobel, D. Gudat, *Z. Anorg. Allg. Chim.* **2011**, *637*, 988-994.

[77] M. D. Taylor, L. R. Grant, *J. Chem. Ed.* **1955**, *32*, 39.

[78] L. Lochmann, J. Trekoval, *J. Organomet. Chem.* **1987**, *326*, 1-7.

[79] J. A. Valdez-Moreira, D. M. Beagan, H. Yang, J. Telser, B. M. Hoffman, M. Pink, V. Carta, J. M. Smith, *ACS Cent. Sci.* **2021**, *7*, 1751-1755.

[80] S. Kossmann, F. Neese, *Chem. Phys. Lett.* **2009**, *481*.

[81] F. Neese, *J. Comput. Chem.* **2003**, *24*, 1740-1747.

[82] F. Neese, *WIREs Comp. Mol. Sci.* **2022**, *5*, e1606.

[83] F. Neese, *J. Comp. Chem.* **2022**, *44*, 381-396.

[84] Y. Guo, K. Sivalingam, F. Neese, *J. Chem. Phys.* **2021**, *154*, 214111.

[85] C. Kollmar, K. Sivalingam, B. Helmich-Paris, C. Angeli, F. Neese, *J. Comput. Chem.* **2019**, *40*, 1463-1470.

[86] C. Kollmar, K. Sivalingam, F. Neese, *J. Chem. Phys.* **2021**, *155*, 234104.

[87] M. Ugandi, M. Roemelt, *Int. J. Quantum Chem.* **2023**, *123*, e27045.

[88] F. Neese, *Inorg. Chim. Acta* **2002**, *337*, 181-192.
