# Supplementary material for: High Spin Iron–Phosphinidene and Arsinidene Complexes With Attenuated Metal–Ligand Multiple Bond Character
Source: Angew Chem Int Ed Engl. 2026 Mar 19;65(18):e23239. doi: 10.1002/anie.202523239 (PMC13110769; doi:10.1002/anie.202523239)

checkCIF/PLATON report

Structure factors have been supplied for datablock(s) 25088

THIS REPORT IS FOR GUIDANCE ONLY. IF USED AS PART OF A REVIEW PROCEDURE FOR PUBLICATION, IT SHOULD NOT REPLACE THE EXPERTISE OF AN EXPERIENCED CRYSTALLOGRAPHIC REFEREE.

No syntax errors found. CIF dictionary Interpreting this report

Datablock: 25088

```
Bond precision:   As-Na = 0.0017 A         Wavelength=0.71073

Cell:             a=20.8129(7)     b=8.3237(4)         c=22.0262(9)
                  alpha=90         beta=112.268(1)     gamma=90
Temperature:      173 K

                 Calculated                    Reported
Volume           3531.3(3)                     3531.2(3)
Space group      P 21/n                        P 21/n
Hall group       -P 2yn                        -P 2yn
Moiety formula   C12 H26 As Na O6              ?
Sum formula      C12 H26 As Na O6              C12 H26 As Na O6
Mr               364.24                        364.24
Dx,g cm-3        1.370                         1.370
Z                8                             8
Mu (mm-1)        1.968                         1.968
F000             1520.0                        1520.0
F000'            1521.29
h,k,lmax         26,10,27                      26,10,27
Nref             7278                          7253
Tmin,Tmax        0.726,0.804                   0.627,0.745
Tmin'            0.640

Correction method= # Reported T Limits: Tmin=0.627 Tmax=0.745
AbsCorr = MULTI-SCAN

Data completeness= 0.997            Theta(max)= 26.421

                                                    wR2(reflections)=
R(reflections)= 0.0738( 6155)                       0.2046( 7253)

S = 1.037                     Npar= 474
```

The following ALERTS were generated. Each ALERT has the format
test-name_ALERT_alert-type_alert-level.
Click on the hyperlinks for more details of the test.

Alert level C

```
PLAT420_ALERT_2_C D-H Bond Without Acceptor  As1A     --H1A      .     Please Check
PLAT420_ALERT_2_C D-H Bond Without Acceptor  As1A     --H1B      .     Please Check
PLAT420_ALERT_2_C D-H Bond Without Acceptor  As1B     --H1C      .     Please Check
PLAT420_ALERT_2_C D-H Bond Without Acceptor  As1B     --H1D      .     Please Check
PLAT906_ALERT_3_C Large K Value in the Analysis of Variance ......      3.204 Check
PLAT911_ALERT_3_C Missing FCF Refl Between Thmin & STh/L=    0.600          8 Report
                 2   0   0,    4   0   0,   -7   0   1,  -5   0   1,   -3   0   1, -12   0   2,
                -6   0   2,   -7   0   3,
PLAT971_ALERT_2_C Check Calcd Resid. Dens.  0.75Ang From As1B           2.07 eA-3
PLAT977_ALERT_2_C Check Negative Difference Density on H1A       .      -0.40 eA-3
PLAT977_ALERT_2_C Check Negative Difference Density on H1B       .      -0.38 eA-3
PLAT977_ALERT_2_C Check Negative Difference Density on H7BB      .      -0.36 eA-3
```

Alert level G

```
PLAT002_ALERT_2_G Number of Distance or Angle Restraints on AtSite         72 Note
PLAT003_ALERT_2_G Number of Uiso or U(i,j)  Restrained non-H-Atoms         72 Report
PLAT083_ALERT_2_G SHELXL Second Parameter in WGHT  Unusually Large       9.20 Why ?
PLAT171_ALERT_4_G The CIF-Embedded .res File Contains EADP Records         37 Report
PLAT175_ALERT_4_G The CIF-Embedded .res File Contains SAME Records         14 Report
PLAT176_ALERT_4_G The CIF-Embedded .res File Contains SADI Records          4 Report
PLAT178_ALERT_4_G The CIF-Embedded .res File Contains SIMU Records          2 Report
PLAT187_ALERT_4_G The CIF-Embedded .res File Contains RIGU Records          1 Report
PLAT191_ALERT_3_G A Non-default SADI Restraint Value has been used     0.0100 Report
PLAT191_ALERT_3_G A Non-default SADI Restraint Value has been used     0.0100 Report
PLAT191_ALERT_3_G A Non-default SADI Restraint Value has been used     0.0100 Report
PLAT191_ALERT_3_G A Non-default SADI Restraint Value has been used     0.0100 Report
PLAT301_ALERT_3_G Main Residue  Disorder ..............(Resd    1)        95% Note
PLAT302_ALERT_4_G Anion/Solvent/Minor-Residue Disorder (Resd    2)        90% Note
PLAT398_ALERT_2_G Deviating  C-O-C   Angle From 120 for O5B      .      109.8 Degree
PLAT398_ALERT_2_G Deviating  C-O-C   Angle From 120 for O1D      .      109.8 Degree
PLAT411_ALERT_2_G Short Inter H...H Contact  H1B      ..H2DB     .       2.13 Ang.
                                     1/2+x,1/2-y,1/2+z  =      4_666 Check
PLAT720_ALERT_4_G Number of Unusual/Non-Standard Labels ..........         72 Note
              H1AA    H1AB    H2AA    H2AB    H3AA    H3AB    H4AA    H4AB
              H5AA    H5AB    H6AA    H6AB    H7AA    H7AB    H8AA    H8AB
              H9AA    H9AB    H1EA    H1EB    H2EA    H2EB    H3EA    H3EB
              H4EA    H4EB    H5EA    H5EB    H6EA    H6EB    H7EA    H7EB
              H8EA    H8EB    H9EA    H9EB    H1BA    H1BB    H2BA    H2BB
              H3BA    H3BB    H4BA    H4BB    H5BA    H5BB    H6BA    H6BB
              H7BA    H7BB    H8BA    H8BB    H9BA    H9BB    H1DA    H1DB
              H2DA    H2DB    H3DA    H3DB    H4DA    H4DB    H5DA    H5DB
              H6DA    H6DB    H7DA    H7DB    H8DA    H8DB    H9DA    H9DB
PLAT721_ALERT_1_G Bond    Calc     1.01000, Rep     0.99000 Dev...       0.02 Ang.
              C1A      -H1AB           1_555   1_555 ........     #   19 Check
PLAT722_ALERT_1_G Angle   Calc      111.00, Rep      109.80 Dev...       1.20 Degree
              O3A     -C4A     -H4AB       1_555  1_555  1_555     #   67 Check
PLAT764_ALERT_4_G Overcomplete CIF Bond List Detected (Rep/Expd) .       1.12 Ratio
PLAT811_ALERT_5_G No ADDSYM Analysis: Too Many Excluded Atoms ....          ! Info
PLAT860_ALERT_3_G Number of Least-Squares Restraints ............        3697 Note
```

```
PLAT910_ALERT_3_G Missing FCF Reflection(s) Below Theta(Min)[Deg]=       2.00 Note
               -1  0  1,   1  0  1,
PLAT912_ALERT_4_G Missing # of FCF Reflections Above STh/L=  0.600         15 Note
PLAT969_ALERT_5_G The 'Henn et al.' R-Factor-gap value ..........      6.792 Note
              Predicted wR2: Based on SigI**2  3.01 or SHELX Weight 19.73
```

```
  0 ALERT level A = Most likely a serious problem - resolve or explain
  0 ALERT level B = A potentially serious problem, consider carefully
 10 ALERT level C = Check. Ensure it is not caused by an omission or oversight
 26 ALERT level G = General information/check it is not something unexpected

  2 ALERT type 1 CIF construction/syntax error, inconsistent or missing data
 14 ALERT type 2 Indicator that the structure model may be wrong or deficient
  9 ALERT type 3 Indicator that the structure quality may be low
  9 ALERT type 4 Improvement, methodology, query or suggestion
  2 ALERT type 5 Informative message, check
```

It is advisable to attempt to resolve as many as possible of the alerts in all categories. Often the minor alerts point to easily fixed oversights, errors and omissions in your CIF or refinement strategy, so attention to these fine details can be worthwhile. In order to resolve some of the more serious problems it may be necessary to carry out additional measurements or structure refinements. However, the purpose of your study may justify the reported deviations and the more serious of these should normally be commented upon in the discussion or experimental section of a paper or in the "special_details" fields of the CIF. checkCIF was carefully designed to identify outliers and unusual parameters, but every test has its limitations and alerts that are not important in a particular case may appear. Conversely, the absence of alerts does not guarantee there are no aspects of the results needing attention. It is up to the individual to critically assess their own results and, if necessary, seek expert advice.

Publication of your CIF in IUCr journals

A basic structural check has been run on your CIF. These basic checks will be run on all CIFs submitted for publication in IUCr journals (*Acta Crystallographica*, *Journal of Applied Crystallography*, *Journal of Synchrotron Radiation*); however, if you intend to submit to *Acta Crystallographica Section C* or *E* or *IUCrData*, you should make sure that full publication checks are run on the final version of your CIF prior to submission.

Publication of your CIF in other journals

Please refer to the *Notes for Authors* of the relevant journal for any special instructions relating to CIF submission.

PLATON version of 04/06/2025; check.def file version of 30/05/2025

Datablock 25088 - ellipsoid plot

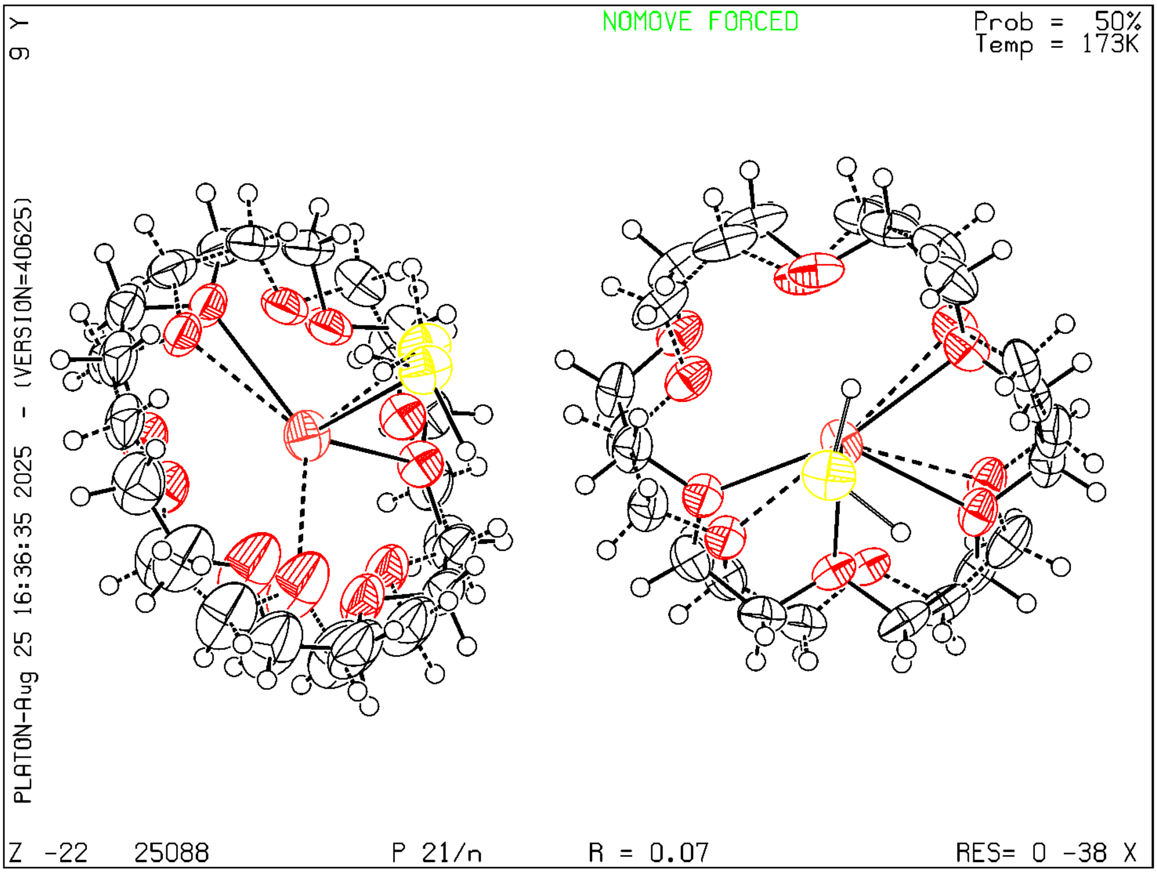

checkCIF/PLATON report

Structure factors have been supplied for datablock(s) 25136

THIS REPORT IS FOR GUIDANCE ONLY. IF USED AS PART OF A REVIEW PROCEDURE FOR PUBLICATION, IT SHOULD NOT REPLACE THE EXPERTISE OF AN EXPERIENCED CRYSTALLOGRAPHIC REFEREE.

No syntax errors found. CIF dictionary Interpreting this report

Datablock: 25136

Bond precision: C-C = 0.0071 A Wavelength=0.71073

Cell:	a=13.6226(8)	b=15.5428(9)	c=19.3700(13)
	alpha=83.814(2)	beta=88.659(2)	gamma=68.725(2)
Temperature:	153 K		

	Calculated	Reported
Volume	3799.0(4)	3799.0(4)
Space group	P 1	P 1
Hall group	P 1	P 1
Moiety formula	C45 H57 B Fe N6 P, C20 H40 K O8, 2(C4 H8 O)	C45 H57 B Fe N6 P, C20 H40 K O8, 2(C4 H8 O)
Sum formula	C73 H113 B Fe K N6 O10 P	C73 H113 B Fe K N6 O10 P
Mr	1371.42	1371.42
Dx,g cm-3	1.199	1.199
Z	2	2
Mu (mm-1)	0.332	0.332
F000	1476.0	1476.0
F000'	1477.84	
h,k,lmax	17,20,25	17,20,25
Nref	34836[17418]	34508
Tmin,Tmax	0.908,0.951	0.660,0.746
Tmin'	0.884	

Correction method= # Reported T Limits: Tmin=0.660 Tmax=0.746
AbsCorr = MULTI-SCAN

Data completeness= 1.98/0.99 Theta(max)= 27.472

R(reflections)= 0.0468(31023) wR2(reflections)= 0.1398(34508)

S = 1.027 Npar= 1864

The following ALERTS were generated. Each ALERT has the format
test-name_ALERT_alert-type_alert-level.
Click on the hyperlinks for more details of the test.

Alert level C

```
PLAT241_ALERT_2_C High   'MainMol' Ueq as Compared to Neighbors of       C59A Check
PLAT241_ALERT_2_C High   'MainMol' Ueq as Compared to Neighbors of       C63A Check
PLAT242_ALERT_2_C Low    'MainMol' Ueq as Compared to Neighbors of        K1B Check
PLAT242_ALERT_2_C Low    'MainMol' Ueq as Compared to Neighbors of        K1A Check
PLAT242_ALERT_2_C Low    'MainMol' Ueq as Compared to Neighbors of       C60A Check
PLAT260_ALERT_2_C Large Average Ueq of Residue Including       O4T      0.109 Check
PLAT260_ALERT_2_C Large Average Ueq of Residue Including       O6S      0.114 Check
PLAT260_ALERT_2_C Large Average Ueq of Residue Including       O1S      0.109 Check
PLAT260_ALERT_2_C Large Average Ueq of Residue Including       O7T      0.114 Check
PLAT260_ALERT_2_C Large Average Ueq of Residue Including      O21S      0.127 Check
PLAT260_ALERT_2_C Large Average Ueq of Residue Including      O22T      0.127 Check
PLAT341_ALERT_3_C Low Bond Precision on  C-C Bonds ...............    0.00705 Ang.
PLAT910_ALERT_3_C Missing FCF Reflection(s) Below Theta(Min)[Deg]=       2.08 Note
                1  0  0,   0  1  0,   1  1  0,   0 -1  1,  -1  0  1,   0  0  1,
                1  0  1,   0  1  1,   1  1  1,
PLAT911_ALERT_3_C Missing FCF Refl Between Thmin & STh/L=    0.600          7 Report
               -1  1  0,   2  1  0,   2  2  0,   2  3  0,  -2 -3  1,  -1 -1  2,
                0  0  2,
PLAT918_ALERT_3_C Reflection(s) with I(obs) much Smaller I(calc) .          1 Check
PLAT934_ALERT_3_C Number of (Iobs-Icalc)/Sigma(W) > 10 Outliers ..          1 Check
               -1 -2  2,
```

Alert level G

```
PLAT002_ALERT_2_G Number of Distance or Angle Restraints on AtSite         92 Note
PLAT003_ALERT_2_G Number of Uiso or U(i,j)  Restrained non-H-Atoms         90 Report
PLAT007_ALERT_5_G Number of Unrefined Donor-H Atoms ..............          2 Report
              H1B   H1A
PLAT033_ALERT_4_G Flack x Value Deviates > 3.0 * Sigma from Zero .      0.170 Note
PLAT154_ALERT_1_G The s.u.'s on the Cell Angles are Equal ..(Note)      0.002 Degree
PLAT171_ALERT_4_G The CIF-Embedded .res File Contains EADP Records         45 Report
PLAT175_ALERT_4_G The CIF-Embedded .res File Contains SAME Records         23 Report
PLAT176_ALERT_4_G The CIF-Embedded .res File Contains SADI Records          2 Report
PLAT177_ALERT_4_G The CIF-Embedded .res File Contains DELU Records         16 Report
PLAT178_ALERT_4_G The CIF-Embedded .res File Contains SIMU Records         14 Report
PLAT187_ALERT_4_G The CIF-Embedded .res File Contains RIGU Records          1 Report
PLAT188_ALERT_3_G A Non-default SIMU Restraint Value has been used     0.0100 Report
PLAT188_ALERT_3_G A Non-default SIMU Restraint Value has been used     0.0100 Report
PLAT188_ALERT_3_G A Non-default SIMU Restraint Value has been used     0.0050 Report
PLAT188_ALERT_3_G A Non-default SIMU Restraint Value has been used     0.0050 Report
PLAT301_ALERT_3_G Main Residue  Disorder ..............(Resd    1)        19% Note
PLAT302_ALERT_4_G Anion/Solvent/Minor-Residue Disorder (Resd    3)        34% Note
PLAT302_ALERT_4_G Anion/Solvent/Minor-Residue Disorder (Resd    5)       100% Note
PLAT302_ALERT_4_G Anion/Solvent/Minor-Residue Disorder (Resd    6)       100% Note
PLAT302_ALERT_4_G Anion/Solvent/Minor-Residue Disorder (Resd    7)       100% Note
PLAT302_ALERT_4_G Anion/Solvent/Minor-Residue Disorder (Resd    8)       100% Note
PLAT302_ALERT_4_G Anion/Solvent/Minor-Residue Disorder (Resd    9)       100% Note
PLAT302_ALERT_4_G Anion/Solvent/Minor-Residue Disorder (Resd   10)       100% Note
PLAT302_ALERT_4_G Anion/Solvent/Minor-Residue Disorder (Resd   11)       100% Note
PLAT302_ALERT_4_G Anion/Solvent/Minor-Residue Disorder (Resd   12)       100% Note
```

```
PLAT302_ALERT_4_G Anion/Solvent/Minor-Residue Disorder (Resd   13)       100% Note
PLAT302_ALERT_4_G Anion/Solvent/Minor-Residue Disorder (Resd   14)       100% Note
PLAT304_ALERT_4_G Non-Integer Number of Atoms in ..... (Resd    5)       9.89 Check
PLAT304_ALERT_4_G Non-Integer Number of Atoms in ..... (Resd    6)       8.94 Check
PLAT304_ALERT_4_G Non-Integer Number of Atoms in ..... (Resd    7)       9.50 Check
PLAT304_ALERT_4_G Non-Integer Number of Atoms in ..... (Resd    8)       3.11 Check
PLAT304_ALERT_4_G Non-Integer Number of Atoms in ..... (Resd    9)       4.06 Check
PLAT304_ALERT_4_G Non-Integer Number of Atoms in ..... (Resd   10)       4.07 Check
PLAT304_ALERT_4_G Non-Integer Number of Atoms in ..... (Resd   11)       2.43 Check
PLAT304_ALERT_4_G Non-Integer Number of Atoms in ..... (Resd   12)       3.50 Check
PLAT304_ALERT_4_G Non-Integer Number of Atoms in ..... (Resd   13)       4.48 Check
PLAT304_ALERT_4_G Non-Integer Number of Atoms in ..... (Resd   14)       2.01 Check
PLAT398_ALERT_2_G Deviating  C-O-C   Angle From 120 for O4T      .      106.2 Degree
PLAT398_ALERT_2_G Deviating  C-O-C   Angle From 120 for O6S      .      108.3 Degree
PLAT398_ALERT_2_G Deviating  C-O-C   Angle From 120 for O16S     .       99.9 Degree
PLAT398_ALERT_2_G Deviating  C-O-C   Angle From 120 for O7T      .      106.0 Degree
PLAT398_ALERT_2_G Deviating  C-O-C   Angle From 120 for O15T     .       84.6 Degree
PLAT398_ALERT_2_G Deviating  C-O-C   Angle From 120 for O18T     .      101.9 Degree
PLAT411_ALERT_2_G Short Inter H...H Contact  H11C     ..H8TB     .       2.09 Ang.
                                                  x,y,1+z  =     1_556 Check
PLAT411_ALERT_2_G Short Inter H...H Contact  H36B     ..H2SB     .       1.93 Ang.
                                                  x,1+y,z  =     1_565 Check
PLAT411_ALERT_2_G Short Inter H...H Contact  H35B     ..H25F     .       2.07 Ang.
                                                 -1+x,y,z  =     1_455 Check
PLAT411_ALERT_2_G Short Inter H...H Contact  H46C     ..H13V     .       1.86 Ang.
                                                 x,y,-1+z  =     1_554 Check
PLAT411_ALERT_2_G Short Inter H...H Contact  H50D     ..H15S     .       1.59 Ang.
                                              -1+x,y,-1+z  =     1_454 Check
PLAT411_ALERT_2_G Short Inter H...H Contact  H46B     ..H15T     .       2.03 Ang.
                                                 x,-1+y,z  =     1_545 Check
PLAT411_ALERT_2_G Short Inter H...H Contact  H9SB     ..H53C     .       1.66 Ang.
                                               1+x,-1+y,z  =     1_645 Check
PLAT411_ALERT_2_G Short Inter H...H Contact  H18T     ..H47A     .       2.04 Ang.
                                                    x,y,z  =     1_555 Check
PLAT411_ALERT_2_G Short Inter H...H Contact  H18T     ..H47B     .       1.85 Ang.
                                                    x,y,z  =     1_555 Check
PLAT432_ALERT_2_G Short Inter X...Y Contact  C18S     ..C47A     .       3.19 Ang.
                                                    x,y,z  =     1_555 Check
PLAT480_ALERT_4_G Long H...A H-Bond Reported H58B     ..O5A      .       2.66 Ang.
PLAT720_ALERT_4_G Number of Unusual/Non-Standard Labels ..........         40 Note
              H5AA    H5AB    H7AA    H7AB    H9AA    H9AB    H5BA    H5BB
              H7BA    H7BB    H9BA    H9BB    H2SA    H2SB    H3SA    H3SB
              H4SA    H4SB    H5SA    H5SB    H5TA    H5TB    H1TA    H1TB
              H2TA    H2TB    H3TA    H3TB    H7SA    H7SB    H8SA    H8SB
              H9SA    H9SB    H8TA    H8TB    H9TA    H9TB    H6TA    H6TB
PLAT722_ALERT_1_G Angle   Calc      109.00, Rep      110.10 Dev...       1.10 Degree
              H21U    -C21T    -H21V       1_555  1_555  1_555     # 1249 Check
PLAT860_ALERT_3_G Number of Least-Squares Restraints ............       3063 Note
PLAT912_ALERT_4_G Missing # of FCF Reflections Above STh/L=  0.600          9 Note
PLAT933_ALERT_2_G Number of HKL-OMIT Records in Embedded .res File         14 Note
                1  1 -2,  -1 -1 -1,   1  1  1,  -1  0 -1,   1  0 -1,   0  1 -1,
                1 -1  0,  -1  1  0,   2  2  0,   1  0  1,  -1 -1  2,  -2 -2  0,
               -2 -1  0,   2  1  0,
PLAT969_ALERT_5_G The 'Henn et al.' R-Factor-gap value ..........      4.415 Note
              Predicted wR2: Based on SigI**2  3.17 or SHELX Weight 13.61
PLAT978_ALERT_2_G Number C-C Bonds with Positive Residual Density.          5 Info
```

```
 0 ALERT level A = Most likely a serious problem - resolve or explain
 0 ALERT level B = A potentially serious problem, consider carefully
16 ALERT level C = Check. Ensure it is not caused by an omission or oversight
61 ALERT level G = General information/check it is not something unexpected

 2 ALERT type 1 CIF construction/syntax error, inconsistent or missing data
31 ALERT type 2 Indicator that the structure model may be wrong or deficient
11 ALERT type 3 Indicator that the structure quality may be low
31 ALERT type 4 Improvement, methodology, query or suggestion
 2 ALERT type 5 Informative message, check
```

It is advisable to attempt to resolve as many as possible of the alerts in all categories. Often the minor alerts point to easily fixed oversights, errors and omissions in your CIF or refinement strategy, so attention to these fine details can be worthwhile. In order to resolve some of the more serious problems it may be necessary to carry out additional measurements or structure refinements. However, the purpose of your study may justify the reported deviations and the more serious of these should normally be commented upon in the discussion or experimental section of a paper or in the "special_details" fields of the CIF. checkCIF was carefully designed to identify outliers and unusual parameters, but every test has its limitations and alerts that are not important in a particular case may appear. Conversely, the absence of alerts does not guarantee there are no aspects of the results needing attention. It is up to the individual to critically assess their own results and, if necessary, seek expert advice.

Publication of your CIF in IUCr journals

A basic structural check has been run on your CIF. These basic checks will be run on all CIFs submitted for publication in IUCr journals (*Acta Crystallographica*, *Journal of Applied Crystallography*, *Journal of Synchrotron Radiation*); however, if you intend to submit to *Acta Crystallographica Section C* or *E* or *IUCrData*, you should make sure that full publication checks are run on the final version of your CIF prior to submission.

Publication of your CIF in other journals

Please refer to the *Notes for Authors* of the relevant journal for any special instructions relating to CIF submission.

PLATON version of 04/06/2025; check.def file version of 30/05/2025

Datablock 25136 - ellipsoid plot

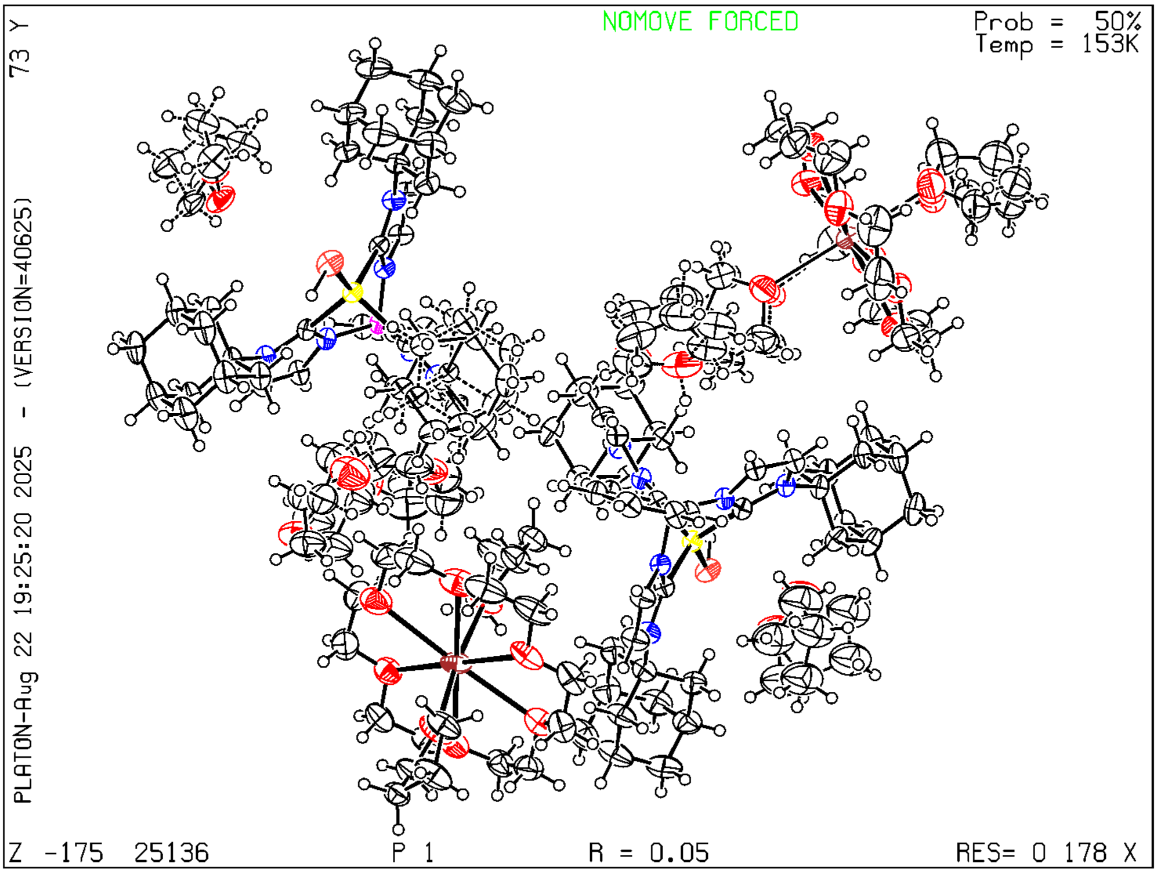

checkCIF/PLATON report

Structure factors have been supplied for datablock(s) 25184

THIS REPORT IS FOR GUIDANCE ONLY. IF USED AS PART OF A REVIEW PROCEDURE FOR PUBLICATION, IT SHOULD NOT REPLACE THE EXPERTISE OF AN EXPERIENCED CRYSTALLOGRAPHIC REFEREE.

No syntax errors found. CIF dictionary Interpreting this report

Datablock: 25184

Bond precision:	C-C = 0.0033 A		Wavelength=0.71073
Cell:	a=16.4945(5)	b=17.7267(5)	c=25.3741(7)
	alpha=90	beta=103.714(1)	gamma=90
Temperature:	153 K		

	Calculated	Reported
Volume	7207.7(4)	7207.7(4)
Space group	P 21/n	P 21/n
Hall group	-P 2yn	-P 2yn
Moiety formula	2(C45 H57 As B Fe N6), 2(C18 H36 K N2 O6), 5(C4 H8 O)	(C45 H57 As B Fe N6), (C18 H36 K N2 O6), 2.5(C4 H8 O)
Sum formula	C146 H226 As2 B2 Fe2 K2 N16 O17	C73 H113 As B Fe K N8 O8.50
Mr	2838.79	1419.39
Dx,g cm-3	1.308	1.308
Z	2	4
Mu (mm-1)	0.783	0.783
F000	3032.0	3032.0
F000’	3035.37	
h,k,lmax	20,22,32	20,22,32
Nref	15550	15531
Tmin,Tmax	0.744,0.817	0.698,0.746
Tmin’	0.730	

Correction method= # Reported T Limits: Tmin=0.698 Tmax=0.746
AbsCorr = MULTI-SCAN

Data completeness= 0.999 Theta(max)= 26.889

R(reflections)= 0.0385(13889)   wR2(reflections)= 0.1151(15531)

S = 1.027   Npar= 853

The following ALERTS were generated. Each ALERT has the format
test-name_ALERT_alert-type_alert-level.
Click on the hyperlinks for more details of the test.

Alert level B

```
PLAT232_ALERT_2_B Hirshfeld Test Diff (M-X)  As1      --Fe1      .       23.1 s.u.
```

Author Response: As1 is located in large pocket and displays larger displacement.

Alert level C

```
PLAT041_ALERT_1_C Calc. and Reported SumFormula    Strings  Differ     Please Check
              Calc: C146 H226 As2 B2 Fe2 K2 N16 O17
              Rep.: C73 H113 As B Fe K N8 O8.50
PLAT042_ALERT_1_C Calc. and Reported MoietyFormula Strings  Differ     Please Check
              Calc: 2(C45 H57 As B Fe N6), 2(C18 H36 K N2 O6), 5(C
                    4 H8 O)
              Rep.: (C45 H57 As B Fe N6), (C18 H36 K N2 O6), 2.5(C
                    4 H8 O)
PLAT243_ALERT_4_C High   'Solvent' Ueq as Compared to Neighbors of        C2S Check
PLAT243_ALERT_4_C High   'Solvent' Ueq as Compared to Neighbors of        C3S Check
PLAT243_ALERT_4_C High   'Solvent' Ueq as Compared to Neighbors of        O2S Check
PLAT243_ALERT_4_C High   'Solvent' Ueq as Compared to Neighbors of        C6S Check
PLAT244_ALERT_4_C Low    'Solvent' Ueq as Compared to Neighbors of        C1S Check
PLAT244_ALERT_4_C Low    'Solvent' Ueq as Compared to Neighbors of        C5S Check
PLAT244_ALERT_4_C Low    'Solvent' Ueq as Compared to Neighbors of        C7S Check
PLAT360_ALERT_2_C Short  C(sp3)-C(sp3) Bond  C3S      - C4S      .      1.41 Ang.
PLAT420_ALERT_2_C D-H Bond Without Acceptor  As1      --H1AS     .     Please Check
PLAT910_ALERT_3_C Missing # of FCF Reflection(s) Below Theta(Min).          8 Note
                1  1  0,  -1  0  1,   1  0  1,  -1  1  1,   0  1  1,   1  1  1,
                0  0  2,   0  1  2,
PLAT911_ALERT_3_C Missing FCF Refl Between Thmin & STh/L=    0.600          4 Report
                0  4  0,   0 12  0,   1  0  5,   2  4  6,
```

Alert level G

```
PLAT002_ALERT_2_G Number of Distance or Angle Restraints on AtSite         10 Note
PLAT003_ALERT_2_G Number of Uiso or U(i,j)  Restrained non-H-Atoms          2 Report
PLAT045_ALERT_1_G Calculated and Reported Z Differ by a Factor ...      0.500 Check
PLAT171_ALERT_4_G The CIF-Embedded .res File Contains EADP Records          2 Report
PLAT175_ALERT_4_G The CIF-Embedded .res File Contains SAME Records          3 Report
PLAT177_ALERT_4_G The CIF-Embedded .res File Contains DELU Records          1 Report
PLAT187_ALERT_4_G The CIF-Embedded .res File Contains RIGU Records          2 Report
PLAT192_ALERT_3_G A Non-default DELU Restraint Value for First Par     0.0050 Report
PLAT232_ALERT_2_G Hirshfeld Test Diff (M-X)  Fe1      --C27      .        5.6 s.u.
```

Author Response: As1 is located in large pocket and displays larger displacement.

```
PLAT299_ALERT_4_G Atom Site Occupancy Constrained at ............        0.5 Check
              O3S     C9S     C10S    C11S    C12S    H10A    H10B    H11C
              H11D    H12C    H12D    H9SA    H9SB
PLAT302_ALERT_4_G Anion/Solvent/Minor-Residue Disorder (Resd    5)       100% Note
PLAT304_ALERT_4_G Non-Integer Number of Atoms in ..... (Resd    5)       6.50 Check
PLAT398_ALERT_2_G Deviating  C-O-C   Angle From 120 for O1S      .      109.5 Degree
PLAT398_ALERT_2_G Deviating  C-O-C   Angle From 120 for O2S      .      109.5 Degree
PLAT398_ALERT_2_G Deviating  C-O-C   Angle From 120 for O3S      .      103.3 Degree
PLAT411_ALERT_2_G Short Inter H...H Contact  H12C     ..H34      .       2.14 Ang.
                                             1-x,1-y,1-z  =      3_666 Check
PLAT480_ALERT_4_G Long H...A H-Bond Reported H4SB     ..O2S      .       2.70 Ang.
PLAT720_ALERT_4_G Number of Unusual/Non-Standard Labels ..........         19 Note
              H1AS    H1SA    H1SB    H2SA    H2SB    H3SA    H3SB    H4SA
              H4SB    H5SA    H5SB    H6SA    H6SB    H7SA    H7SB    H8SA
              H8SB    H9SA    H9SB
PLAT789_ALERT_4_G Atoms with Negative _atom_site_disorder_group  #         13 Check
PLAT822_ALERT_4_G CIF-embedded .res Contains Negative PART Numbers          1 Check
PLAT860_ALERT_3_G Number of Least-Squares Restraints ............         964 Note
PLAT912_ALERT_4_G Missing # of FCF Reflections Above STh/L=  0.600          7 Note
PLAT913_ALERT_3_G Missing # of Very Strong Reflections in FCF ....          2 Note
                0  4  0,   1  0  5,
PLAT933_ALERT_2_G Number of HKL-OMIT Records in Embedded .res File          4 Note
                0  1  2,   0 12  0,   0  1  2,   2  4  6,
PLAT965_ALERT_2_G The SHELXL WEIGHT Optimisation has not Converged     Please Check
PLAT969_ALERT_5_G The 'Henn et al.' R-Factor-gap value ..........      7.527 Note
              Predicted wR2: Based on SigI**2  1.53 or SHELX Weight 11.20
PLAT978_ALERT_2_G Number C-C Bonds with Positive Residual Density.          7 Info
```

```
   0 ALERT level A = Most likely a serious problem - resolve or explain
   1 ALERT level B = A potentially serious problem, consider carefully
  13 ALERT level C = Check. Ensure it is not caused by an omission or oversight
  27 ALERT level G = General information/check it is not something unexpected

   3 ALERT type 1 CIF construction/syntax error, inconsistent or missing data
  13 ALERT type 2 Indicator that the structure model may be wrong or deficient
   5 ALERT type 3 Indicator that the structure quality may be low
  19 ALERT type 4 Improvement, methodology, query or suggestion
   1 ALERT type 5 Informative message, check
```

It is advisable to attempt to resolve as many as possible of the alerts in all categories. Often the minor alerts point to easily fixed oversights, errors and omissions in your CIF or refinement strategy, so attention to these fine details can be worthwhile. In order to resolve some of the more serious problems it may be necessary to carry out additional measurements or structure refinements. However, the purpose of your study may justify the reported deviations and the more serious of these should normally be commented upon in the discussion or experimental section of a paper or in the "special_details" fields of the CIF. checkCIF was carefully designed to identify outliers and unusual parameters, but every test has its limitations and alerts that are not important in a particular case may appear. Conversely, the absence of alerts does not guarantee there are no aspects of the results needing attention. It is up to the individual to critically assess their own results and, if necessary, seek expert advice.

Publication of your CIF in IUCr journals

A basic structural check has been run on your CIF. These basic checks will be run on all CIFs submitted for publication in IUCr journals (*Acta Crystallographica*, *Journal of Applied Crystallography*, *Journal of Synchrotron Radiation*); however, if you intend to submit to *Acta Crystallographica Section C* or *E* or *IUCrData*, you should make sure that full publication checks are run on the final version of your CIF prior to submission.

Publication of your CIF in other journals

Please refer to the *Notes for Authors* of the relevant journal for any special instructions relating to CIF submission.

PLATON version of 02/02/2025; check.def file version of 02/02/2025

Datablock 25184 - ellipsoid plot

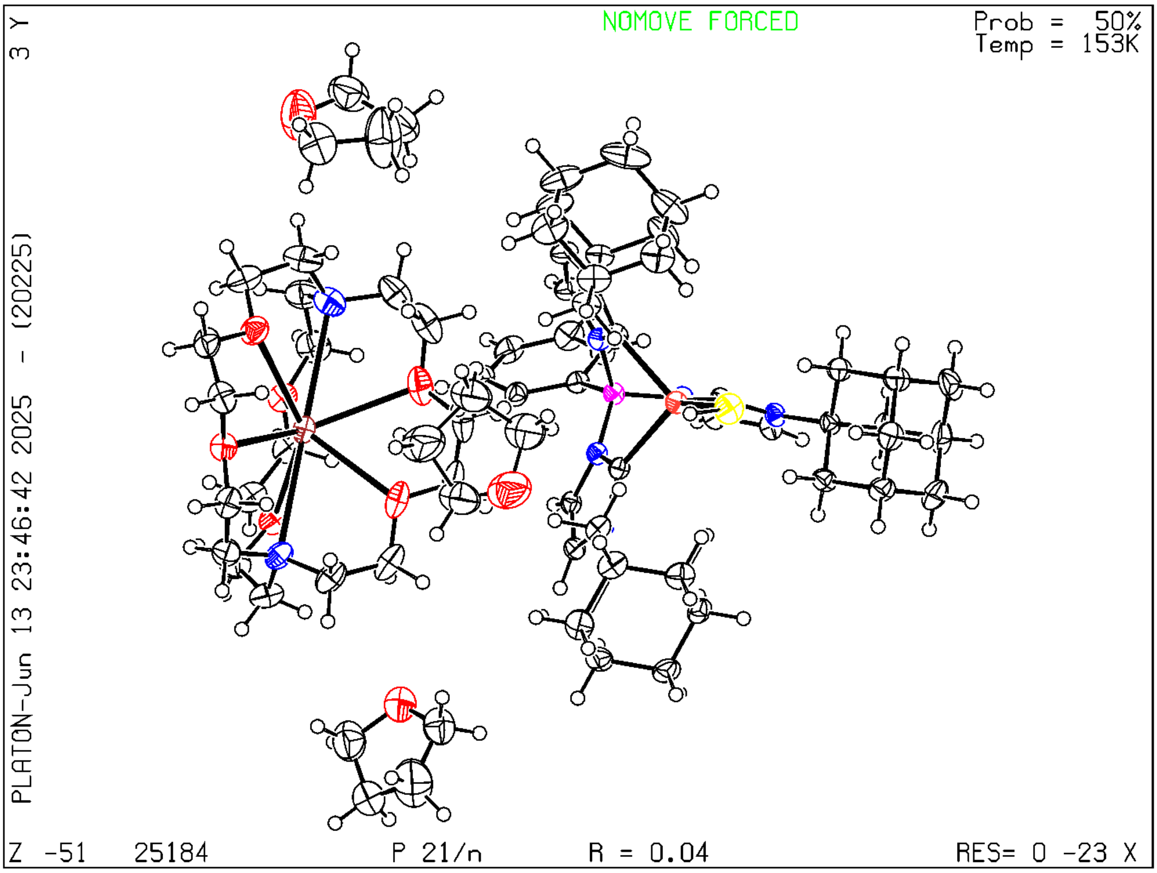

checkCIF/PLATON report

Structure factors have been supplied for datablock(s) 25139

THIS REPORT IS FOR GUIDANCE ONLY. IF USED AS PART OF A REVIEW PROCEDURE FOR PUBLICATION, IT SHOULD NOT REPLACE THE EXPERTISE OF AN EXPERIENCED CRYSTALLOGRAPHIC REFEREE.

No syntax errors found. CIF dictionary Interpreting this report

Datablock: 25139

```
Bond precision:   C-C = 0.0117 A           Wavelength=0.71073

Cell:              a=13.8714(6)     b=17.6014(8)          c=19.4029(9)
                   alpha=90         beta=93.0743(14)      gamma=90
Temperature:       153 K
```

	Calculated	Reported
Volume	4730.5(4)	4730.5(4)
Space group	P 21/c	P 21/c
Hall group	-P 2ybc	-P 2ybc
Moiety formula	C45 H57.18 As0.59 B Cl0.41 Fe N6, 2(C4 H8 O)	0.59(C45 H58 As B Fe N6), 0.41(C45 H56 B Cl Fe N6), 2(C4 H8 O)
Sum formula	C53 H73.18 As0.59 B Cl0.41 Fe N6 O2	C53 H73.18 As0.59 B Cl0.41 Fe N6 O2
Mr	951.79	951.80
Dx,g cm-3	1.336	1.336
Z	4	4
Mu (mm-1)	0.801	0.801
F000	2026.6	2027.0
F000'	2028.85	
h,k,lmax	16,21,23	16,20,23
Nref	8442	8384
Tmin,Tmax	0.888,0.922	0.368,0.745
Tmin'	0.888	

```
Correction method= # Reported T Limits: Tmin=0.368 Tmax=0.745
AbsCorr = MULTI-SCAN

Data completeness= 0.993           Theta(max)= 25.116
```

```
R(reflections)= 0.0802( 6323)                    wR2(reflections)=
                                                 0.2185( 8384)
S = 1.063                  Npar= 614
```

The following ALERTS were generated. Each ALERT has the format
test-name_ALERT_alert-type_alert-level.
Click on the hyperlinks for more details of the test.

Alert level C

```
RINTA01_ALERT_3_C  The value of Rint is greater than 0.12
            Rint given   0.176
PLAT042_ALERT_1_C Calc. and Reported MoietyFormula Strings  Differ     Please Check
              Calc: C45 H57.18 As0.59 B Cl0.41 Fe N6, 2(C4 H8 O)
              Rep.: 0.59(C45 H58 As B Fe N6), 0.41(C45 H56 B Cl Fe
                     N6), 2(C4 H8 O)
PLAT077_ALERT_4_C Unit Cell Contains Non-integer Number of Atoms .     Please Check
PLAT341_ALERT_3_C Low Bond Precision on  C-C Bonds ...............    0.01169 Ang.
PLAT420_ALERT_2_C D-H Bond Without Acceptor  As1       --H1A      .     Please Check
PLAT420_ALERT_2_C D-H Bond Without Acceptor  As1       --H1B      .     Please Check
PLAT910_ALERT_3_C Missing FCF Reflection(s) Below Theta(Min)[Deg]=       2.18 Note
                1  0  0,   1  1  0,  -1  1  1,   0  1  1,   0  0  2,
PLAT911_ALERT_3_C Missing FCF Refl Between Thmin & STh/L=    0.597         52 Report
                0  8  0,  16  5  1,   7 19  1,   5 20  1,  -7 19  2,  15  8  3,
               -1 17  4,   4 20  4,  15  6  6,  12 13  6, -11 15  6,  15  5  7,
               11 14  7,   8 17  7,  -1 20  7,   0 20  7,  15  3  8,  12 12  8,
                6 18  8,  14  5 10,  -5 18 10,  13  7 11, -11 12 12,   6 16 12,
               -5 17 12,  12  7 13,  12  5 14,  11  8 14,  10 10 14,  12  1 15,
                   (   22 More Missing: see the .ckf listing file)
```

Alert level G

```
PLAT002_ALERT_2_G Number of Distance or Angle Restraints on AtSite         20 Note
PLAT003_ALERT_2_G Number of Uiso or U(i,j)  Restrained non-H-Atoms         10 Report
PLAT068_ALERT_1_G Reported F000 Differs from Calcd (or Missing)...     Please Check
PLAT083_ALERT_2_G SHELXL Second Parameter in WGHT  Unusually Large      19.18 Why ?
PLAT171_ALERT_4_G The CIF-Embedded .res File Contains EADP Records         11 Report
PLAT175_ALERT_4_G The CIF-Embedded .res File Contains SAME Records          8 Report
PLAT177_ALERT_4_G The CIF-Embedded .res File Contains DELU Records          1 Report
PLAT187_ALERT_4_G The CIF-Embedded .res File Contains RIGU Records          1 Report
PLAT232_ALERT_2_G Hirshfeld Test Diff (M-X)  As1       --Fe1      .      11.2 s.u.
PLAT232_ALERT_2_G Hirshfeld Test Diff (M-X)  Fe1       --Cl1      .      11.4 s.u.
PLAT301_ALERT_3_G Main Residue  Disorder ..............(Resd    1)         2% Note
PLAT302_ALERT_4_G Anion/Solvent/Minor-Residue Disorder (Resd    2)       100% Note
PLAT302_ALERT_4_G Anion/Solvent/Minor-Residue Disorder (Resd    3)       100% Note
PLAT302_ALERT_4_G Anion/Solvent/Minor-Residue Disorder (Resd    4)       100% Note
PLAT302_ALERT_4_G Anion/Solvent/Minor-Residue Disorder (Resd    5)       100% Note
PLAT304_ALERT_4_G Non-Integer Number of Atoms in ..... (Resd    1)     111.18 Check
PLAT304_ALERT_4_G Non-Integer Number of Atoms in ..... (Resd    2)       8.72 Check
PLAT304_ALERT_4_G Non-Integer Number of Atoms in ..... (Resd    3)       8.63 Check
PLAT304_ALERT_4_G Non-Integer Number of Atoms in ..... (Resd    4)       4.28 Check
PLAT304_ALERT_4_G Non-Integer Number of Atoms in ..... (Resd    5)       4.37 Check
PLAT398_ALERT_2_G Deviating  C-O-C   Angle From 120 for O1S       .     107.6 Degree
PLAT398_ALERT_2_G Deviating  C-O-C   Angle From 120 for O2S       .     107.8 Degree
PLAT398_ALERT_2_G Deviating  C-O-C   Angle From 120 for O1D       .     107.4 Degree
```

```
PLAT398_ALERT_2_G Deviating  C-O-C   Angle From 120 for O2D      .      107.2 Degree
PLAT410_ALERT_2_G Short Intra H...H Contact  H1A      ..H9B      .       2.10 Ang.
                                                  x,y,z  =      1_555 Check
PLAT411_ALERT_2_G Short Inter H...H Contact  H7A      ..H7DA     .       1.95 Ang.
                                             -x,1/2+y,1/2-z  =      2_555 Check
PLAT411_ALERT_2_G Short Inter H...H Contact  H7A      ..H7DB     .       1.94 Ang.
                                             -x,1/2+y,1/2-z  =      2_555 Check
PLAT411_ALERT_2_G Short Inter H...H Contact  H25B     ..H7DB     .       1.97 Ang.
                                                 -x,1-y,1-z  =      3_566 Check
PLAT720_ALERT_4_G Number of Unusual/Non-Standard Labels ..........         32 Note
              H1SA    H1SB    H2SA    H2SB    H3SA    H3SB    H4SA    H4SB
              H1DA    H1DB    H2DA    H2DB    H3DA    H3DB    H4DA    H4DB
              H5SA    H5SB    H6SA    H6SB    H7SA    H7SB    H8SA    H8SB
              H6DA    H6DB    H5DA    H5DB    H7DA    H7DB    H8DA    H8DB
PLAT860_ALERT_3_G Number of Least-Squares Restraints .............        812 Note
PLAT870_ALERT_4_G ALERTS Related to Twinning Effects Suppressed ..          ! Info
PLAT909_ALERT_3_G Percentage of I>2sig(I) Data at Theta(Max) Still        46% Note
PLAT931_ALERT_5_G CIFcalcFCF  Twin Law ( 1 0 0)         Est.d BASF       0.31 Check
PLAT933_ALERT_2_G Number of HKL-OMIT Records in Embedded .res File          2 Note
                1  1  0,  -1  1  1,
PLAT941_ALERT_3_G Average HKL Measurement Multiplicity ..........        1.0 Low
PLAT969_ALERT_5_G The 'Henn et al.' R-Factor-gap value ..........      2.110 Note
              Predicted wR2: Based on SigI**2 10.35 or SHELX Weight 20.55
```

---

0 **ALERT level A** = Most likely a serious problem - resolve or explain
0 **ALERT level B** = A potentially serious problem, consider carefully
8 **ALERT level C** = Check. Ensure it is not caused by an omission or oversight
36 **ALERT level G** = General information/check it is not something unexpected

2 ALERT type 1 CIF construction/syntax error, inconsistent or missing data
16 ALERT type 2 Indicator that the structure model may be wrong or deficient
8 ALERT type 3 Indicator that the structure quality may be low
16 ALERT type 4 Improvement, methodology, query or suggestion
2 ALERT type 5 Informative message, check

It is advisable to attempt to resolve as many as possible of the alerts in all categories. Often the minor alerts point to easily fixed oversights, errors and omissions in your CIF or refinement strategy, so attention to these fine details can be worthwhile. In order to resolve some of the more serious problems it may be necessary to carry out additional measurements or structure refinements. However, the purpose of your study may justify the reported deviations and the more serious of these should normally be commented upon in the discussion or experimental section of a paper or in the "special_details" fields of the CIF. checkCIF was carefully designed to identify outliers and unusual parameters, but every test has its limitations and alerts that are not important in a particular case may appear. Conversely, the absence of alerts does not guarantee there are no aspects of the results needing attention. It is up to the individual to critically assess their own results and, if necessary, seek expert advice.

Publication of your CIF in IUCr journals

A basic structural check has been run on your CIF. These basic checks will be run on all CIFs submitted for publication in IUCr journals (*Acta Crystallographica*, *Journal of Applied Crystallography*, *Journal of Synchrotron Radiation*); however, if you intend to submit to *Acta Crystallographica Section C* or *E* or *IUCrData*, you should make sure that full publication checks are run on the final version of your CIF prior to submission.

Publication of your CIF in other journals

Please refer to the *Notes for Authors* of the relevant journal for any special instructions relating to CIF submission.

PLATON version of 04/06/2025; check.def file version of 30/05/2025

Datablock 25139 - ellipsoid plot

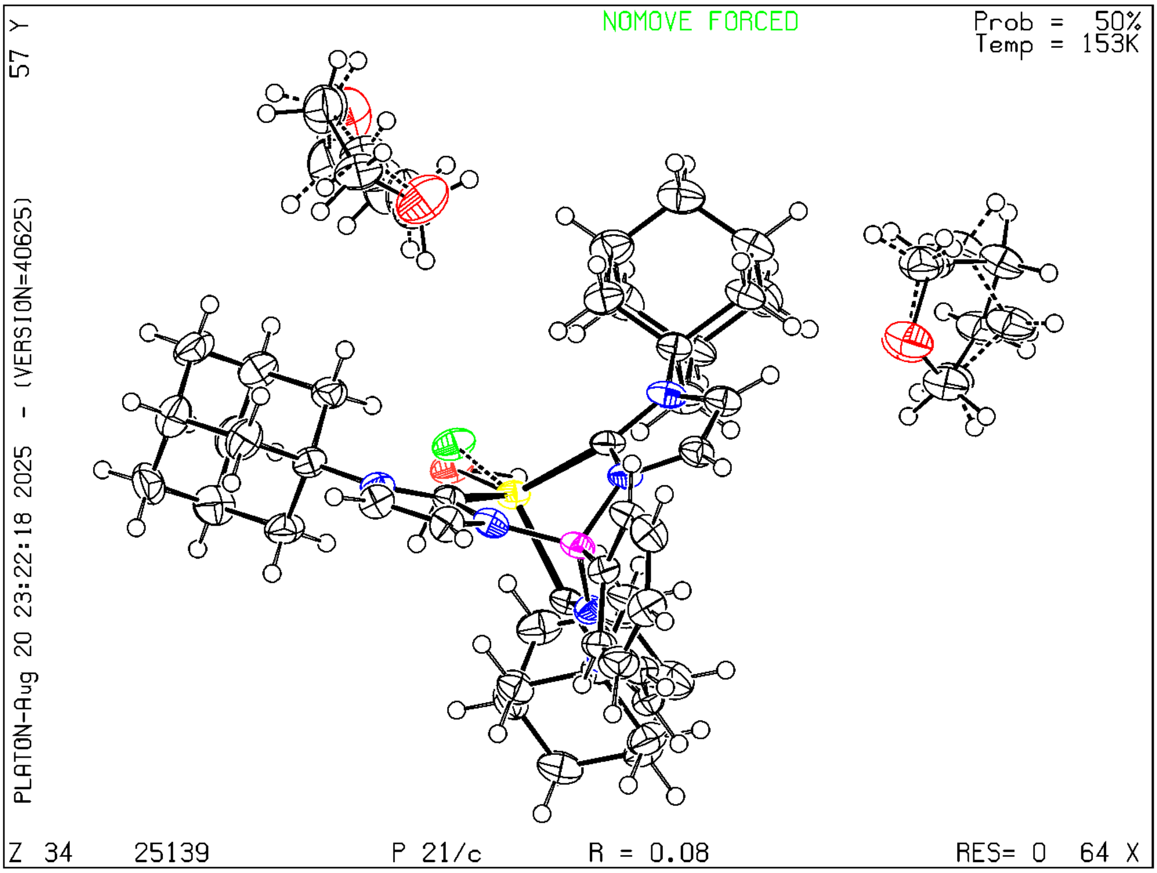

checkCIF/PLATON report

Structure factors have been supplied for datablock(s) 25075

THIS REPORT IS FOR GUIDANCE ONLY. IF USED AS PART OF A REVIEW PROCEDURE FOR PUBLICATION, IT SHOULD NOT REPLACE THE EXPERTISE OF AN EXPERIENCED CRYSTALLOGRAPHIC REFEREE.

No syntax errors found. CIF dictionary Interpreting this report

Datablock: 25075

Bond precision:	C-C = 0.0027 A		Wavelength=0.71073
Cell:	a=16.4587(4)	b=17.7400(4)	c=25.3434(6)
	alpha=90	beta=103.775(1)	gamma=90
Temperature:	153 K		

	Calculated	Reported
Volume	7186.9(3)	7186.9(3)
Space group	P 21/n	P 21/n
Hall group	-P 2yn	-P 2yn
Moiety formula	2(C45 H57 B Fe N6 P), 2(C18 H36 K N2 O6), 5(C4 H8 O)	C45 H57 B Fe N6 P, C18 H36 K N2 O6, 2.5(C4 H8 O)
Sum formula	C146 H226 B2 Fe2 K2 N16 O17 P2	C73 H113 B Fe K N8 O8.50 P
Mr	2750.89	1375.44
Dx,g cm-3	1.271	1.271
Z	2	4
Mu (mm-1)	0.351	0.351
F000	2960.0	2960.0
F000'	2963.63	
h,k,lmax	21,23,33	21,23,33
Nref	17845	17835
Tmin,Tmax	0.938,0.951	0.720,0.746
Tmin'	0.873	

Correction method= # Reported T Limits: Tmin=0.720 Tmax=0.746
AbsCorr = MULTI-SCAN

Data completeness= 0.999 Theta(max)= 28.288

```
R(reflections)= 0.0402( 15347)                        wR2(reflections)=
                                                      0.1187( 17835)
S = 1.034                   Npar= 853
```

The following ALERTS were generated. Each ALERT has the format
test-name_ALERT_alert-type_alert-level.
Click on the hyperlinks for more details of the test.

Alert level C

```
PLAT041_ALERT_1_C Calc. and Reported SumFormula    Strings  Differ     Please Check
              Calc: C146 H226 B2 Fe2 K2 N16 O17 P2
              Rep.: C73 H113 B Fe K N8 O8.50 P
PLAT042_ALERT_1_C Calc. and Reported MoietyFormula Strings  Differ     Please Check
              Calc: 2(C45 H57 B Fe N6 P), 2(C18 H36 K N2 O6), 5(C4
                     H8 O)
              Rep.: C45 H57 B Fe N6 P, C18 H36 K N2 O6, 2.5(C4 H8
                    O)
PLAT243_ALERT_4_C High   'Solvent' Ueq as Compared to Neighbors of        C2S Check
PLAT243_ALERT_4_C High   'Solvent' Ueq as Compared to Neighbors of        O2S Check
PLAT243_ALERT_4_C High   'Solvent' Ueq as Compared to Neighbors of        C6S Check
PLAT244_ALERT_4_C Low    'Solvent' Ueq as Compared to Neighbors of        C1S Check
PLAT244_ALERT_4_C Low    'Solvent' Ueq as Compared to Neighbors of        C5S Check
PLAT910_ALERT_3_C Missing # of FCF Reflection(s) Below Theta(Min).          8 Note
                1  1  0,  -1  0  1,   1  0  1,  -1  1  1,   0  1  1,   1  1  1,
                0  0  2,   0  1  2,
PLAT911_ALERT_3_C Missing FCF Refl Between Thmin & STh/L=    0.600          2 Report
                9  9  3,   3  0 15,
```

Alert level G

```
PLAT002_ALERT_2_G Number of Distance or Angle Restraints on AtSite         10 Note
PLAT007_ALERT_5_G Number of Unrefined Donor-H Atoms .............           1 Report
              H1P
PLAT045_ALERT_1_G Calculated and Reported Z Differ by a Factor ...      0.500 Check
PLAT171_ALERT_4_G The CIF-Embedded .res File Contains EADP Records          2 Report
PLAT175_ALERT_4_G The CIF-Embedded .res File Contains SAME Records          3 Report
PLAT187_ALERT_4_G The CIF-Embedded .res File Contains RIGU Records          1 Report
PLAT299_ALERT_4_G Atom Site Occupancy Constrained at ............         0.5 Check
              O3S     C9S     C10S    C11S    C12S    H9S1    H9S2    H10S
              H10T    H11S    H11T    H12S    H12T
PLAT302_ALERT_4_G Anion/Solvent/Minor-Residue Disorder (Resd    5)       100% Note
PLAT304_ALERT_4_G Non-Integer Number of Atoms in ..... (Resd    5)       6.50 Check
PLAT398_ALERT_2_G Deviating  C-O-C   Angle From 120 for O1S      .      109.0 Degree
PLAT398_ALERT_2_G Deviating  C-O-C   Angle From 120 for O2S      .      109.1 Degree
PLAT398_ALERT_2_G Deviating  C-O-C   Angle From 120 for O3S      .      101.8 Degree
PLAT411_ALERT_2_G Short Inter H...H Contact  H12S     ..H34      .       2.09 Ang.
                                          1-x,1-y,1-z  =     3_666 Check
PLAT480_ALERT_4_G Long H...A H-Bond Reported H4S      ..O2S      .       2.65 Ang.
PLAT720_ALERT_4_G Number of Unusual/Non-Standard Labels .........           5 Note
              H5AB    H7AB    H9AB    H9S1    H9S2
PLAT789_ALERT_4_G Atoms with Negative _atom_site_disorder_group  #         13 Check
PLAT822_ALERT_4_G CIF-embedded .res Contains Negative PART Numbers          1 Check
PLAT860_ALERT_3_G Number of Least-Squares Restraints ............          56 Note
PLAT933_ALERT_2_G Number of HKL-OMIT Records in Embedded .res File          6 Note
```

```
               1  1  1,   0  1  2,   1  1  1,   0  1  2,   9  9  3,   3  0 15,
PLAT965_ALERT_2_G The SHELXL WEIGHT Optimisation has not Converged     Please Check
PLAT969_ALERT_5_G The 'Henn et al.' R-Factor-gap value ..........      7.204 Note
              Predicted wR2: Based on SigI**2  1.65 or SHELX Weight 11.49
PLAT978_ALERT_2_G Number C-C Bonds with Positive Residual Density.        11 Info
```

```
  0 ALERT level A = Most likely a serious problem - resolve or explain
  0 ALERT level B = A potentially serious problem, consider carefully
  9 ALERT level C = Check. Ensure it is not caused by an omission or oversight
 22 ALERT level G = General information/check it is not something unexpected

  3 ALERT type 1 CIF construction/syntax error, inconsistent or missing data
  8 ALERT type 2 Indicator that the structure model may be wrong or deficient
  3 ALERT type 3 Indicator that the structure quality may be low
 15 ALERT type 4 Improvement, methodology, query or suggestion
  2 ALERT type 5 Informative message, check
```

It is advisable to attempt to resolve as many as possible of the alerts in all categories. Often the minor alerts point to easily fixed oversights, errors and omissions in your CIF or refinement strategy, so attention to these fine details can be worthwhile. In order to resolve some of the more serious problems it may be necessary to carry out additional measurements or structure refinements. However, the purpose of your study may justify the reported deviations and the more serious of these should normally be commented upon in the discussion or experimental section of a paper or in the "special_details" fields of the CIF. checkCIF was carefully designed to identify outliers and unusual parameters, but every test has its limitations and alerts that are not important in a particular case may appear. Conversely, the absence of alerts does not guarantee there are no aspects of the results needing attention. It is up to the individual to critically assess their own results and, if necessary, seek expert advice.

Publication of your CIF in IUCr journals

A basic structural check has been run on your CIF. These basic checks will be run on all CIFs submitted for publication in IUCr journals (*Acta Crystallographica*, *Journal of Applied Crystallography*, *Journal of Synchrotron Radiation*); however, if you intend to submit to *Acta Crystallographica Section C* or *E* or *IUCrData*, you should make sure that full publication checks are run on the final version of your CIF prior to submission.

Publication of your CIF in other journals

Please refer to the *Notes for Authors* of the relevant journal for any special instructions relating to CIF submission.

PLATON version of 02/02/2025; check.def file version of 02/02/2025

Datablock 25075 - ellipsoid plot

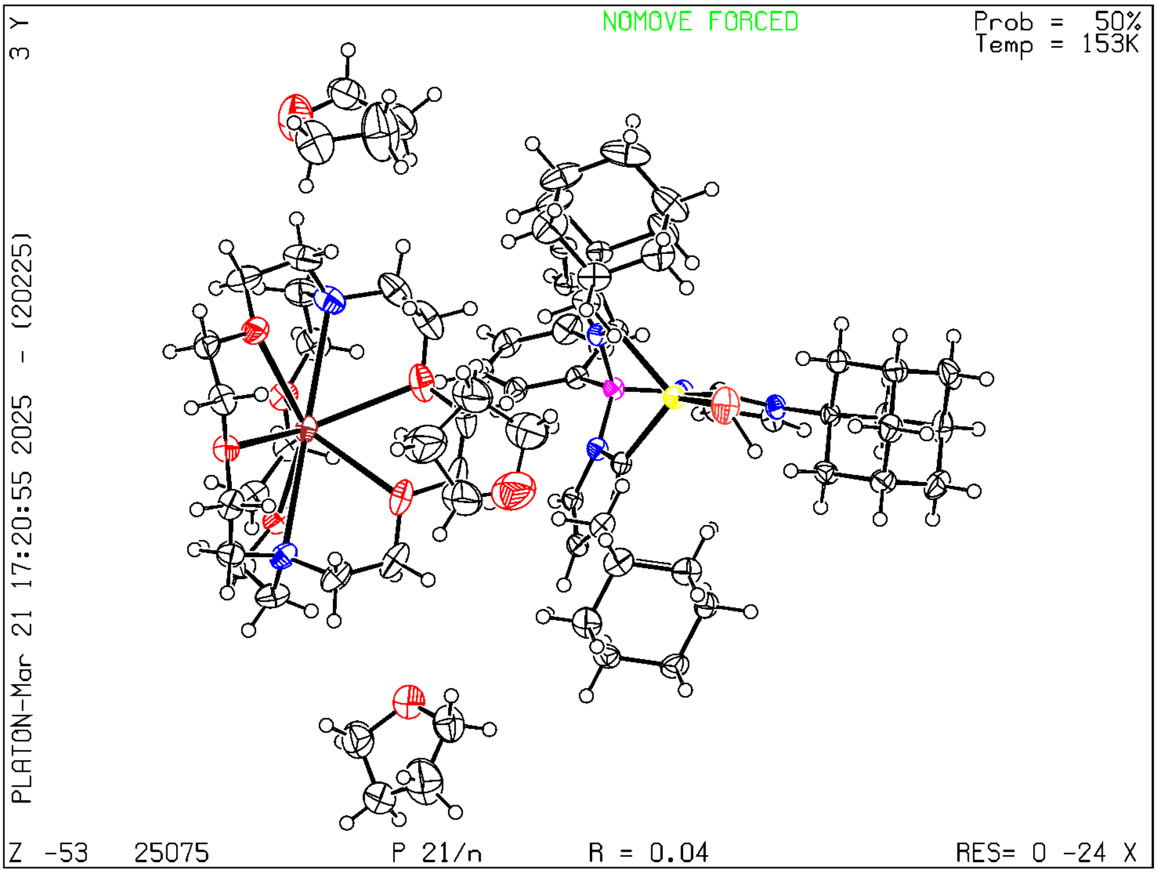

checkCIF/PLATON report

Structure factors have been supplied for datablock(s) 25062

THIS REPORT IS FOR GUIDANCE ONLY. IF USED AS PART OF A REVIEW PROCEDURE FOR PUBLICATION, IT SHOULD NOT REPLACE THE EXPERTISE OF AN EXPERIENCED CRYSTALLOGRAPHIC REFEREE.

No syntax errors found. CIF dictionary Interpreting this report

Datablock: 25062

Bond precision:	C-C = 0.0077 A		Wavelength=0.71073
Cell:	a=21.6676(11)	b=19.8700(11)	c=14.4839(8)
	alpha=90	beta=90	gamma=90
Temperature:	153 K		

	Calculated	Reported
Volume	6235.8(6)	6235.8(6)
Space group	P n a 21	P n a 21
Hall group	P 2c -2n	P 2c -2n
Moiety formula	C57 H81 B Fe K N6 O6 P, C7 H8	C57 H81 B Fe K N6 O6 P, C7 H8
Sum formula	C64 H89 B Fe K N6 O6 P	C64 H89 B Fe K N6 O6 P
Mr	1175.15	1175.14
Dx,g cm-3	1.252	1.252
Z	4	4
Mu (mm-1)	0.389	0.389
F000	2512.0	2512.0
F000’	2515.44	
h,k,lmax	25,23,17	25,23,17
Nref	11018[5747]	10990
Tmin,Tmax	0.944,0.956	0.705,0.745
Tmin’	0.944	

Correction method= # Reported T Limits: Tmin=0.705 Tmax=0.745
AbsCorr = MULTI-SCAN

Data completeness= 1.91/1.00 Theta(max)= 25.040

R(reflections)= 0.0464(10409) wR2(reflections)= 0.1229(10990)

S = 1.070 Npar= 759

The following ALERTS were generated. Each ALERT has the format
test-name_ALERT_alert-type_alert-level.
Click on the hyperlinks for more details of the test.

Alert level C

```
STRVA01_ALERT_4_C           Flack test results are ambiguous.
           From the CIF: _refine_ls_abs_structure_Flack     0.470
           From the CIF: _refine_ls_abs_structure_Flack_su     0.020
PLAT090_ALERT_3_C Poor Data / Parameter Ratio (Zmax > 18) ........       7.55 Note
PLAT094_ALERT_2_C Ratio of Maximum / Minimum Residual Density ....       3.45 Report
PLAT220_ALERT_2_C NonSolvent   Resd 1  C   Ueq(max)/Ueq(min) Range        3.6 Ratio
PLAT242_ALERT_2_C Low    'MainMol' Ueq as Compared to Neighbors of         K1 Check
PLAT250_ALERT_2_C Large U3/U1 Ratio for <U(i,j)> Tensor(Resd    2)        2.1 Note
PLAT250_ALERT_2_C Large U3/U1 Ratio for <U(i,j)> Tensor(Resd    3)        2.1 Note
PLAT260_ALERT_2_C Large Average Ueq of Residue Including       C1S      0.111 Check
PLAT260_ALERT_2_C Large Average Ueq of Residue Including       C1D      0.114 Check
PLAT341_ALERT_3_C Low Bond Precision on  C-C Bonds ...............    0.00765 Ang.
PLAT414_ALERT_2_C Short Intra D-H..H-X       H1P       ..H5A      .      1.96 Ang.
                                                        x,y,z  =      1_555 Check
PLAT601_ALERT_2_C Unit Cell Contains Solvent Accessible VOIDS   <=         33 Ang**3
PLAT911_ALERT_3_C Missing FCF Refl Between Thmin & STh/L=    0.596         10 Report
                4  0  0,   6 23  0,   9 22  2,   4 23  3,  11 21  3,   1 23  4,
                3 22  6,   3 20  9,   3 18 11,  13  7 14,
```

Alert level G

```
PLAT002_ALERT_2_G Number of Distance or Angle Restraints on AtSite         20 Note
PLAT003_ALERT_2_G Number of Uiso or U(i,j)  Restrained non-H-Atoms         14 Report
PLAT007_ALERT_5_G Number of Unrefined Donor-H Atoms .............          1 Report
              H1P
PLAT111_ALERT_2_G ADDSYM Detects New (Pseudo) Centre of Symmetry .         80 %Fit
PLAT113_ALERT_2_G ADDSYM Suggests Possible Pseudo/New  Space Group       Pnma Check
              WARNING: Disordered Atoms Excluded from Analysis
              Check Model Parameter Symmetry for Reflection Data Support
PLAT171_ALERT_4_G The CIF-Embedded .res File Contains EADP Records         10 Report
PLAT175_ALERT_4_G The CIF-Embedded .res File Contains SAME Records          1 Report
PLAT176_ALERT_4_G The CIF-Embedded .res File Contains SADI Records          2 Report
PLAT177_ALERT_4_G The CIF-Embedded .res File Contains DELU Records          1 Report
PLAT178_ALERT_4_G The CIF-Embedded .res File Contains SIMU Records          1 Report
PLAT187_ALERT_4_G The CIF-Embedded .res File Contains RIGU Records          2 Report
PLAT188_ALERT_3_G A Non-default SIMU Restraint Value has been used     0.0100 Report
PLAT191_ALERT_3_G A Non-default SADI Restraint Value has been used     0.0010 Report
PLAT301_ALERT_3_G Main Residue  Disorder .............(Resd    1)          8% Note
PLAT302_ALERT_4_G Anion/Solvent/Minor-Residue Disorder (Resd    2)       100% Note
PLAT302_ALERT_4_G Anion/Solvent/Minor-Residue Disorder (Resd    3)       100% Note
PLAT303_ALERT_2_G Full Occupancy Atom H20A      with # Connections       2.00 Check
PLAT304_ALERT_4_G Non-Integer Number of Atoms in ..... (Resd    2)       8.54 Check
PLAT304_ALERT_4_G Non-Integer Number of Atoms in ..... (Resd    3)       6.46 Check
PLAT410_ALERT_2_G Short Intra H...H Contact  H47A     ..H48C     .       2.12 Ang.
                                                        x,y,z  =      1_555 Check
PLAT720_ALERT_4_G Number of Unusual/Non-Standard Labels .........           9 Note
              H5AB    H7AB    H9AB    H7SA    H7SB    H7SC    H7DA    H7DB
              H7DC
PLAT722_ALERT_1_G Angle   Calc      112.00, Rep      110.90 Dev...       1.10 Degree
              O2D     -C48D    -H48D       1_555  1_555  1_555     #  336 Check
```

```
PLAT779_ALERT_4_G Suspect or Irrelevant (Bond) Angle(s) in CIF ...      32.10 Deg.
             K1   -C20  -H20A    1_555   1_555   1_555 ......     #  186 Check
PLAT860_ALERT_3_G Number of Least-Squares Restraints ............       1275 Note
PLAT909_ALERT_3_G Percentage of I>2sig(I) Data at Theta(Max) Still        84% Note
PLAT910_ALERT_3_G Missing # of FCF Reflection(s) Below Theta(Min).          4 Note
               1  1  0,   2  0  0,   0  1  1,   1  1  1,
PLAT913_ALERT_3_G Missing # of Very Strong Reflections in FCF ....          1 Note
               4  0  0,
PLAT969_ALERT_5_G The 'Henn et al.' R-Factor-gap value ..........      6.059 Note
             Predicted wR2: Based on SigI**2  2.03 or SHELX Weight 11.49
PLAT978_ALERT_2_G Number C-C Bonds with Positive Residual Density.          2 Info
```

```
   0 ALERT level A = Most likely a serious problem - resolve or explain
   0 ALERT level B = A potentially serious problem, consider carefully
  13 ALERT level C = Check. Ensure it is not caused by an omission or oversight
  29 ALERT level G = General information/check it is not something unexpected

   1 ALERT type 1 CIF construction/syntax error, inconsistent or missing data
  16 ALERT type 2 Indicator that the structure model may be wrong or deficient
  10 ALERT type 3 Indicator that the structure quality may be low
  13 ALERT type 4 Improvement, methodology, query or suggestion
   2 ALERT type 5 Informative message, check
```

It is advisable to attempt to resolve as many as possible of the alerts in all categories. Often the minor alerts point to easily fixed oversights, errors and omissions in your CIF or refinement strategy, so attention to these fine details can be worthwhile. In order to resolve some of the more serious problems it may be necessary to carry out additional measurements or structure refinements. However, the purpose of your study may justify the reported deviations and the more serious of these should normally be commented upon in the discussion or experimental section of a paper or in the "special_details" fields of the CIF. checkCIF was carefully designed to identify outliers and unusual parameters, but every test has its limitations and alerts that are not important in a particular case may appear. Conversely, the absence of alerts does not guarantee there are no aspects of the results needing attention. It is up to the individual to critically assess their own results and, if necessary, seek expert advice.

Publication of your CIF in IUCr journals

A basic structural check has been run on your CIF. These basic checks will be run on all CIFs submitted for publication in IUCr journals (*Acta Crystallographica*, *Journal of Applied Crystallography*, *Journal of Synchrotron Radiation*); however, if you intend to submit to *Acta Crystallographica Section C* or *E* or *IUCrData*, you should make sure that full publication checks are run on the final version of your CIF prior to submission.

Publication of your CIF in other journals

Please refer to the *Notes for Authors* of the relevant journal for any special instructions relating to CIF submission.

PLATON version of 02/02/2025; check.def file version of 02/02/2025

Datablock 25062 - ellipsoid plot

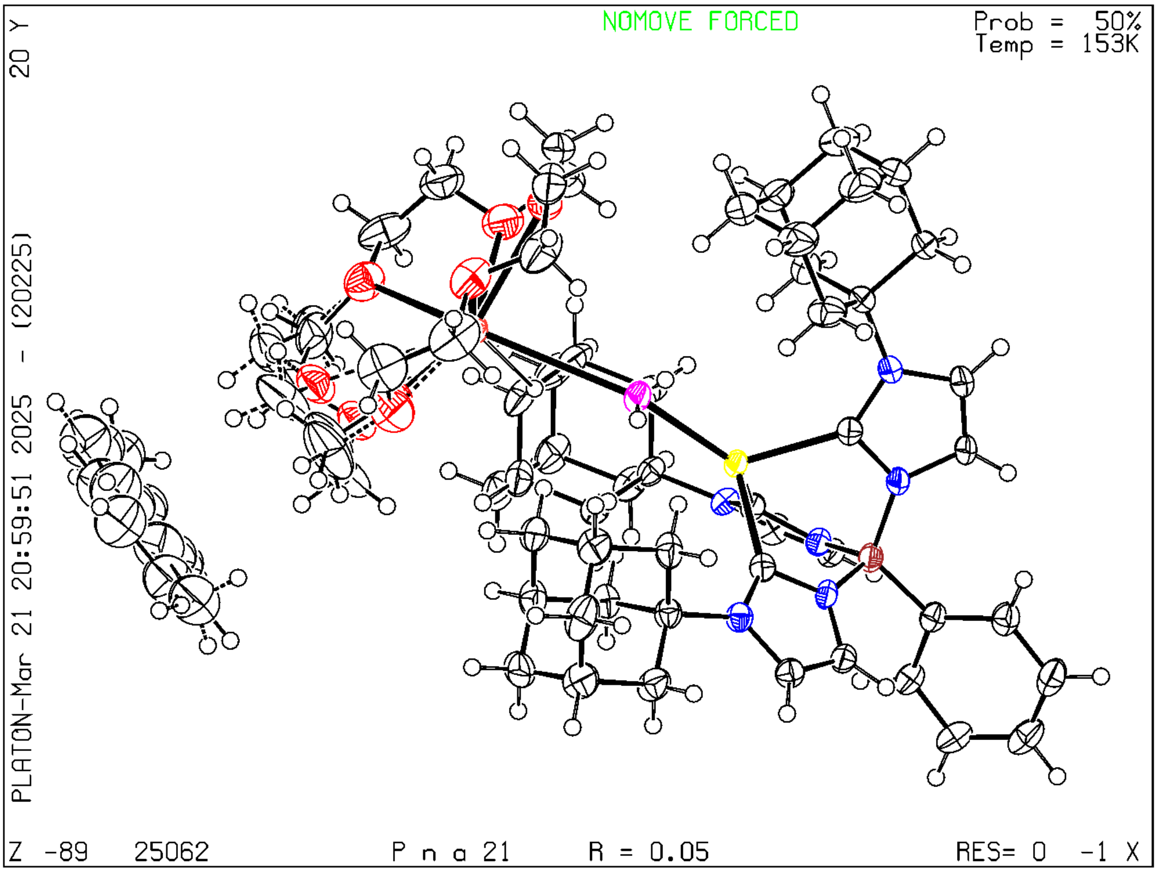

checkCIF/PLATON report

Structure factors have been supplied for datablock(s) 25044

THIS REPORT IS FOR GUIDANCE ONLY. IF USED AS PART OF A REVIEW PROCEDURE FOR PUBLICATION, IT SHOULD NOT REPLACE THE EXPERTISE OF AN EXPERIENCED CRYSTALLOGRAPHIC REFEREE.

No syntax errors found. CIF dictionary Interpreting this report

Datablock: 25044

Bond precision:	C-C = 0.0045 A		Wavelength=0.71073
Cell:	a=13.8644(6)	b=17.6271(7)	c=19.4292(8)
	alpha=90	beta=93.064(2)	gamma=90
Temperature:	173 K		

	Calculated	Reported
Volume	4741.5(3)	4741.5(3)
Space group	P 21/c	P 21/c
Hall group	-P 2ybc	-P 2ybc
Moiety formula	C45 H58 B Fe N6 P, 2(C4 H8 O)	C45 H58 B Fe N6 P, 2(C4 H8 O)
Sum formula	C53 H74 B Fe N6 O2 P	C53 H74 B Fe N6 O2 P
Mr	924.81	924.81
Dx,g cm-3	1.296	1.296
Z	4	4
Mu (mm-1)	0.400	0.400
F000	1984.0	1984.0
F000'	1986.37	
h,k,lmax	17,22,24	17,22,24
Nref	9735	9723
Tmin,Tmax	0.904,0.964	0.690,0.745
Tmin'	0.853	

Correction method= # Reported T Limits: Tmin=0.690 Tmax=0.745
AbsCorr = MULTI-SCAN

Data completeness= 0.999 Theta(max)= 26.399

R(reflections)= 0.0461(8819) wR2(reflections)= 0.1124(9723)

S = 1.067 Npar= 610

The following ALERTS were generated. Each ALERT has the format
test-name_ALERT_alert-type_alert-level.
Click on the hyperlinks for more details of the test.

Alert level G

```
PLAT002_ALERT_2_G Number of Distance or Angle Restraints on AtSite         20 Note
PLAT003_ALERT_2_G Number of Uiso or U(i,j)  Restrained non-H-Atoms         10 Report
PLAT007_ALERT_5_G Number of Unrefined Donor-H Atoms .............           2 Report
              H1PA  H1PB
PLAT083_ALERT_2_G SHELXL Second Parameter in WGHT  Unusually Large       6.27 Why ?
PLAT171_ALERT_4_G The CIF-Embedded .res File Contains EADP Records         10 Report
PLAT175_ALERT_4_G The CIF-Embedded .res File Contains SAME Records          8 Report
PLAT177_ALERT_4_G The CIF-Embedded .res File Contains DELU Records          1 Report
PLAT187_ALERT_4_G The CIF-Embedded .res File Contains RIGU Records          1 Report
PLAT232_ALERT_2_G Hirshfeld Test Diff (M-X)  Fe1      --P1       .        9.2 s.u.
PLAT302_ALERT_4_G Anion/Solvent/Minor-Residue Disorder (Resd    2)       100% Note
PLAT302_ALERT_4_G Anion/Solvent/Minor-Residue Disorder (Resd    3)       100% Note
PLAT302_ALERT_4_G Anion/Solvent/Minor-Residue Disorder (Resd    4)       100% Note
PLAT302_ALERT_4_G Anion/Solvent/Minor-Residue Disorder (Resd    5)       100% Note
PLAT304_ALERT_4_G Non-Integer Number of Atoms in ..... (Resd    2)       8.24 Check
PLAT304_ALERT_4_G Non-Integer Number of Atoms in ..... (Resd    3)      10.11 Check
PLAT304_ALERT_4_G Non-Integer Number of Atoms in ..... (Resd    4)       4.76 Check
PLAT304_ALERT_4_G Non-Integer Number of Atoms in ..... (Resd    5)       2.89 Check
PLAT398_ALERT_2_G Deviating  C-O-C   Angle From 120 for O1S      .      109.0 Degree
PLAT398_ALERT_2_G Deviating  C-O-C   Angle From 120 for O1D      .      109.6 Degree
PLAT411_ALERT_2_G Short Inter H...H Contact  H7A      ..H7DA     .       2.09 Ang.
                                             -x,1/2+y,1/2-z  =      2_555 Check
PLAT411_ALERT_2_G Short Inter H...H Contact  H7A      ..H7DB     .       2.03 Ang.
                                             -x,1/2+y,1/2-z  =      2_555 Check
PLAT411_ALERT_2_G Short Inter H...H Contact  H25A     ..H7DB     .       2.08 Ang.
                                                 -x,1-y,1-z  =      3_566 Check
PLAT720_ALERT_4_G Number of Unusual/Non-Standard Labels .........          34 Note
              H1PA    H1PB    H1SA    H1SB    H2SA    H2SB    H3SA    H3SB
              H4SA    H4SB    H1DA    H1DB    H2DA    H2DB    H3DA    H3DB
              H4DA    H4DB    H5SA    H5SB    H6SA    H6SB    H7SA    H7SB
              H8SA    H8SB    H6DA    H6DB    H5DA    H5DB    H7DA    H7DB
              H8DA    H8DB
PLAT860_ALERT_3_G Number of Least-Squares Restraints ............         809 Note
PLAT870_ALERT_4_G ALERTS Related to Twinning Effects Suppressed ..          ! Info
PLAT910_ALERT_3_G Missing # of FCF Reflection(s) Below Theta(Min).          1 Note
                1  0  0,
PLAT912_ALERT_4_G Missing # of FCF Reflections Above STh/L=  0.600        16 Note
PLAT931_ALERT_5_G CIFcalcFCF  Twin Law         [ 0 0 1] Est.d BASF       0.37 Check
PLAT933_ALERT_2_G Number of HKL-OMIT Records in Embedded .res File          1 Note
                1  0  0,
PLAT941_ALERT_3_G Average HKL Measurement Multiplicity ..........         1.0 Low
PLAT969_ALERT_5_G The 'Henn et al.' R-Factor-gap value ..........       3.666 Note
              Predicted wR2: Based on SigI**2  3.06 or SHELX Weight 10.53
```

0 **ALERT level A** = Most likely a serious problem - resolve or explain
0 **ALERT level B** = A potentially serious problem, consider carefully
0 **ALERT level C** = Check. Ensure it is not caused by an omission or oversight
31 **ALERT level G** = General information/check it is not something unexpected

```
 0 ALERT type 1 CIF construction/syntax error, inconsistent or missing data
10 ALERT type 2 Indicator that the structure model may be wrong or deficient
 3 ALERT type 3 Indicator that the structure quality may be low
15 ALERT type 4 Improvement, methodology, query or suggestion
 3 ALERT type 5 Informative message, check
```

It is advisable to attempt to resolve as many as possible of the alerts in all categories. Often the minor alerts point to easily fixed oversights, errors and omissions in your CIF or refinement strategy, so attention to these fine details can be worthwhile. In order to resolve some of the more serious problems it may be necessary to carry out additional measurements or structure refinements. However, the purpose of your study may justify the reported deviations and the more serious of these should normally be commented upon in the discussion or experimental section of a paper or in the "special_details" fields of the CIF. checkCIF was carefully designed to identify outliers and unusual parameters, but every test has its limitations and alerts that are not important in a particular case may appear. Conversely, the absence of alerts does not guarantee there are no aspects of the results needing attention. It is up to the individual to critically assess their own results and, if necessary, seek expert advice.

Publication of your CIF in IUCr journals

A basic structural check has been run on your CIF. These basic checks will be run on all CIFs submitted for publication in IUCr journals (*Acta Crystallographica*, *Journal of Applied Crystallography*, *Journal of Synchrotron Radiation*); however, if you intend to submit to *Acta Crystallographica Section C* or *E* or *IUCrData*, you should make sure that full publication checks are run on the final version of your CIF prior to submission.

Publication of your CIF in other journals

Please refer to the *Notes for Authors* of the relevant journal for any special instructions relating to CIF submission.

PLATON version of 02/02/2025; check.def file version of 02/02/2025

Datablock 25044 - ellipsoid plot

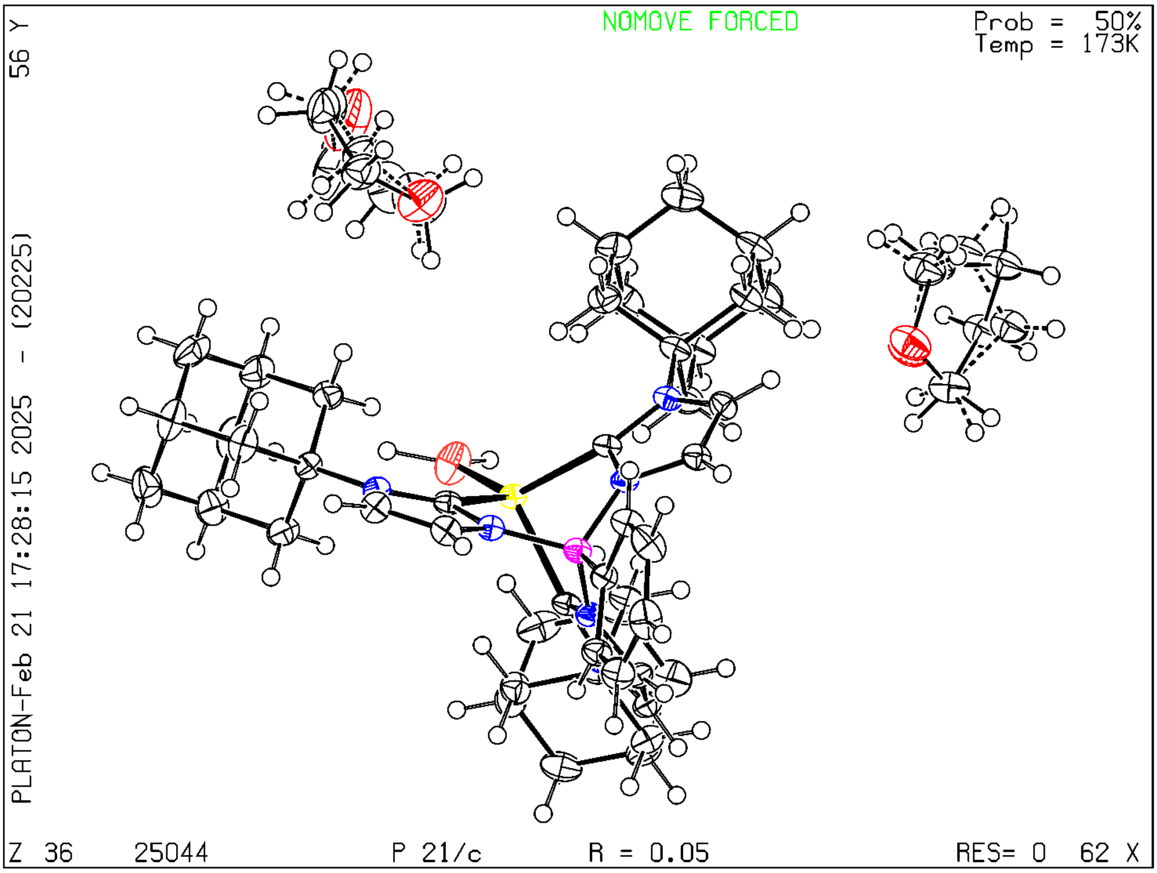

Supplement: Supplementary file 2 — Supporting File 2: anie71880‐sup‐0002‐Data.zip [file ANIE-65-e23239-s001.zip › anie71880-sup-0002-Data/checkcif.pdf]
